# Synthesis of axially chiral gold complexes and their applications in asymmetric catalyses

**DOI:** 10.3762/bjoc.9.261

**Published:** 2013-10-28

**Authors:** Yin-wei Sun, Qin Xu, Min Shi

**Affiliations:** 1 Key Laboratory for Advanced Materials and Institute of Fine Chemicals, School of Chemistry & Molecular Engineering, East China University of Science and Technology, and 130 MeiLong Road, Shanghai 200237, People’s Republic of China,; 2 State Key Laboratory of Organometallic Chemistry, Shanghai Institute of Organic Chemistry, Chinese Academy of Sciences, 354 Fenglin Road, Shanghai 200032, People’s Republic of China

**Keywords:** asymmetric gold catalysis, chiral Au catalysts, gold-π interaction, NHC gold complex

## Abstract

Several novel chiral *N*-heterocyclic carbene and phosphine ligands were prepared from (*S*)-BINOL. Moreover, their ligated Au complexes were also successfully synthesized and characterized by X-ray crystal diffraction. A weak gold-π interaction between the Au atom and the aromatic ring in these gold complexes was identified. Furthermore, we confirmed the formation of a pair of diastereomeric isomers in NHC gold complexes bearing an axially chiral binaphthyl moiety derived from the hindered rotation around C–C and C–N bonds. In the asymmetric intramolecular hydroamination reaction most of these chiral Au(I) complexes showed good catalytic activities towards olefins tethered with a NHTs functional group to give the corresponding product in moderate yields and up to 29% ee.

## Introduction

**Figure 1:**
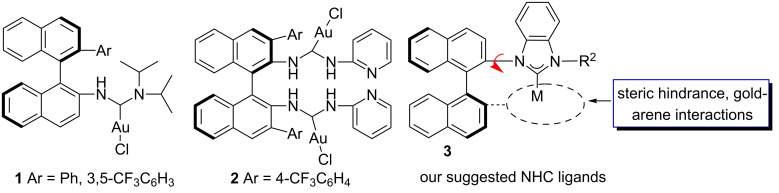
Monodentate chiral NHC gold catalysts in recent years.

After the long-held assumption of the non-reactivity of gold complexes, numerous reactions catalyzed by gold complexes have emerged in the last 2 decades [1–9]. In the past few years, reports on gold-catalyzed organic transformations have increased substantially [10–29]. Homogeneous gold catalysis has proven to be a powerful tool in organic synthesis. However, chiral gold complexes [30–45], especially chiral NHC–gold complex-catalyzed asymmetric reactions [46–53] are still uncommon. Very few efficient chiral NHC–gold catalysts have been known up to the year of 2013. So far, several axially chiral NHC–gold catalysts based on binaphthyl skeleton such as **1** and **2** [46,49] have been reported with good to excellent chiral inductions in asymmetric gold catalysis (Figure 1). Encouraged by these results, we attempted to develop novel types of axially chiral NHC–gold catalysts based on the binaphthyl skeleton.

Very recently, Echavarren’s group has reported a very important gold–arene interaction in dialkylbiarylphosphane gold complexes, which is very useful in gold catalysis [54]. It has been disclosed that there was a weak gold-π interaction between the gold atom and the aromatic ring in catalysts **1** [46]. On the basis of this finding, we envisaged that if an aryl group is introduced near the ligated gold atom, the gold–arene interaction may affect the catalytic efficiency in gold catalysis (Figure 1).

## Results and Discussion

**Scheme 1:**
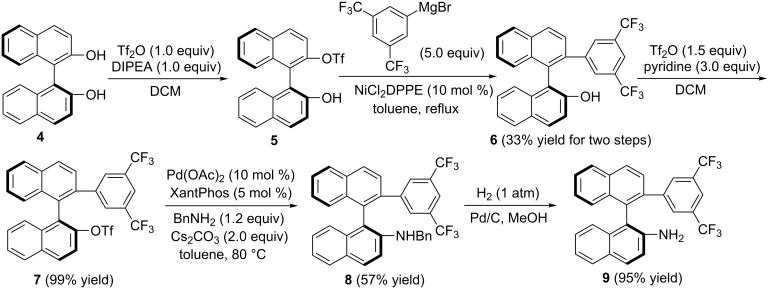
Synthesis of compound **9**.

**Synthesis of the carbene–Au(I) complexes**. The synthesis of compound **9** was reported by Slaughter and co-workers (Scheme 1) [49]. The usage of (*S*)-BINOL as the starting material to react with trifluoromethanesulfonic anhydride in the presence of DIPEA afforded at 0 °C in dichloromethane the corresponding product (*S*)-2'-hydroxy-1,1'-binaphthyl-2-yl trifluoromethanesulfonate (**5**) in good yield. The crude product and NiCl_2_(dppe) (10 mol %) was dissolved in toluene under argon. To this solution was added dropwise a 1.0 M THF solution of 3,5-bis(trifluoromethyl)phenylmagnesium bromide, which afforded (*S*)-**6** in 33% yield in two steps under reflux [55]. Then, (*S*)-**7** was obtained by treatment of (*S*)-**6** with Tf_2_O and pyridine in DCM in 99% yield. The usage of dimethylbis(diphenylphosphino)xanthene (XantPhos) as a ligand and Pd_2_(dba)_3_ as a catalyst in the presence of Cs_2_CO_3_, facilitated the reaction of (*S*)-**7** with benzylamine in toluene to give the desired compound (*S*)-**8** in 57% yield [49]. Reduction of (*S*)-**8** by using Pd/C and H_2_ in MeOH produced the desired compound (*S*)-**9** in 95% yield.

**Scheme 2:**
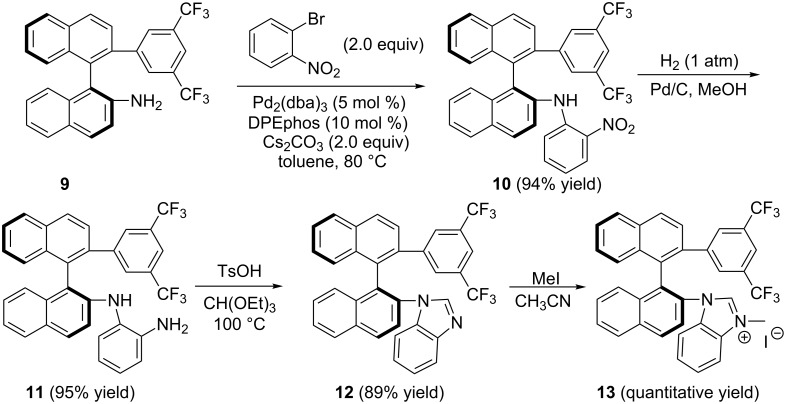
Synthesis of *N*-heterocyclic carbene precursor.

**Scheme 3:**
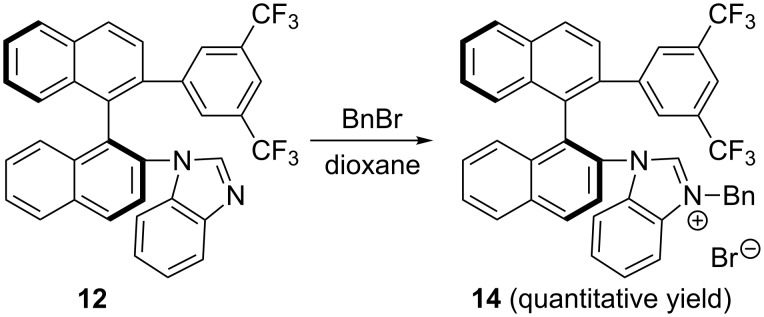
Synthesis of benzimidazolium salt (*S*)-**14**.

The preparation of chiral benzimidazolium salt (*S*)-**13** is shown in Scheme 2. Based on our previous work [52], the coupling reaction between compound (*S*)-**9** and 1-bromo-2-nitrobenzene was carried out by using Pd_2_(dba)_3_ as the catalyst in the presence of bis[2-(diphenylphosphino)phenyl] ether (DPEphos) and Cs_2_CO_3_, affording the desired compound (*S*)-**10** in 94% yield [51]. Reduction of (*S*)-**10** was performed under H_2_ (1.0 atm) atmosphere by using Pd/C as a catalyst, giving the desired compound (*S*)-**11** in 95% yield. The subsequent cyclization of (*S*)-**11** with triethyl orthoformate was carried out at 100 °C in the presence of *p*-toluenesulfonic acid, affording the desired product (*S*)-**12** in 89% yield. The corresponding benzimidazolium salt (*S*)-**13** was obtained in quantitative yield upon treating the benzimidazole ring of (*S*)-**12** with methyl iodide in acetonitrile under reflux (Scheme 2). Moreover, treatment of the benzimidazole ring of (*S*)-**12** by using benzyl bromide upon heating in dioxane could produce the corresponding benzimidazolium salt (*S*)-**14** also in quantitative yield (Scheme 3).

**Scheme 4:**
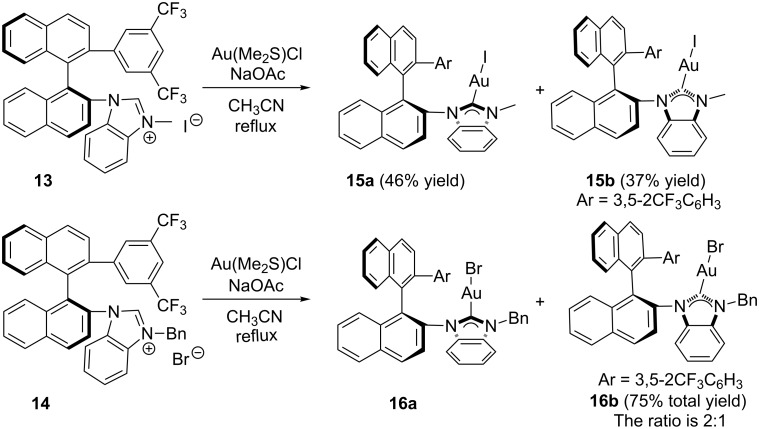
Synthesis of carbene Au complexes.

**Figure 2:**
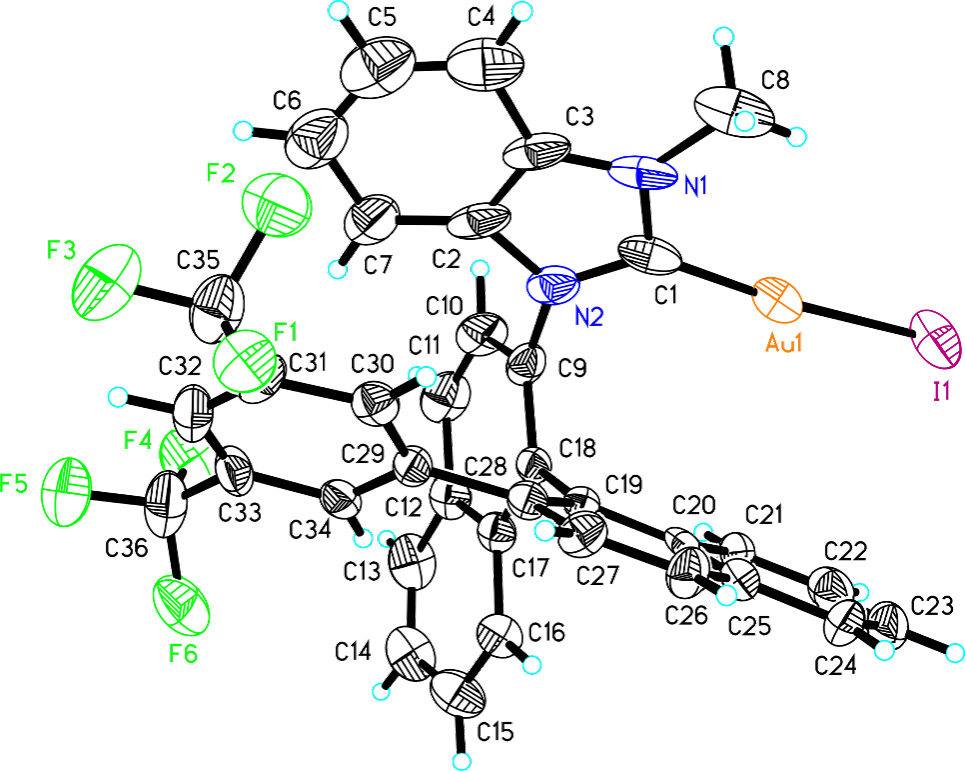
The crystal data of gold complex (*S*)-**15a** was deposited in the CCDC with the number 883917. Empirical formula: C_36_H_22_AuF_6_IN_2_; formula weight: 920.42; crystal color, colorless; crystal dimensions: 0.321 × 0.212 × 0.143 mm; crystal system: orthorhombic; lattice parameters: *a* = 9.6909(5) Å, *b* = 18.5814(9) Å, *c* = 36.0427(18) Å, α = 90^o^, β = 90^o^, γ = 90^o^, *V* = 6490.2(6) Å^3^; space group: *P*2(1)2(1)2(1); *Z* = 8; *D*_calc_ = 1.884 g/cm^3^; F_000_ = 3504; final R indices [I > 2sigma(I)]: R1 = 0.0421; wR2 = 0.0793.

**Figure 3:**
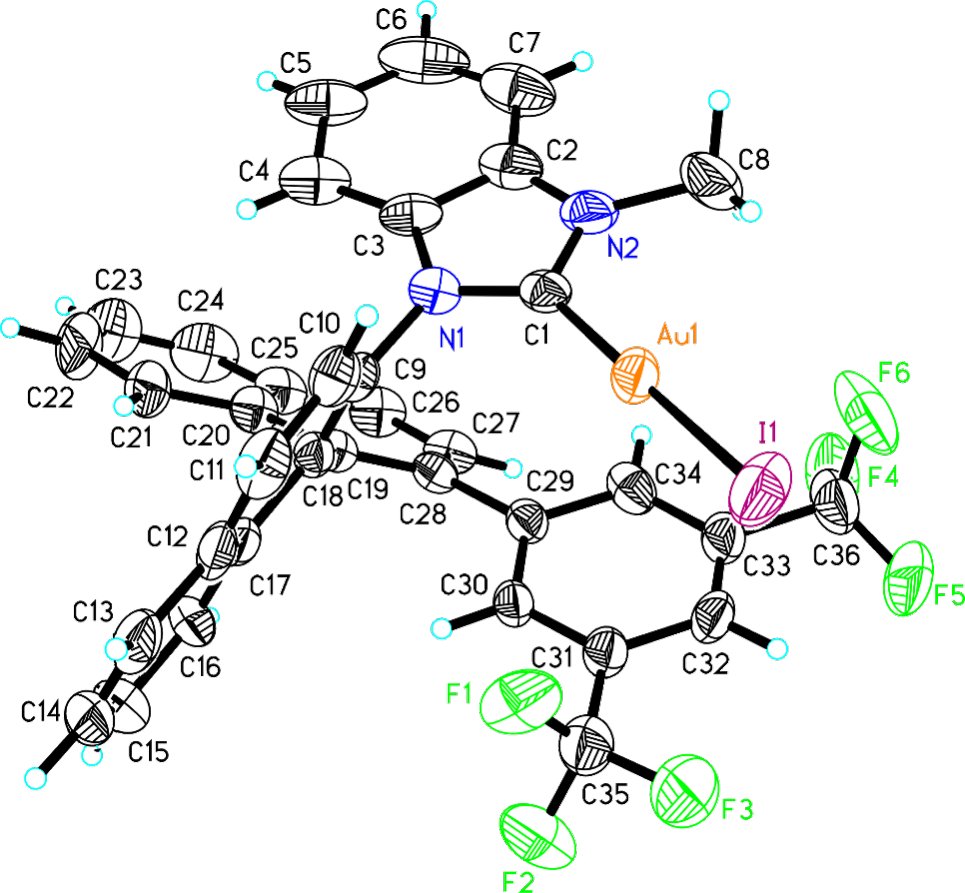
The crystal data of gold complex (*S*)-**15b** was deposited in the CCDC with the number 883916. Empirical formula: C_36_H_22_AuF_6_IN_2_; formula weight: 920.42; crystal color, colorless; crystal dimensions: 0.265 × 0.211 × 0.147 mm; crystal system: orthorhombic; lattice parameters: *a* = 7.6103(5) Å, *b* = 12.6408(8) Å, *c* = 34.029(2) Å, α = 90^o^, β = 90^o^, γ = 90^o^, *V* = 3273.6(4) Å^3^; space group: *P*2(1)2(1)2(1); *Z* = 4; *D*_calc_ = 1.868 g/cm^3^; F_000_ = 1752; final R indices [I > 2sigma(I)]: R1 = 0.0482; wR2 = 0.1072.

**Scheme 5:**
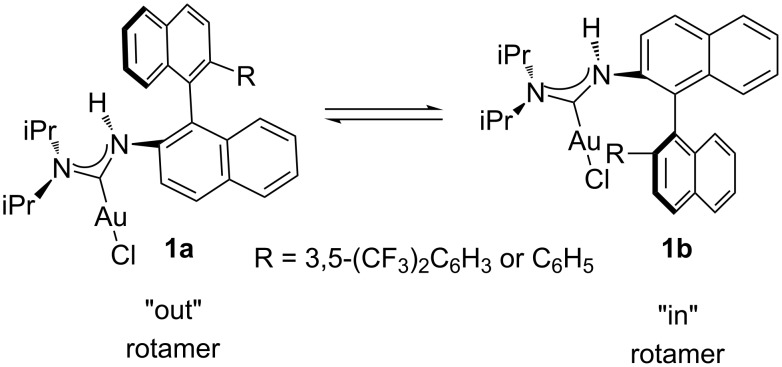
Rotamers of **1a** and **1b** by DFT calculation reported by Slaughter’s group.

With these NHC precursor salts (*S*)-**13** and (*S*)-**14** in hand, their coordination pattern with Au was examined. Benzimidazolium salts (*S*)-**13** and (*S*)-**14** were treated with AuCl·S(Me)_2_ in acetonitrile in the presence of NaOAc under reflux, giving the corresponding Au complexes (*S*)-**15** [two diastereomers: (*S*)-**15a** in 46% yield and (*S*)-**15b** in 37% yield] and (*S*)-**16** in 75% total yield [the two diastereomers: (*S*)-**16a** and (*S*)-**16b** can not be separated by silica gel column chromatography] as a white solid after purification with silica gel column chromatography (Scheme 4). The ratio of (*S*)-**16a** and (*S*)-**16b** was identified as 1:2 on the basis of ^1^H NMR spectroscopic data. After recrystallization from the mixed solvent of DCM and pentane, the single crystals of diastereomers (*S*)-**15a** and (*S*)-**15b** were obtained and their structures were confirmed by the X-ray crystal structure diffraction (Figure 2 and Figure 3). The distance between the center of the aromatic ring in one naphthyl moiety (C20–C25) and the Au atom in (*S*)-**15a** was only 3.7 Å (Figure 2). The distance from the Au atom to the center of the bis(trifluoromethyl)phenyl ring (C29–C34) in (*S*)-**15b** was 3.5 Å (Figure 3). Thus, their X-ray crystal structures clearly revealed the presence of a weak gold–π interaction between the Au atom and the aromatic rings in these gold complexes. Because of the gold–π interaction, the C–N bond could not rotate freely, giving two diastereomeric rotamers (*S*)-**15a** and (*S*)-**15b**. Slaughter and co-workers have also found two rotamers in gold complexes **1** caused by the handicap of C–N bond rotation on the basis of X-ray diffraction and named them as “out” rotamer and “in” rotamer [49] (Scheme 5). Their energy barrier has been also disclosed by DFT calculations.

**Scheme 6:**
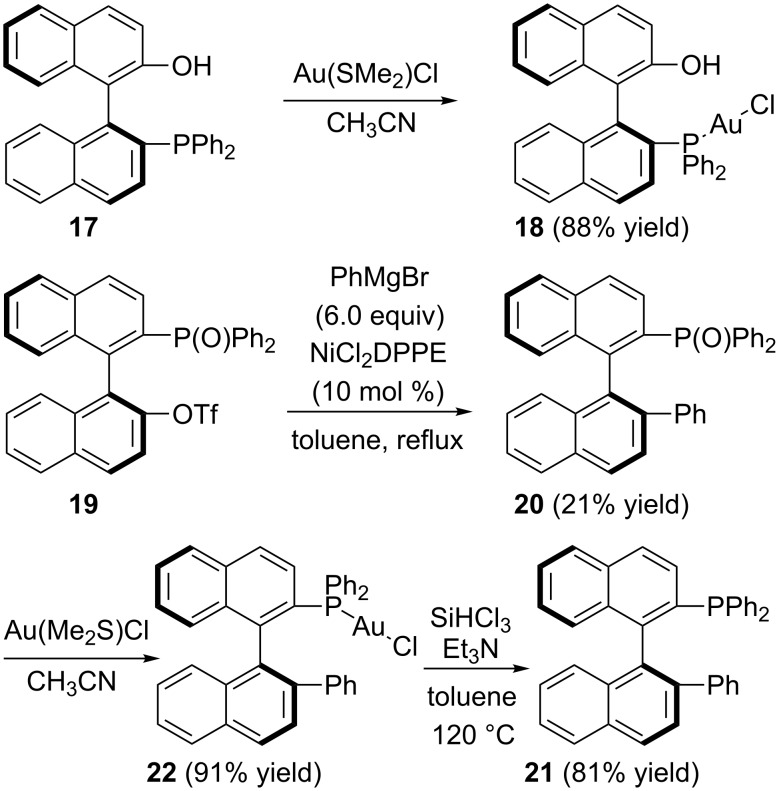
The synthesis of P–Au complexes.

**Figure 4:**
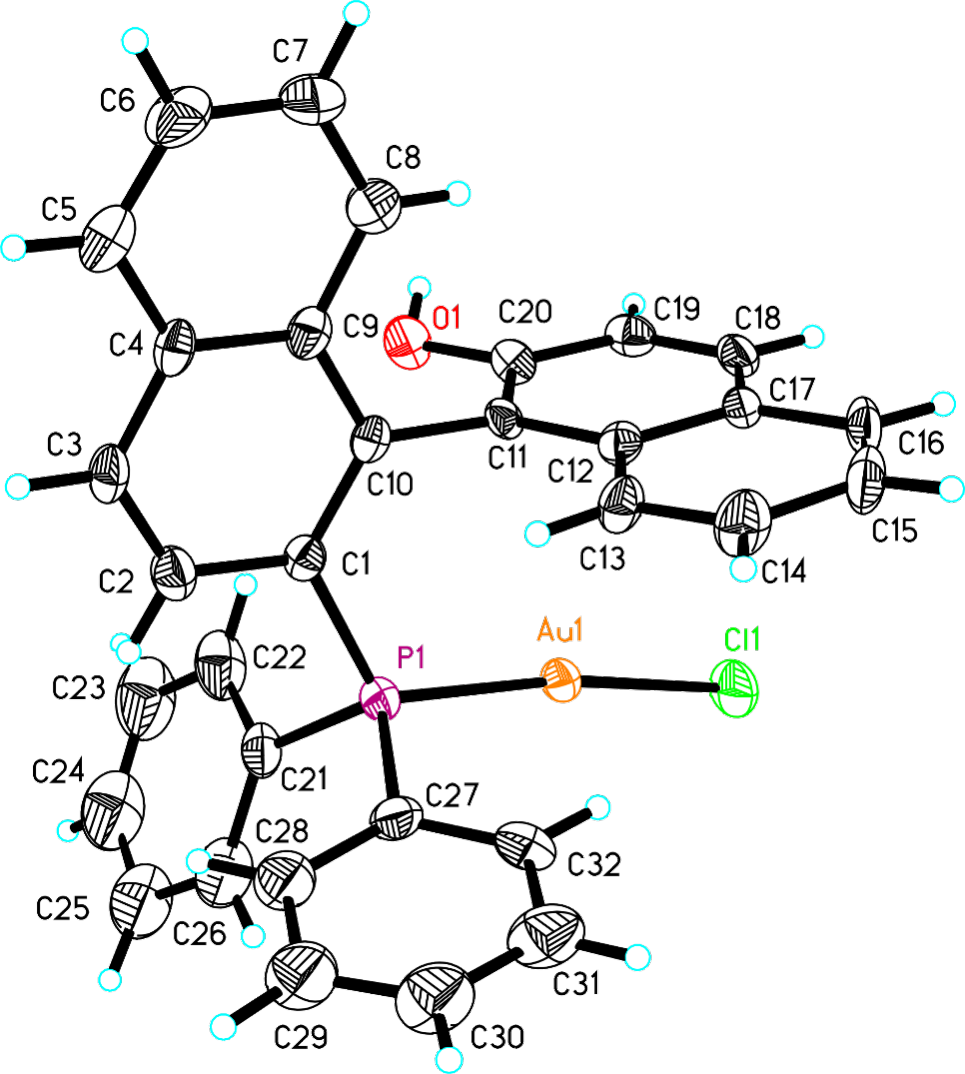
The crystal data of gold complex (*S*)-**18** was deposited in the CCDC with the number 920617. Empirical formula: C_32_H_23_AuClOP; formula weight: 686.89; crystal color, colorless; crystal dimensions: 0.212 × 0.139 × 0.101 mm; crystal system: orthorhombic; lattice parameters: *a* = 9.0411(7) Å, *b* = 13.4833(10) Å, *c* = 21.9878(16) Å, α = 90^o^, β = 90^o^, γ = 90^o^, *V* = 2680.4(3) Å^3^; space group: *P*2(1)2(1)2(1); *Z* = 4; *D*_calc_ = 1.702 g/cm^3^; F_000_ = 1336; final R indices [I > 2sigma(I)]: R1 = 0.0378; wR2 = 0.0680.

**Synthesis of the P–Au(I) complexes**. The synthesis of the Au complexes (*S*)-**18** and (*S*)-**22** is shown in Scheme 6. Compounds (*S*)-**17** and (*S*)-**19** were prepared according to published literature procedures [56]. Compound (*S*)-**17** was treated with AuCl·S(Me)_2_ in acetonitrile at room temperature to give the corresponding Au complex (*S*)-**18** in 88% yield as a white solid after purification with silica gel column chromatography. The structure of (*S*)-**18** was confirmed by the X-ray crystal structure diffraction (Figure 4). The distance from the Au atom to the center of the aromatic ring (C11, C12 and C17–C20) in one naphthyl moiety was 3.3 Å.

**Figure 5:**
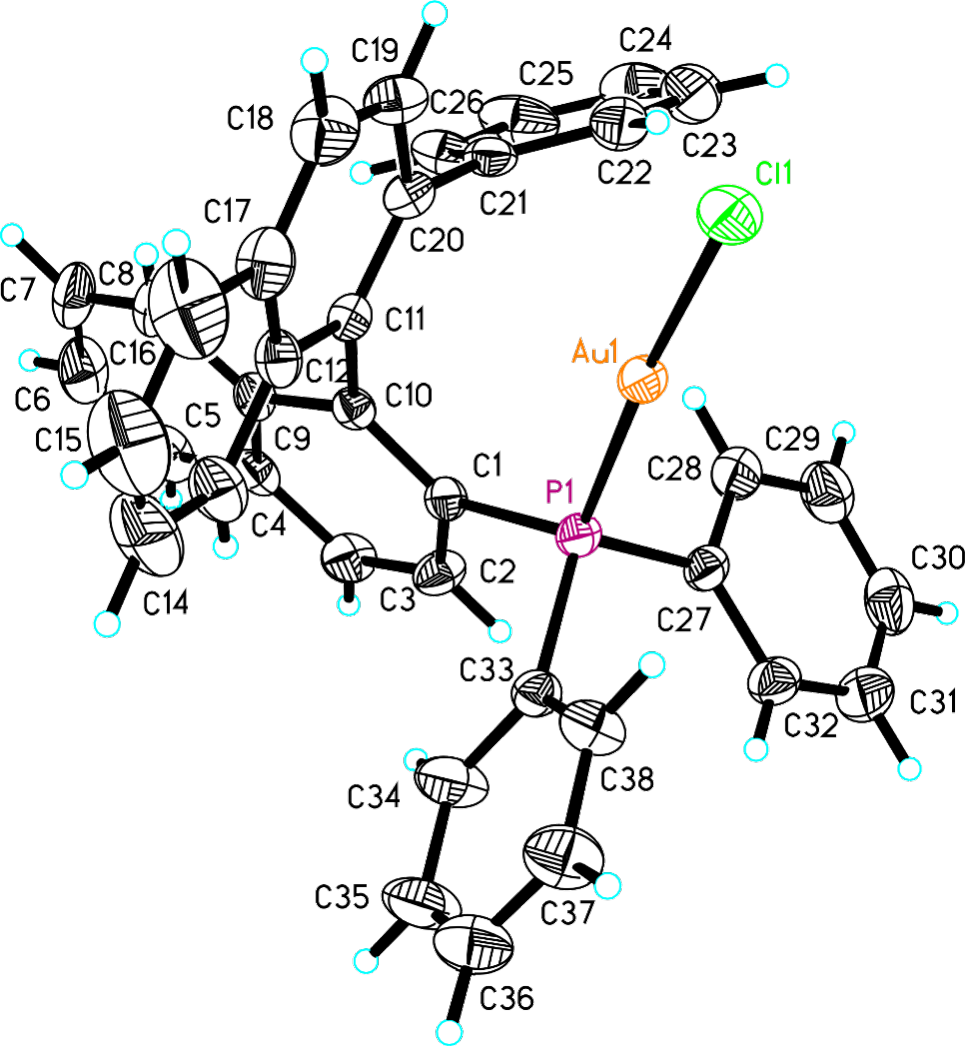
The crystal data of gold complex (*S*)-**22** was deposited in the CCDC with the number 928664. Empirical formula: C_38_H_27_AuClP; formula weight: 746.98; crystal color, habit: yellow; crystal system: monoclinic; crystal size: 0.21 × 0.19 × 0.11; lattice parameters: *a* = 10.1476(9) Å, *b* = 15.7426(14) Å, *c* = 10.5615(9) Å, α = 90^o^, β = 115.257(2)^o^, γ = 90^o^, *V* = 1525.9(2) Å^3^; space group: *P*2(1); *Z* = 4; *D*_calc_ = 1.626 g/cm^3^; F_000_ = 732; final R indices [I > 2sigma(I)]: R1 = 0.0231; wR2 = 0.0538.

The compound (*S*)-**19** and NiCl_2_(dppe) (10 mol %) were dissolved in toluene under argon. To this solution was added dropwise a 1.0 M THF solution of phenylmagnesium bromide and the desired compound (*S*)-**20** was afforded in 21% yield. Then, the obtained compound (*S*)-**20** was treated with SiHCl_3_ in the presence of triethylamine in toluene at 120 °C, giving (*S*)-diphenyl(2'-phenyl-1,1'-binaphthyl-2-yl)phosphine (**21**) in 81% yield. The corresponding gold complex (*S*)-**22** was obtained in 91% yield upon treating (*S*)-**21** with the same method as the gold complex (*S*)-**18**. The structure of (*S*)-**22** was confirmed by X-ray crystal structure diffraction (Figure 5). The crystal structure of (*S*)-**22** (Figure 5) revealed that the distance from the Au atom to the center of the phenyl ring (C21–C26) was 4.5 Å. During the process of the preparation of (*S*)-**21**, we found a small amount of naphtho[1,2-*g*]chrysene (**23**), presumably derived from a cross coupling of compound (*S*)-**19** with PhMgBr. Its structure was also confirmed by the X-ray crystal structure diffraction (Figure SI-1 in Supporting Information File 1).

The catalytic activities of these gold complexes were examined by the gold-catalyzed asymmetric intramolecular hydroamination of olefin **24** tethered with a NHTs functional group.

**Intramolecular hydroamination reaction catalyzed by Au(I) complexes**. We synthesized a variety of Au complexes both neutral and cationic and subsequently used these complexes as catalysts in a variety of reactions. High enantioselectivities were achieved in the asymmetric intramolecular hydroamination of allenes by using a variety of chiral phosphine–Au(I) complexes [57–63]. On the other hand, the intramolecular hydroamination of olefins is a more important reaction in organic synthesis and has been widely reported [64–68]. Recently, the enantioselective intramolecular hydroamination of olefins has also been significantly improved by using various transition metal complexes or other metal complexes [69–77]. However, to the best of our knowledge, the enantioselective intramolecular hydroamination of olefins catalyzed by gold complexes has not been reported yet. We therefore applied our Au complexes to the asymmetric catalysis of the intramolecular hydroamination of olefin **24** tethered with a NHTs functional group.

Treatment of olefin **24** with the axially chiral gold complex (*S*)-**22** and AgOTf (5 mol %) in toluene at 85 ^o^C for 36 h afforded pyrrolidine derivative **25** in 46% yield and 17% ee. While using AgSbF_6_ or AgNTf_2_ as additives, only trace amounts of **25** were formed. Further screening of silver salts revealed that AgOTs showed the best catalytic activity in this reaction, giving **25** in 72% yield and 27% ee (Table 1, entries 1–6). The usage of other solvents such as DCE, CH_3_CN and THF, decreased significantly the yield (Table 1, entries 7–9). The employment of other axially chiral Au complexes in this reaction led to similar results, affording **25** in 42–65% yields and 7–2–7% ee (Table 1, entries 10–13). The control experiment indicated that no reaction occurred in the absence of a Au catalyst (Table 1, entry 14).

**Table 1:** Asymmetric intramolecular hydroamination catalyzed by Au complexes.

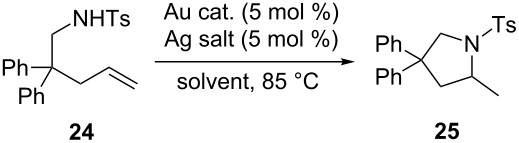					

entry^a^	Au cat.	Ag salt	solvent	yield (%)^b^	ee (%)^c^

1	**22**	AgOTf	toluene	46	17
2	**22**	AgSbF_6_	toluene	trace	–^d^
3	**22**	CF_3_COOAg	toluene	69	11
4	**22**	AgOTs	toluene	72	27
5	**22**	AgBF_4_	toluene	15	10
6	**22**	AgNTf_2_	toluene	48	0
7	**22**	AgOTs	CH_3_CN	12	27
8	**22**	AgOTs	DCE	11	29
9	**22**	AgOTs	THF	N.R.	–^d^
10	**18**	AgOTs	toluene	58	24
11	**16**	AgOTs	toluene	65	27
12	**15a**	AgOTs	toluene	42	7
13	**15b**	AgOTs	toluene	51	10
14	none	AgOTs	toluene	N.R.	–^d^

^a^The reaction was carried out on a 0.1 mmol scale in solvents (1.0 mL). ^b^Isolated yield. ^c^Measured by chira HPLC. ^d^Not determined.

## Conclusion

Axially chiral Au(I) complexes exhibiting a binaphthalene scaffold with NHC or phosphine gold complexes on one side and an arene moiety on another side were prepared starting from axially chiral BINOL. A weak gold–π interaction between the Au atom and the aromatic ring in these gold complexes was identified. These axially chiral Au(I) complexes showed moderate catalytic activities along with low chiral inductions in the asymmetric intramolecular hydroamination reaction of olefin **24** tethered with a functional group of NHT.

## Experimental

### Synthesis of NHC–Au(I) complexes (*S*)-**15a** and (*S*)-**15b**

The compound (*S*)-**13** (145 mg, 0.2 mmol) and AuCl·S(Me)_2_ (59 mg, 0.2 mmol), NaOAc (33 mg, 0.4 mmol) were heated under reflux in CH_3_CN (2 mL) overnight. The volatiles were then removed under reduced pressure and the residue was purified by a silica gel flash column chromatography to afford gold-complexes (*S*)-**15a** (84 mg) in 46% yield and (*S*)-**15b** (68 mg) in 37% yield. A single crystal grown from complex (*S*)-**15a** or (*S*)-**15b** in a saturated solution of CH_2_Cl_2_/pentane was suitable for X-ray crystal analysis. (*S*)-**15a**: white solid; [α]_D_^20^ −64.7 (*c* 0.10, CH_2_Cl_2_); ^1^H NMR (400 MHz, CDCl_3_, TMS) δ 8.17–8.13 (m, 2H, ArH), 7.94 (d, *J* = 8.8 Hz, 1H, ArH), 7.90–7.88 (m, 1H, ArH), 7.74–7.70 (m, 1H, ArH), 7.66–7.59 (m, 3H, ArH), 7.56–7.50 (m, 2H, ArH), 7.42 (d, *J* = 8.4 Hz, 1H, ArH), 7.34–7.28 (m, 4H, ArH), 7.06 (s, 2H, ArH), 6.86–6.82 (m, 1H, ArH), 5.60 (d, *J* = 8.4 Hz, 1H, ArH), 3.78 (s, 3H, CH_3_); ^19^F NMR (376 MHz, CDCl_3_, CFCl_3_) δ −63.096; ^13^C NMR (100 MHz, CDCl_3_) δ 141.5, 140.8, 134.9, 134.5, 133.4, 132.9, 132.6, 131.3, 131.0, 130.9, 130.7, 129.9, 129.2, 129.1, 129.0, 128.5, 128.41, 128.37, 127.9, 127.6, 127.3, 126.9, 126.8, 126.4, 123.7, 123.4, 121.0, 120.6, 113.2, 111.4, 34.8; IR (CH_2_Cl_2_) ν: 3059, 2926, 1594, 1385, 1346, 1277, 1182, 1133, 897, 820, 745, 713 cm^−1^; HRMS–ESI: [M + NH_4_]^+^ calcd for C_36_H_26_AuF_6_IN_3_, 938.0736; found, 938.0728. (*S*)-**15b**: white solid; [α]_D_^20^ −66.1 (*c* 1.45, CH_2_Cl_2_); ^1^H NMR (400 MHz, CDCl_3_, TMS) δ 8.17 (d, *J* = 8.4 Hz, 1H, ArH), 8.13 (d, *J* = 8.0 Hz, 1H, ArH), 7.85 (d, *J* = 8.0 Hz, 1H, ArH), 7.79–7.69 (m, 4H, ArH), 7.63 (d, *J* = 8.0 Hz, 1H, ArH), 7.59–7.54 (m, 3H, ArH), 7.50–7.46 (m, 1H, ArH), 7.23 (d, *J* = 8.8 Hz, 2H, ArH), 7.17 (s, 2H, ArH), 7.08–7.04 (m, 1H, ArH), 6.47–6.42 (m, 2H, ArH), 3.94 (s, 3H, CH_3_); ^19^F NMR (376 MHz, CDCl_3_, CFCl_3_) δ −62.451; ^13^C NMR (100 MHz, CDCl_3_) δ 134.6, 134.5, 134.1, 134.0, 133.6, 131.4, 131.34, 131.27, 129.1, 129.0, 128.8, 128.7, 128.66, 128.58, 128.5, 128.3, 128.2, 127.8, 126.9, 126.5, 124.1, 123.4, 118.6, 31.9; IR (CH_2_Cl_2_) ν: 2923, 2851, 1726, 1465, 1387, 1277, 1181, 1131, 894, 823, 743, 712, 681 cm^−1^; HRMS–ESI: [M + NH_4_]^+^ calcd for C_36_H_26_AuF_6_IN_3_, 938.0736; found, 938.0725.

### Synthesis of chiral P–Au(I) complexes (*S*)-**18** and (*S*)-**22**

The compound (*S*)-**17** (454 mg, 1.0 mmol) and AuCl·S(Me)_2_ (294 mg, 1.0 mmol) were stirred in CH_3_CN (10 mL) overnight. The volatiles were then removed under reduced pressure and the residue was purified by silica gel flash column chromatography to afford gold-complex (*S*)-**18** (603 mg) in 88% yield. A single crystal grown from complex (*S*)-**18** in a saturated solution of CH_2_Cl_2_/pentane was suitable for X-ray crystal analysis. (*S*)-**18**: white solid; [α]_D_^20^ −35.4 (*c* 0.20, CH_2_Cl_2_); ^1^H NMR (400 MHz, CDCl_3_, TMS) δ 7.99–7.93 (m, 3H, ArH), 7.80 (d, *J* = 8.4 Hz, 1H, ArH), 7.60–7.56 (m, 1H, ArH), 7.50–7.45 (m, 3H, ArH), 7.42–7.17 (m, 12H, ArH), 6.86–6.82 (m, 1H, ArH), 6.45 (d, *J* = 8.4 Hz, 1H, ArH), 5.17 (br, 1H, OH); ^31^P NMR (162 MHz, CDCl_3_, 85% H_3_PO_4_) δ 26.116; ^13^C NMR (100 MHz, CDCl_3_) δ 141.8, 136.5, 134.4, 133.7, 133.24, 133.22, 133.1, 132.39, 132.35, 130.5, 130.2, 129.8, 129.0, 128.9, 128.6, 128.4, 127.6, 127.3, 127.2, 127.1, 127.0, 126.6, 124.2, 123.7, 123.0, 112.6, 110.4; IR (CH_2_Cl_2_) ν: 3359, 3055, 2924, 1623, 1513, 1435, 1269, 1098, 972, 937, 814, 743, 692 cm^−1^; HRMS–ESI: [M + NH_4_]^+^: calcd for C_32_H_27_AuClNOP, 704.1179; found, 704.1170.

Gold complex (*S*)-**22** has been prepared by the same reaction procedure as gold complex (*S*)-**18** in 91% yield. A single crystal grown from complex (*S*)-**22** in a saturated solution of CH_2_Cl_2_/pentane was suitable for X-ray crystal analysis. white solid; [α]_D_^20^ −80.7 (*c* 0.95, CH_2_Cl_2_); ^1^H NMR (400 MHz, CDCl_3_, TMS) δ 8.27 (d, *J* = 8.8 Hz, 1H, ArH), 8.05 (d, *J* = 8.4 Hz, 1H, ArH), 7.90 (d, *J* = 8.0 Hz, 1H, ArH), 7.84 (d, *J* = 8.4 Hz, 1H, ArH), 7.67 (d, *J* = 8.4 Hz, 1H, ArH), 7.62–7.57 (m, 1H, ArH), 7.47 (t, *J* = 7.6 Hz, 1H, ArH), 7.40–7.35 (m, 4H, ArH), 7.28–7.24 (m, 2H, ArH), 7.22–7.12 (m, 6H, ArH), 6.97 (d, *J* = 7.6 Hz, 2H, ArH), 6.92 (t, *J* = 7.2 Hz, 2H, ArH), 6.88 (d, *J* = 7.6 Hz, 1H, ArH), 6.84 (d, *J* = 8.8 Hz, 1H, ArH), 6.77 (t, *J* = 7.6 Hz, 2H, ArH); ^31^P NMR (162 MHz, CDCl_3_, 85% H_3_PO_4_) δ 22.898, 22.825, 22.751, 22.697; ^13^C NMR (100 MHz, CDCl_3_) δ 151.4, 134.6, 134.4, 134.1, 133.9, 133.7, 133.5, 133.2, 133.1, 131.5, 131.44, 131.37, 131.35, 131.25, 129.9, 129.3, 129.04, 128.98, 128.92, 128.89, 128.87, 128.85, 128.84, 128.7, 128.64, 128.59, 128.51, 128.4, 128.3, 128.2, 127.8, 127.5, 126.81, 126.80, 126.6, 126.5, 126.47, 126.1, 126.0, 124.1, 123.4; IR (CH_2_Cl_2_) ν: 3054, 1589, 1494, 1480, 1436, 1306, 1265, 1098, 1027, 819, 763, 744, 698 cm^−1^; HRMS–ESI: [M + NH_4_]^+^ calcd for C_38_H_31_AuClNP, 764.1543; found, 764.1532.

### General procedure for the intramolecular hydroamination reaction catalyzed by Au(I) complexes

In a similar way as described in reference [51], a mixture of Au catalyst (5 mol %) and AgX (5 mol %) in solvent (0.5 mL) was stirred at room temperature for 5 min under argon, then a solution of compound **24** (39.1 mg, 0.10 mmol) in solvent (0.5 mL) was added into the resulting solution. The resulting suspension was stirred at 85 °C for 36 h. Column chromatography of the reaction mixture gave the desired product. The enantiomeric purity of the product was determined by chiral HPLC analysis. Compound **25**: ^1^H NMR (400 MHz, CDCl_3_, TMS) δ 7.61 (d, *J* = 8.0 Hz, 2H, ArH), 7.27–7.09 (m, 12H, ArH), 4.17 (d, *J* = 10.4 Hz, 1H, CH_2_), 3.94 (dd, *J*_1_ = 10.4 Hz, *J*_2_ = 0.4 Hz, 1H, CH_2_), 3.82–3.74 (m, 1H, CH), 2.78 (ddd, *J*_1_ = 12.4 Hz, *J*_2_ = 7.2 Hz, *J*_3_ = 0.4 Hz, 1H, CH_2_), 2.38 (s, 3H, CH_3_), 2.26 (dd, *J*_1_ = 12.4 Hz, *J*_2_ = 7.2 Hz, 1H, CH_2_), 1.25 (d, *J* = 6.4 Hz, 3H, CH_3_); ^13^C NMR (100 MHz, CDCl_3_) δ 145.6, 144.8, 142.9, 135.3, 129.5, 128.43, 128.42, 127.1, 126.6, 126.42, 126.39, 126.2, 58.3, 55.4, 52.2, 45.9, 22.1, 21.4; [α]_D_^20^ 20.1 (*c* 1.2, CH_2_Cl_2_), for 29% ee; Chiralcel PA-2, hexane/iPrOH = 60/40, 0.5 mL/min, 214 nm, *t*_major_ = 45.07 min, *t*_minor_ = 27.49 min.

## Supporting Information

File 1 Experimental procedures and characterization date of compounds.

File 2 Chemical information file of compound (*S*)-**15a**.

File 3 Chemical information file of compound (*S*)-**15b**.

File 4 Chemical information file of compound (*S*)-**18**.

File 5 Chemical information file of compound (*S*)-**21**.

File 6 Chemical information file of compound (*S*)-**23**.
