# Supplementary material for: Synthesis of axially chiral gold complexes and their applications in asymmetric catalyses
Source: Beilstein J Org Chem. 2013 Oct 28;9:2224–32. doi: 10.3762/bjoc.9.261 (PMC3817475; doi:10.3762/bjoc.9.261)
Supplement: File 1 — Experimental procedures and characterization date of compounds. [file Beilstein_J_Org_Chem-09-2224-s001.pdf]

Supporting Information

for

Synthesis of axially chiral gold complexes and their applications in asymmetric catalyses

Yin-wei Sun[1], Qin Xu[1]* and Min Shi[1,2]*

Address: [1]Key Laboratory for Advanced Materials and Institute of Fine Chemicals, School of Chemistry & Molecular Engineering, East China University of Science and Technology, and 130 MeiLong Road, Shanghai 200237, People's Republic of China, and [2]State Key Laboratory of Organometallic Chemistry, Shanghai Institute of Organic Chemistry, Chinese Academy of Sciences, 354 Fenglin Road, Shanghai 200032, People's Republic of China

Email: Qin Xu - qinxu@ecust.edu.cn; Min Shi - Mshi@mail.sioc.ac.cn

*Corresponding author

Experimental procedures and characterization date of compounds

CONTENTS

General remarks……………………………………………………………………………S2

The synthesis of NHC–Au(I) complexes…………………………………………………S2

The synthesis of P–Au(I) complexes……………………………………………………S23

Typical procedure for intramolecular hydroamination reaction…………………………S33

References…………………………………………………………………………………S38

General remarks. Dichloromethane was freshly distilled from calcium hydride; THF and toluene were distilled from sodium (Na) under argon (Ar) atmosphere. Melting points were determined on a digital melting point apparatus and temperatures were uncorrected. ^{1}H NMR, ^{13}C NMR, ^{19}F NMR and ^{31}P NMR spectra were recorded on a Bruker AM-400 spectrophotometers. Infrared spectra were recorded on a Perkin-Elmer PE-983 spectrometer with absorption in cm^{-1}. Flash column chromatography was performed using 300–400 mesh silica gel. For thin-layer chromatography (TLC), silica gel plates (Huanghai GF_{254}) were used. Elementary analysis was taken on a Carlo-Erba 1106 analyzer. Mass spectra were recorded by ESI, and HRMS were measured on a HP-5989 instrument.

Synthesis of NHC–Au(I) complexes

Synthesis of (*S*)-2'-(3,5-bis(trifluoromethyl)phenyl)-[1,1'-binaphthalen]-2-ol (6)

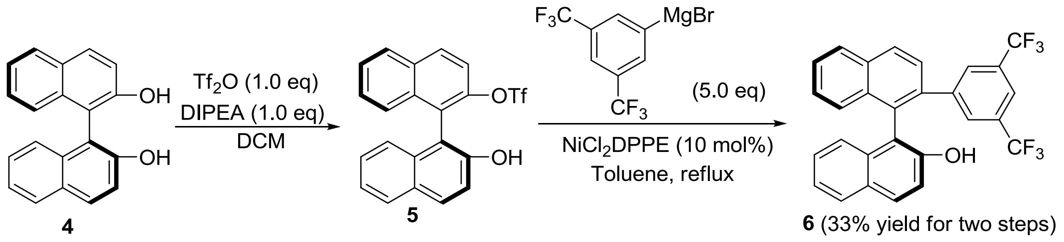

Tf_2O (1.18 mL, 10 mmol) was added dropwise into a solution of (S)-binaphthol (2.86 g, 10 mmol) and iPr_2NEt (1.74 mL, 10 mmol) in CH_2Cl_2 (50 mL) at 0 °C under argon. The reaction mixture was stirred for 1 h at this temperature and then quenched with saturated $NaHCO_3$. Extractive workup was performed with CH_2Cl_2, and the combined extracts were washed with brine and dried over anhydrous Na_2SO_4. The solvents was evaporation, then dried under vacuum, the crude product and $NiCl_2$(dppe) (530 mg, 1 mmol) was dissolved into THF (40 mL) under argon. To this solution was added dropwise a 1 M THF solution of 3,5-bis(trifluoromethyl)phenyl magnesium bromide (60 mL, 60 mmol) at 0 °C, and stirring was continued under reflux for overnight. The reaction was quenched with saturated NH_4Cl carefully and extractive workup was conducted with EA. The organic extracts were washed with brine and dried over anhydrous Na_2SO_4. Removal of solvents and purification of the residual oil by column

chromatography on silica gel afforded (*S*)-**6** (1.59 g, 3.3 mmol, 33% yield) as yellow powder. This is a known compound.[1] ^{1}H NMR (400 MHz, $CDCl_3$, TMS): δ 8.10 (d, *J* = 8.4 Hz, 1H, ArH), 8.01 (d, *J* = 8.0 Hz, 1H, ArH), 7.77 (t, *J* = 9.2 Hz, 2H, ArH), 7.66 (d, *J* = 8.4 Hz, 1H, ArH), 7.59-7.53 (m, 4H, ArH), 7.45-7.37 (m, 2H, ArH), 7.28-7.19 (m, 2H, ArH), 7.12 (d, *J* = 9.2 Hz, 1H, ArH), 6.98 (d, *J* = 8.4 Hz, 1H, ArH), 4.81 (br, 1H, OH); ^{19}F NMR (376 MHz, $CDCl_3$, $CFCl_3$): δ -63.107.

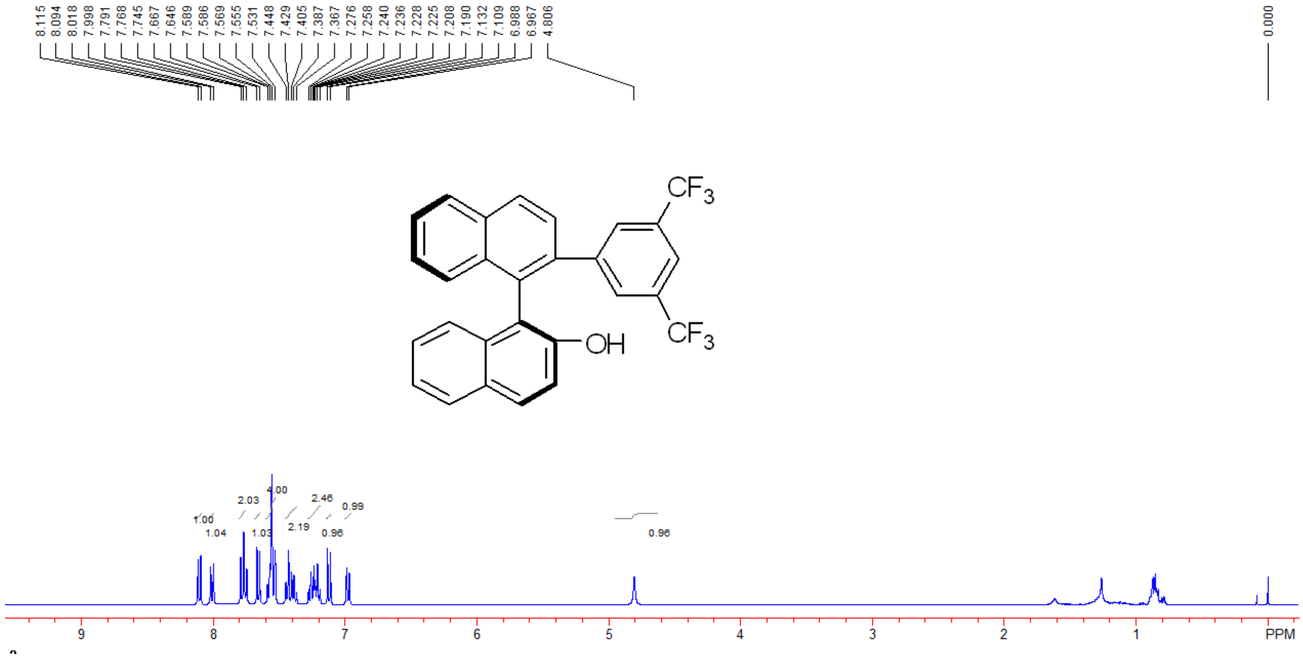

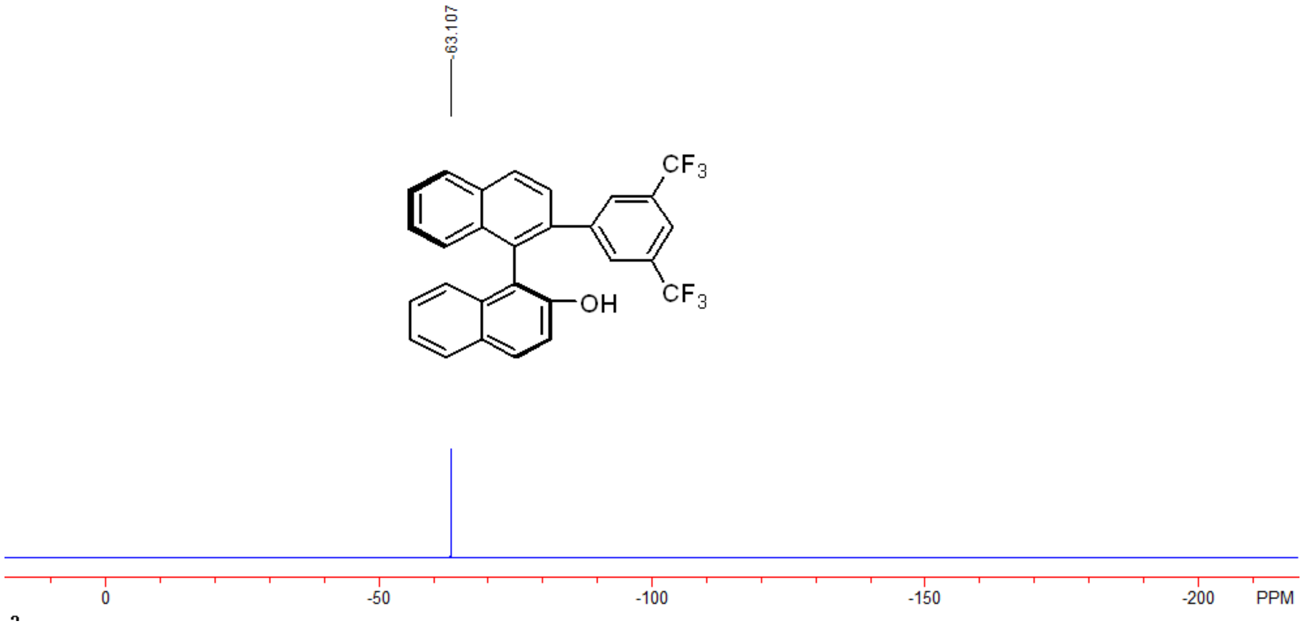

Synthesis of (*S*)-2'-(3,5-bis(trifluoromethyl)phenyl)-[1,1'-binaphthalen]-2-yl trifluoromethanesulfonate (7)

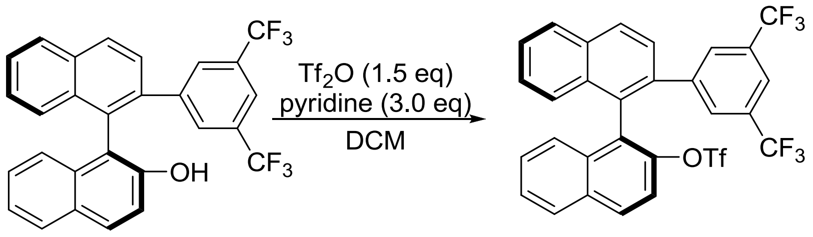

6 **7** (99% yield)

2-hydroxy-1,1'-binaphthyl derivative **6** (1.45 g, 3.0 mmol) was dissolved into dry CH_2Cl_2 (12 mL) at 0 °C. Then pyridine (0.7 mL, 9.0 mmol) was added, followed by slow syringe addition of triflic anhydride (1.27 g, 4.5 mmol). The reaction mixture was returned to room temperature slowly. After complete consumption of starting material as monitored by TLC, the solution volume was doubled with CH_2Cl_2. The mixture was washed with 1.0 N HCl, followed by water and saturated $NaHCO_3$. The organic layer was separated and dried over anhydrous sodium sulfate. The solvent was evaporated and dried under vacuum to yield the solid triflate **7** (1.83 g, 99% yield). This is a known compound.[1] ^{1}H NMR (400 MHz, $CDCl_3$, TMS): δ 8.16 (d, J = 8.4 Hz, 1H, ArH), 8.02 (d, J = 8.0 Hz, 1H, ArH), 7.97-7.94 (m, 2H, ArH), 7.67 (d, J = 8.8 Hz, 1H, ArH), 7.60-7.55 (m, 3H, ArH), 7.48-7.44 (m, 3H, ArH), 7.42-7.36 (m, 2H, ArH), 7.30-7.26 (m, 2H, ArH); ^{19}F NMR (376 MHz, $CDCl_3$, $CFCl_3$): δ -63.325, -75.020.

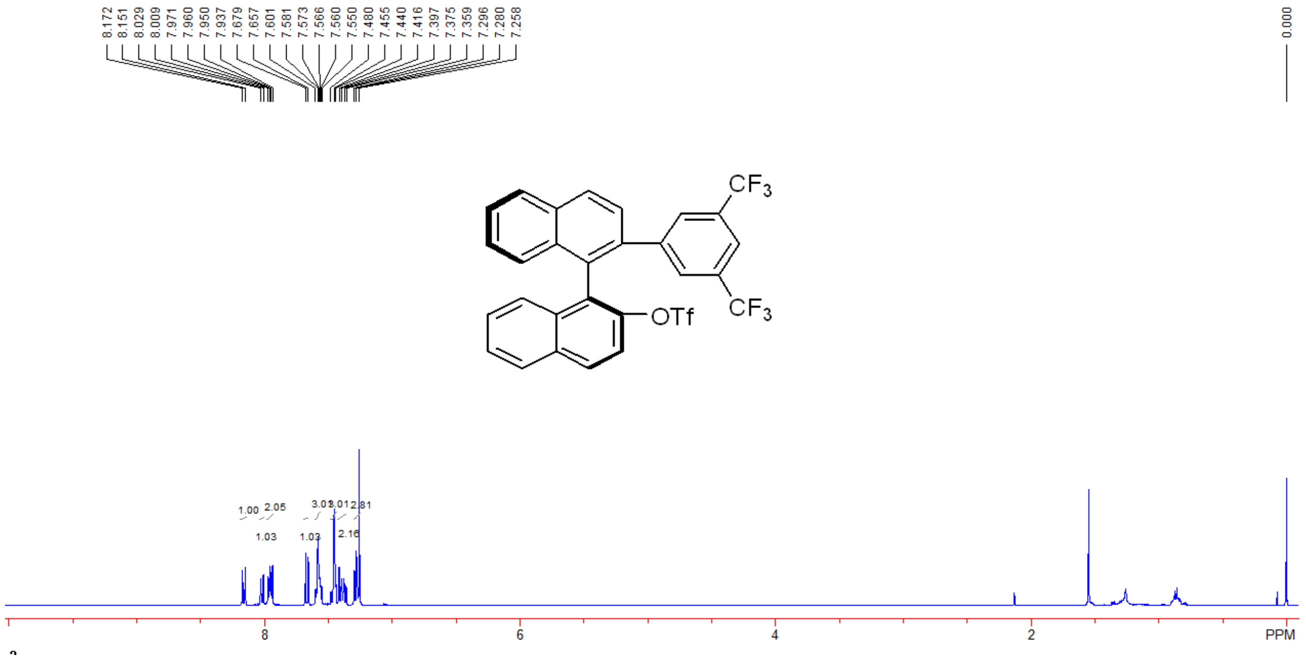

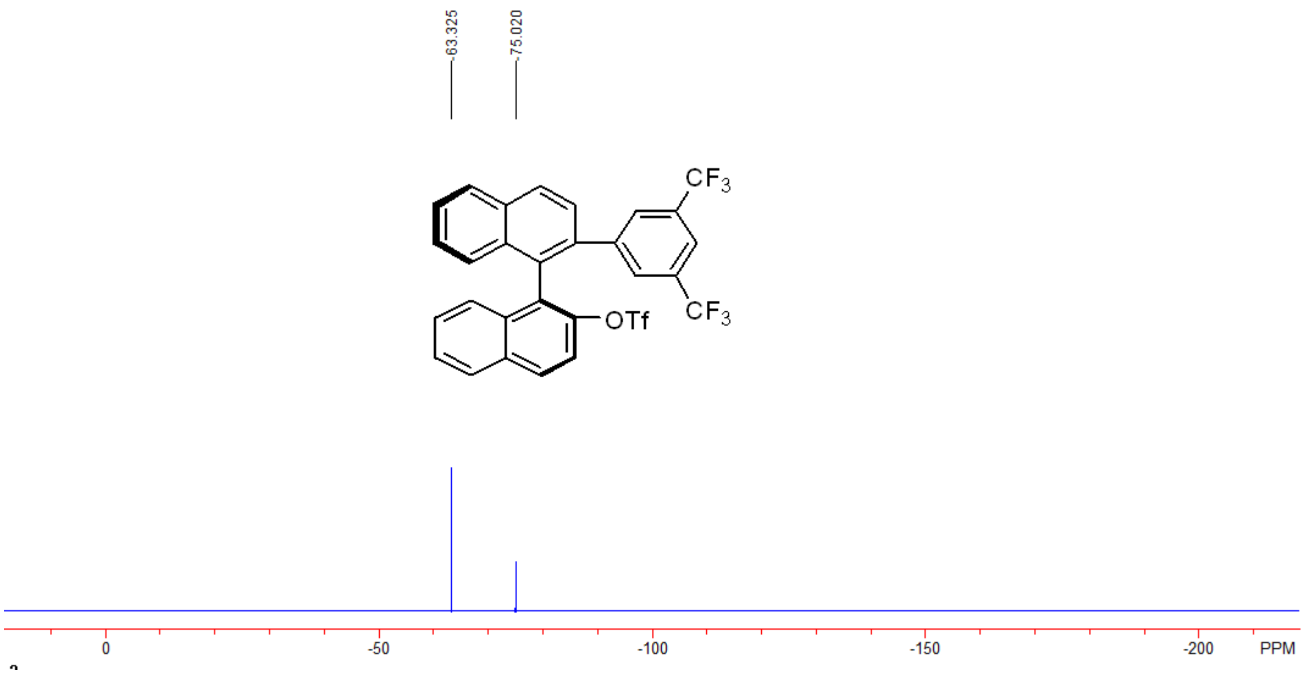

Synthesis of (*S*)-*N*-benzyl-2'-(3,5-bis(trifluoromethyl)phenyl)-[1,1'-binaphthalen]-2-amine (8)

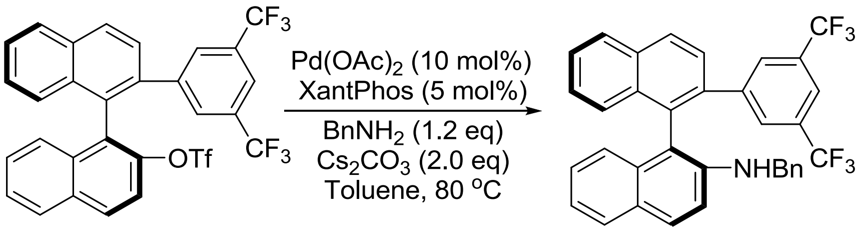

7 **8** (57% yield)

$Pd(OAc)_2$ (34 mg, 0.15 mmol) and xantphos [4,5-bis(diphenylphosphino)-9,9-dimethylxanthene; 174 mg, 0.3 mmol], freshly prepared triflate (*S*)-**7** (1.83 g, 3 mmol) and Cs_2CO_3 (1.97 g, 6 mmol), were added to freshly distilled dry toluene (9 mL) in a sealable reaction vessel under argon, and the reaction mixture was stirred at rt. After 5 min, benzylamine (0.4 mL, 3.6 mmol) was added under argon. The mixture was stirred for 3 d at 80 °C. The reaction mixture was then cooled to room temperature, diluted with EA, and filtered through celite. The organic extracts were washed with brine and dried over anhydrous Na_2SO_4. The solvent was evaporated to obtain crude product. The pure product (*S*)-**8** (976 mg, 57% yield) was purified by flash chromatography on silica gel using CH_2Cl_2/hexanes as the eluent. This is a known compound.[1] ^{1}H NMR (400 MHz, $CDCl_3$, TMS): δ 8.09 (d, J = 8.4 Hz, 1H, ArH), 8.00 (d, J =

8.4 Hz, 1H, ArH), 7.71-7.65 (m, 5H, ArH), 7.58-7.54 (m, 2H, ArH), 7.47 (d, J = 8.0 Hz, 1H, ArH), 7.40-7.35 (m, 1H, ArH), 7.24-7.10 (m, 5H, ArH), 7.03-7.00 (m, 2H, ArH), 6.95 (d, J = 9.2 Hz, 1H, ArH), 6.85-6.82 (m, 1H, ArH), 4.28 (s, 2H, CH_2), 3.87 (br, 1H, NH); ^{19}F NMR (376 MHz, $CDCl_3$, $CFCl_3$): δ -63.020; ^{13}C NMR (100 MHz, $CDCl_3$): δ 143.7, 143.1, 139.3, 137.6, 134.0, 133.9, 132.8, 132.7, 130.5 (d, ^{2}J(C,F) = 33 Hz), 129.8, 129.3, 129.03, 129.02, 128.5, 128.3, 128.1, 127.4, 127.3, 127.1, 127.03, 127.02, 126.8, 126.62, 126.58, 123.4, 123.1 (d, ^{1}J(C,F) = 271 Hz), 121.9, 120.3 (septet, ^{3}J(C,F) = 3.7 Hz), 114.1, 113.5, 47.8.

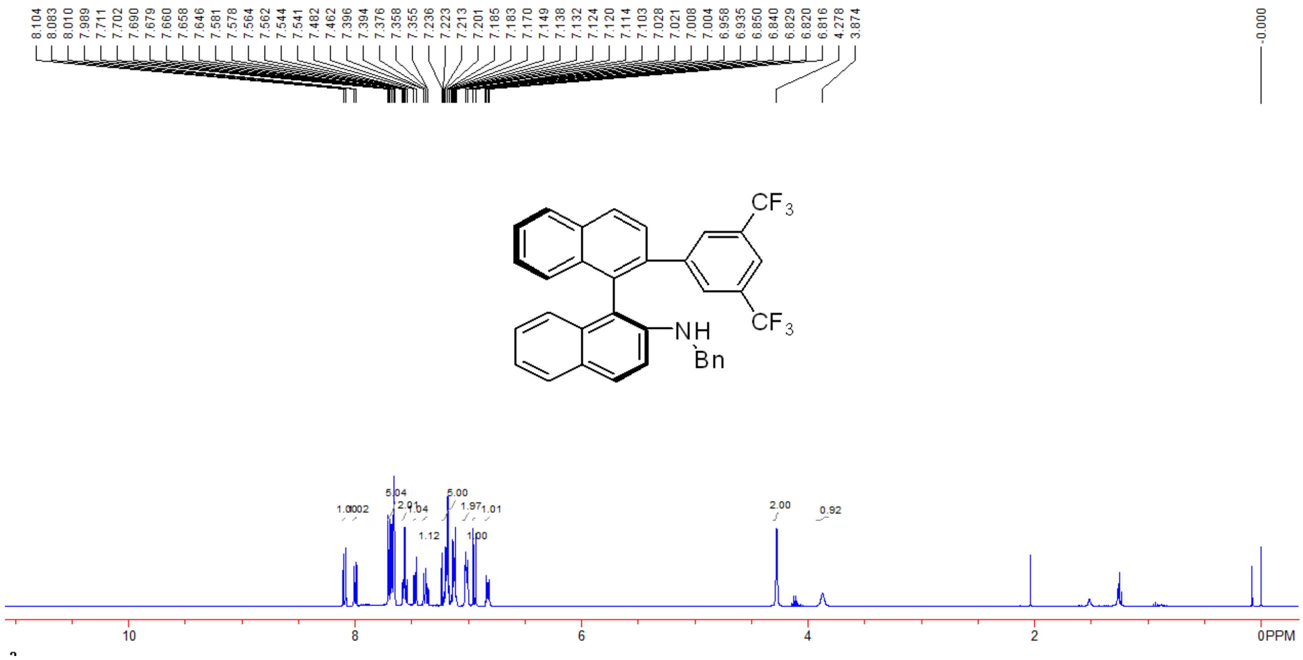

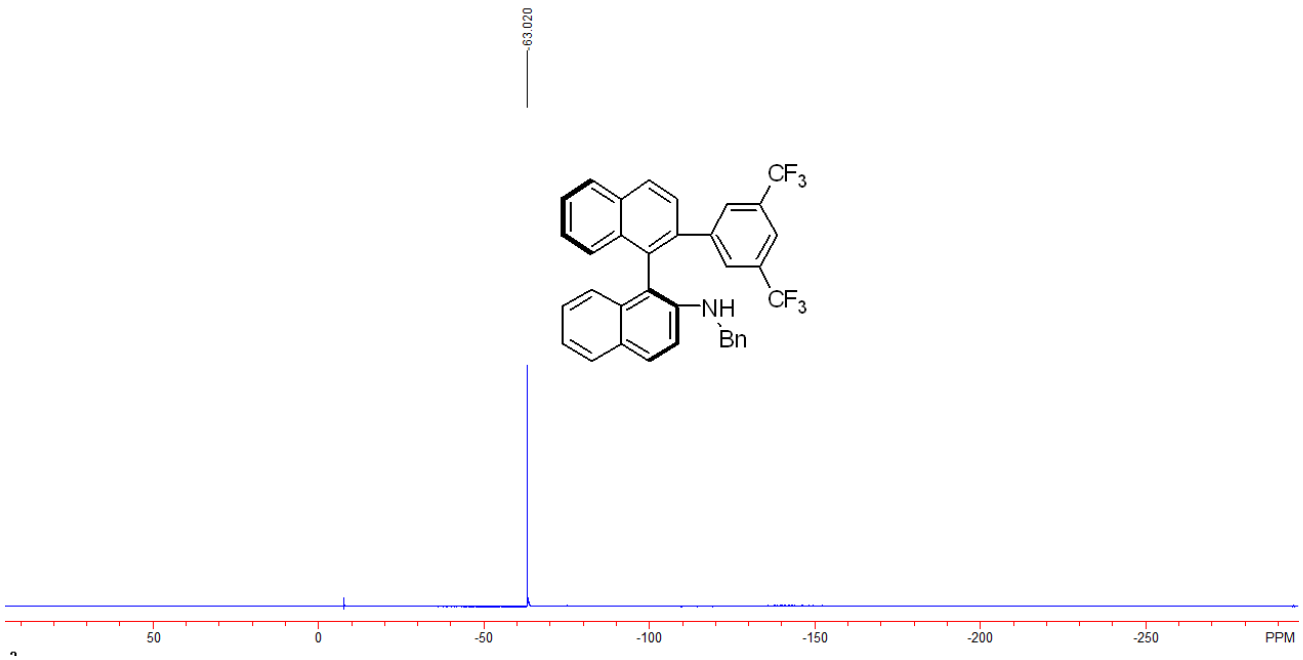

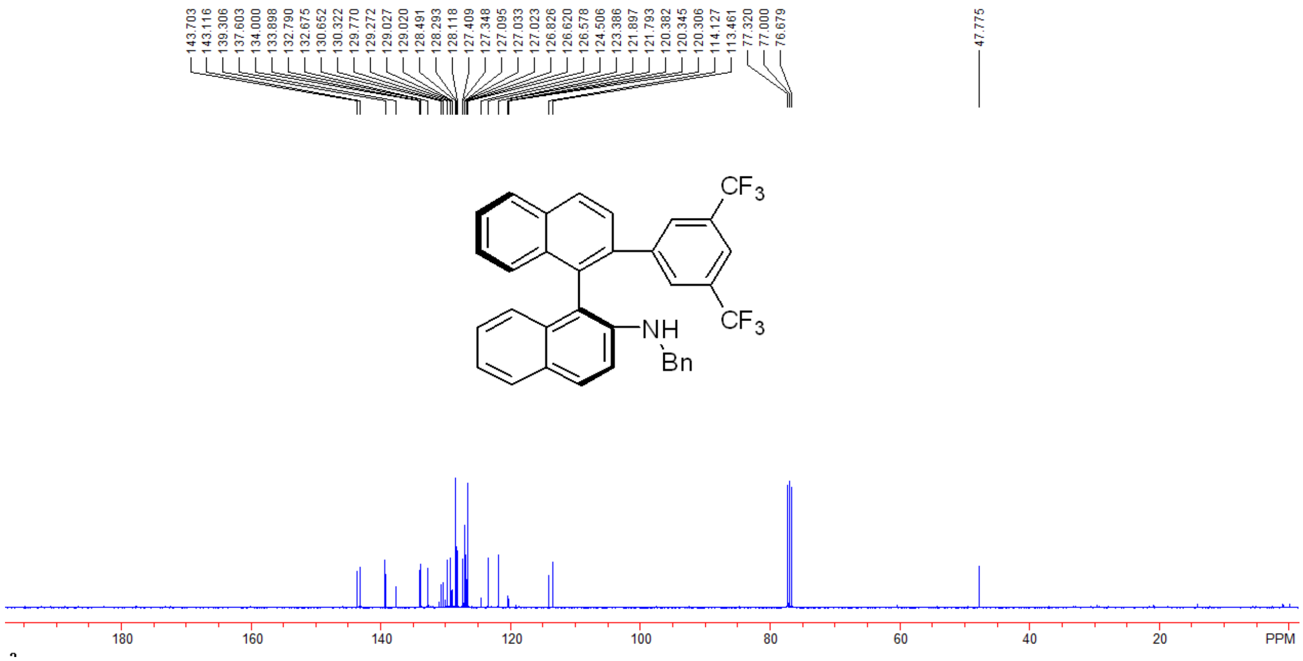

Synthesis of (*S*)-2'-(3,5-bis(trifluoromethyl)phenyl)-[1,1'-binaphthalen]-2-amine (9)

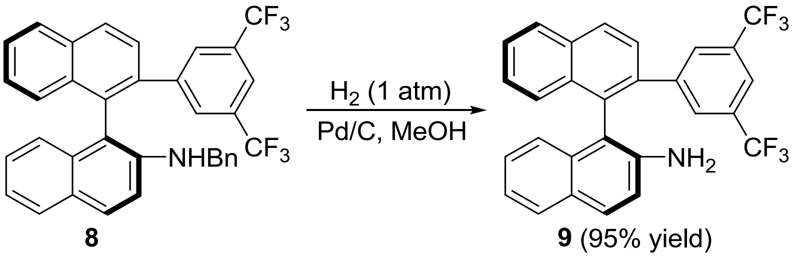

A solution of 2-benzylamino-1,1'-binaphthyl derivative (*S*)-**8** (856 mg, 1.5 mmol) was dissolved in MeOH (12 mL) in an oven-dried two-neck flask equipped with a argon supply. Under argon, 5% Pd/C (86 mg) was added. The argon atmosphere was then removed under vacuum, and the apparatus was placed under 1 atm of H_2 (hydrogen balloon). The mixture was stirred for overnight under reflux, until complete consumption of starting material was indicated by TLC. The hydrogen atmosphere was then removed under vacuum, and the reaction mixture was flushed generously with nitrogen. The suspended Pd/C was removed by filtration through celite, and the solvent was evaporated to obtain the solid product (*S*)-**9** (685 mg, 95% yield). They are indefinitely stable if stored in an argon atmosphere. This is a known compound.[1] ^{1}H NMR (400 MHz, $CDCl_3$, TMS): δ 8.09 (d, *J* = 8.8 Hz, 1H, ArH), 8.00 (d, *J* = 8.0 Hz, 1H, ArH), 7.70-7.65 (m, 5H, ArH), 7.55 (t, *J* = 8.0 Hz, 1H, ArH), 7.52 (s, 1H, ArH), 7.47 (d, *J* =

8.4 Hz, 1H, ArH), 7.39-7.35 (m, 1H, ArH), 7.15-7.10 (m, 2H, ArH), 6.94 (d, J = 8.8 Hz, 1H, ArH), 6.84-6.82 (m, 1H, ArH), 3.58 (br, 2H, NH); ^{19}F NMR (376 MHz, $CDCl_3$, $CFCl_3$): δ -63.470; ^{13}C NMR (100 MHz, $CDCl_3$): δ 143.1, 142.4, 137.5, 133.83, 133.77, 132.7, 132.5, 130.4 (d, ^{2}J(C,F) = 33 Hz), 129.6, 129.1, 129.0, 128.3, 128.0, 127.7, 127.4, 127.3, 126.9, 126.7, 126.4, 123.5, 123.1 (d, ^{1}J(C,F) = 271 Hz), 122.2, 120.3, 117.4, 114.4.

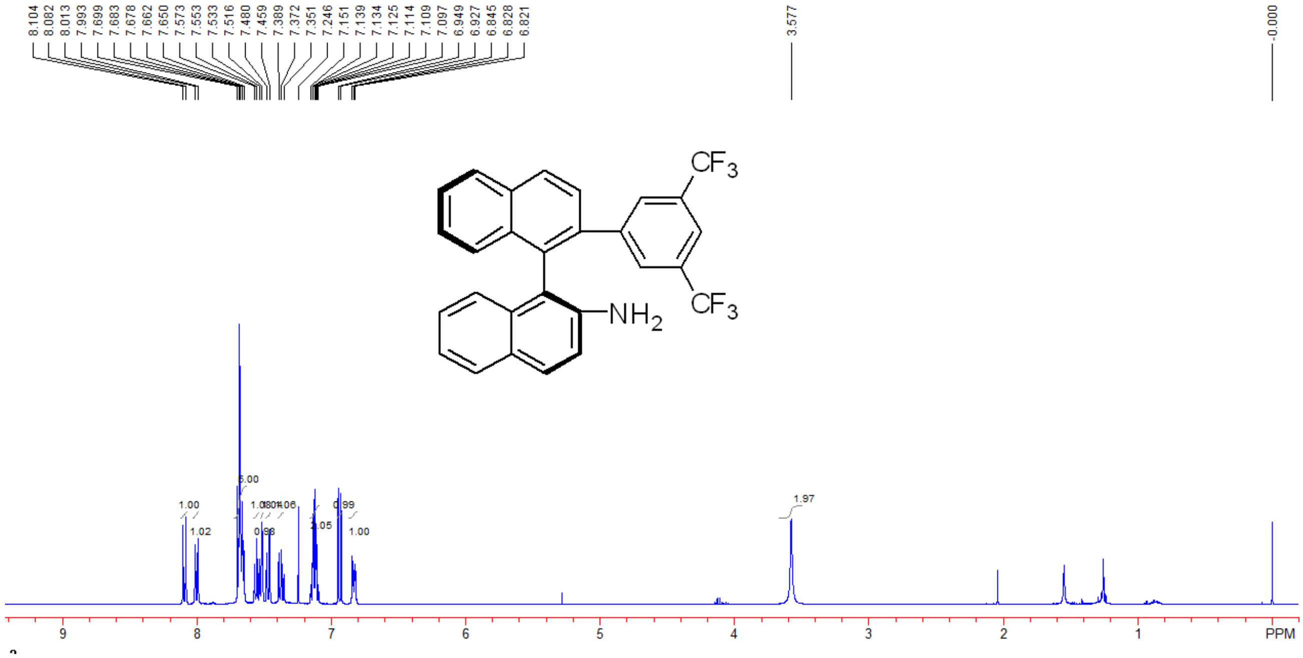

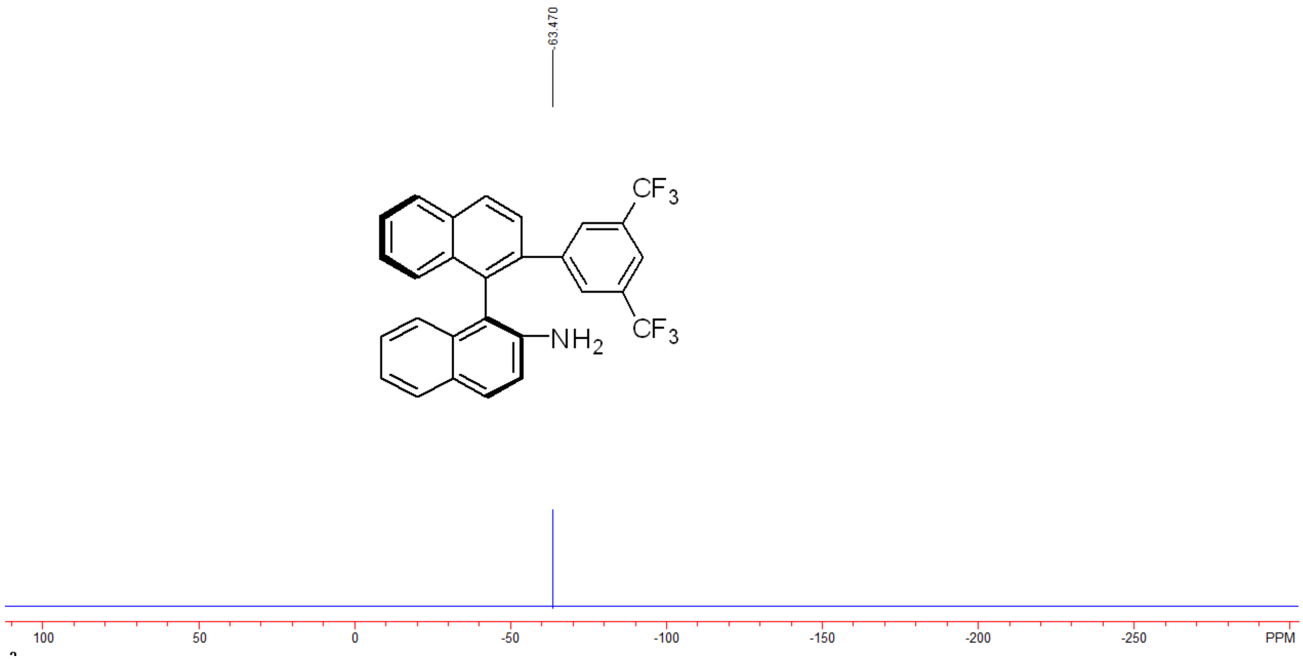

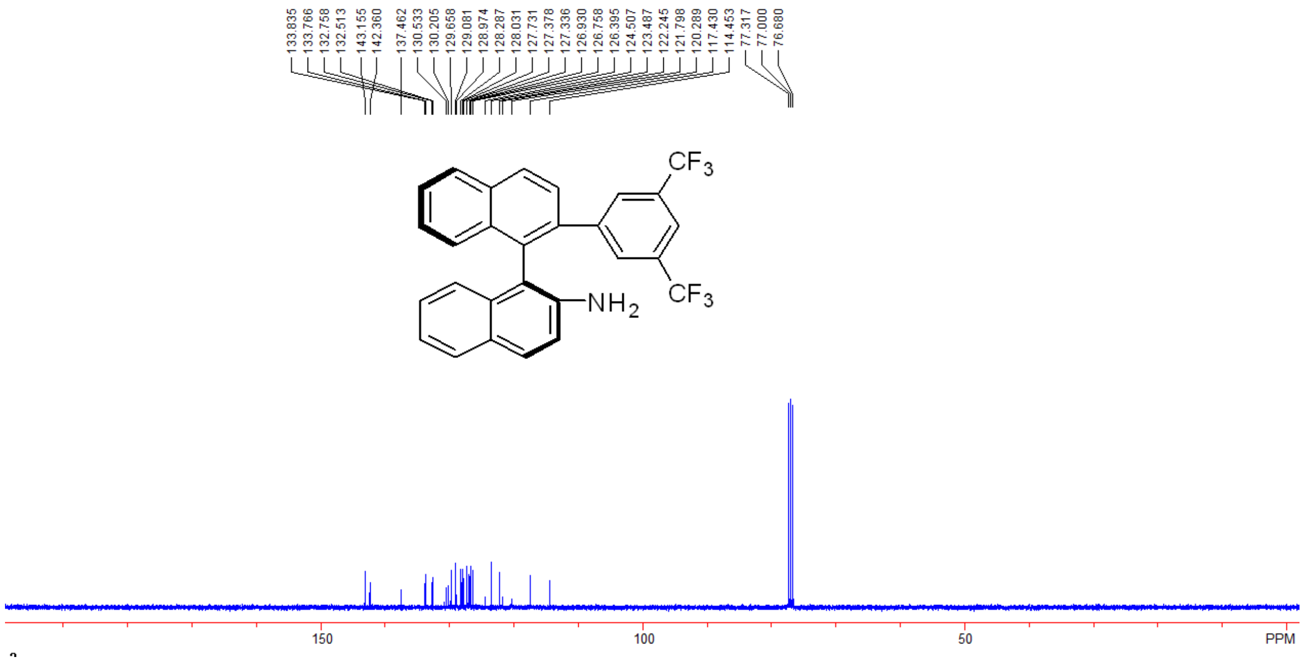

Synthesis of (*S*)-2'-(3,5-bis(trifluoromethyl)phenyl)-*N*-(2-nitrophenyl)-[1,1'-binaphthalen]-2-amine (10)

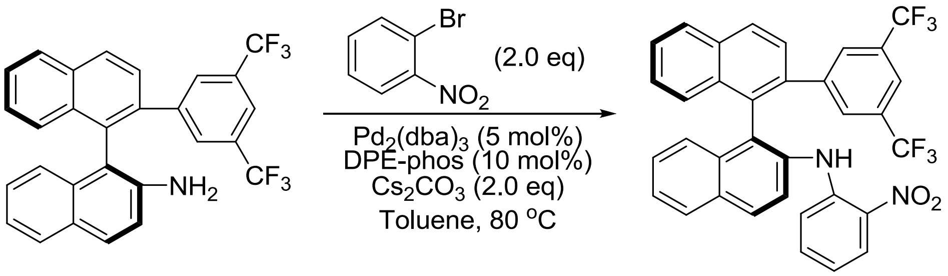

9 **10** (94% yield)

A mixture of 2-amino-1,1'-binaphthyl derivative (*S*)-**9** (673 mg, 1.4 mmol), 1-bromo-2-nitrobenzene (563 mg, 2.8 mmol), $Pd_2(dba)_3$ (64 mg, 0.07 mmol), DPE-phos (77 mg, 0.14 mmol) and Cs_2CO_3 (913 mg, 2.8 mmol) was dissolved into toluene (6.0 mL) under argon. The mixture was stirred at 80 °C for 3 d. The reaction mixture was then cooled to room temperature, diluted with EA, and filtered through celite. The organic extracts were washed with brine and dried over anhydrous Na_2SO_4. The solvent was evaporated to obtain crude product. The pure product (*S*)-**10** (792 mg, 94% yield) was purified by flash chromatography on silica gel using PE/EA as the eluent. This is a new compound. $[\alpha]^{20}_{D}$ 26.7 (*c* 0.25, CH_2Cl_2); ^1H NMR (400 MHz, $CDCl_3$, TMS): δ 8.83 (s, 1H, NH), 8.11 (d, *J* = 8.8 Hz, 1H, ArH), 8.02 (d,

J = 8.0 Hz, 1H, ArH), 7.98 (dd, J_1 = 8.8 Hz, J_2 = 1.6 Hz, 1H, ArH), 7.89-7.87 (m, 2H, ArH), 7.65 (d, J = 8.4 Hz, 1H, ArH), 7.58-7.54 (m, 1H, ArH), 7.51 (s, 1H, ArH), 7.49 (d, J = 9.2 Hz, 1H, ArH), 7.46-7.42 (m, 3H, ArH), 7.39-7.34 (m, 3H, ArH), 7.29 (d, J = 8.4 Hz, 1H, ArH), 7.19-7.15 (m, 1H, ArH), 6.76 (d, J = 8.8 Hz, 1H, ArH), 6.70-6.66 (m, 1H, ArH); ^{19}F NMR (376 MHz, $CDCl_3$, $CFCl_3$): δ -63.480; ^{13}C NMR (100 MHz, $CDCl_3$): δ 142.9, 140.8, 136.6, 135.3, 135.0, 134.6, 133.6, 133.5, 132.5, 131.8, 130.7, 130.6 (d, ^{2}J(C,F) = 33 Hz), 129.7, 129.3, 129.1, 128.6, 128.3, 127.6, 127.5, 127.10, 127.06, 127.0, 126.5, 126.0, 125.6, 125.4, 122.8 (d, ^{1}J(C,F) = 271 Hz), 121.3, 120.3 (septet, ^{3}J(C,F) = 3.7 Hz), 118.2, 115.0; IR (CH_2Cl_2): ν 2962, 1612, 1496, 1277, 1259, 1128, 1090, 1015, 797, 737, 707, 680 cm^{-1}; MS (m/z): HRMS (ESI) Calcd. for $C_{34}H_{21}F_6N_2O_2$ ($[M+H]^+$); 603.1502. Found: 603.1489.

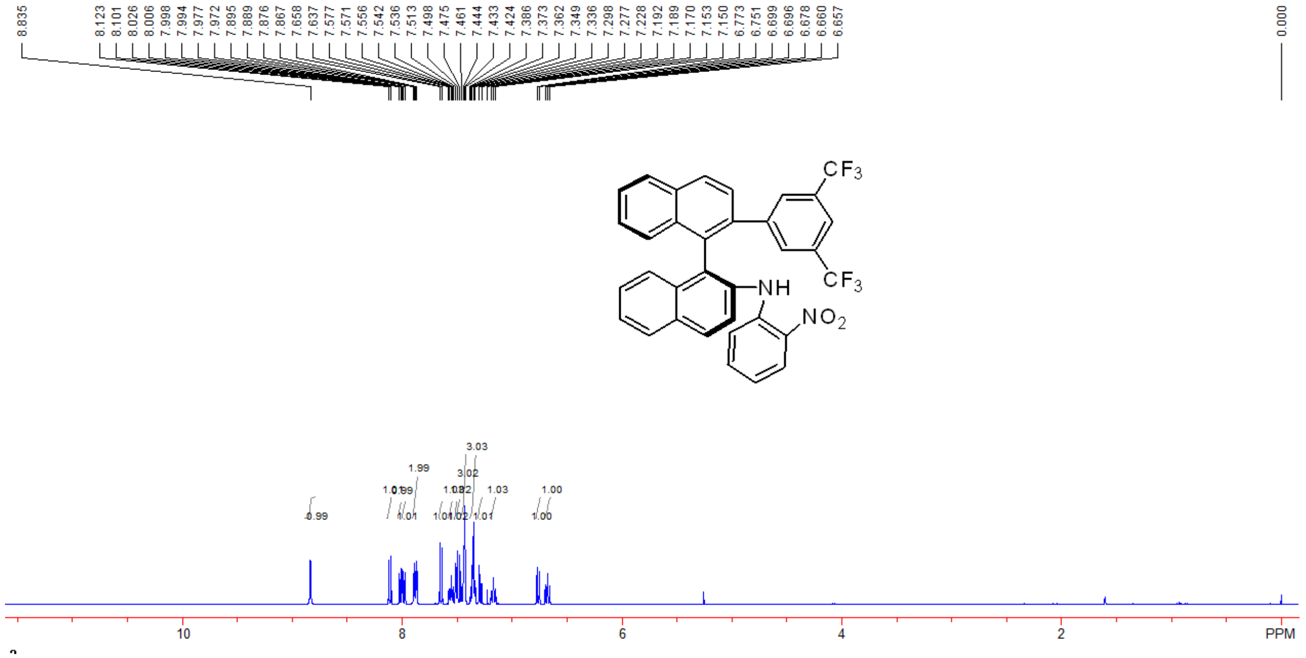

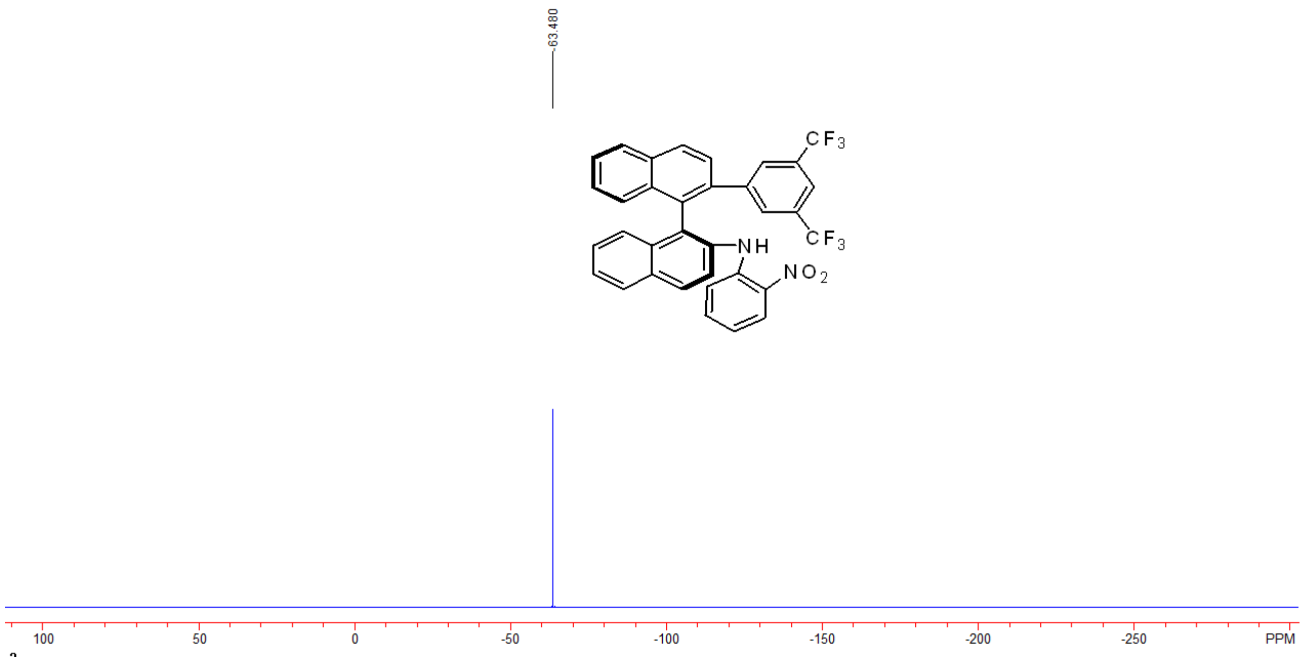

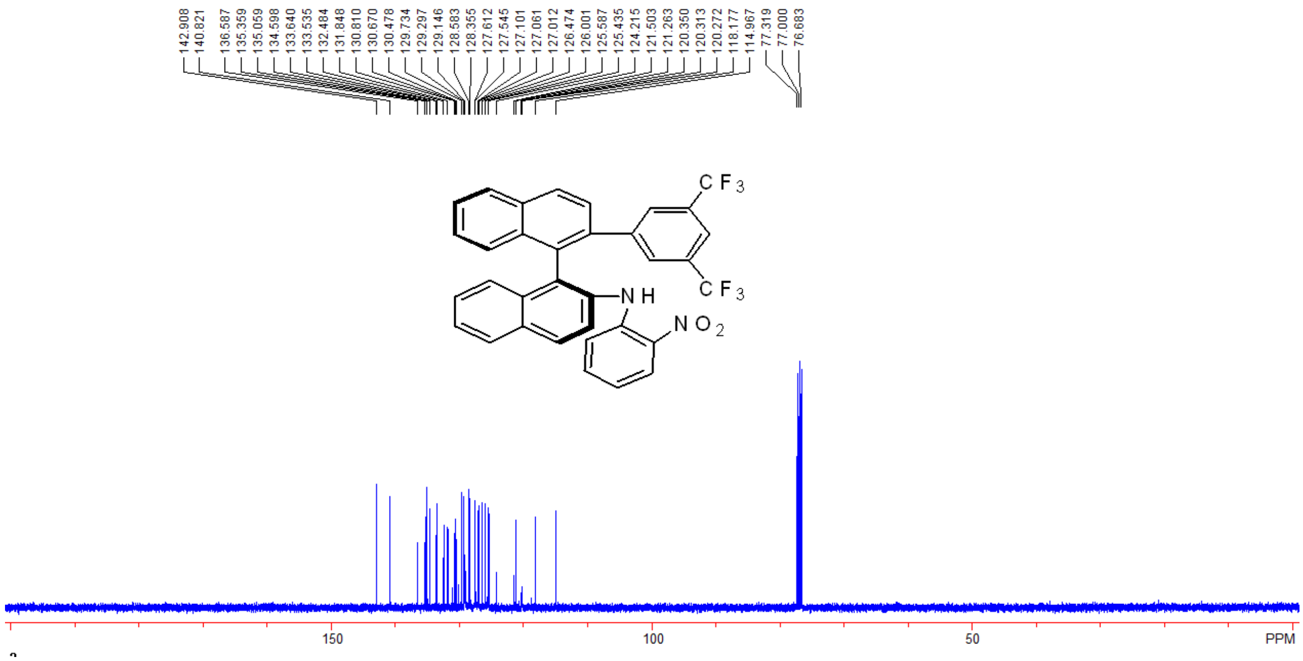

Synthesis of (*S*)-N^1-(2'-(3,5-bis(trifluoromethyl)phenyl)-[1,1'-binaphthalen]-2-yl)benzene-1,2-diamine (11)

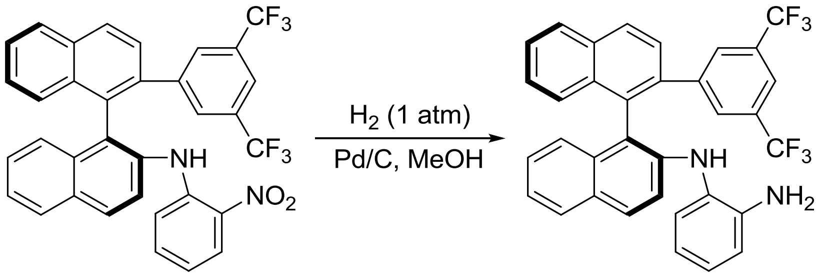

10 **11** (95% yield)

A mixture of 1,1'-binaphthyl derivative (*S*)-**10** (782 mg, 1.3 mol) and 10% Pd/C (78 mg) was dissolved into MeOH (6 mL) under argon. The argon atmosphere was then removed under vacuum, and the apparatus was placed under 1 atm of H_2 (hydrogen balloon). The mixture was stirred for overnight under reflux, until complete consumption of starting material was indicated by TLC. The hydrogen atmosphere was then removed under vacuum. The suspended Pd/C was removed by filtration through celite. The solvent was evaporated to obtain crude product (*S*)-**11** (706 mg, 95% yield). This is a new compound. ^{1}H NMR (400 MHz, $CDCl_3$, TMS): δ 8.11 (d, *J* = 8.0 Hz, 1H, ArH), 8.01 (d, *J* = 8.0 Hz, 1H, ArH), 7.73-7.66 (m, 5H, ArH), 7.59-7.54 (m, 3H, ArH), 7.42-7.38 (m, 1H, ArH), 7.22-7.18 (m, 2H, ArH), 7.01-6.97 (m, 1H, ArH), 6.96-6.91 (m, 1H, ArH), 6.89 (d, *J* = 9.2 Hz, 1H, ArH), 6.65-6.58 (m, 3H, ArH), 4.71 (s, 1H, NH), 3.42 (br, 2H, NH_2); ^{19}F NMR (376 MHz, $CDCl_3$, $CFCl_3$): δ -63.382; ^{13}C NMR (100 MHz, $CDCl_3$): δ 143.1, 142.7, 141.7, 137.6, 134.3, 133.9, 132.8, 132.4, 130.6 (d, ^2J(C,F) = 33 Hz), 129.6, 129.5, 129.1, 128.5, 128.2, 128.0, 127.5, 127.4, 127.3, 127.1, 127.0, 126.5, 126.2, 126.1, 123.8, 123.0 (d, ^1J(C,F) = 271 Hz), 122.6, 121.7, 120.4 (septet, ^3J(C,F) = 3.6 Hz), 118.9, 115.73, 115.71, 115.69. IR (CH_2Cl_2): ν 3482, 3381, 3057, 2927, 1620, 1385, 1277, 1178, 1129, 813, 739, 708, 681 cm^{-1}. MS (m/z): HRMS (ESI) Calcd. for $C_{34}H_{22}F_6N_2$ ([M+H]$^+$); 572.1687. Found: 572.1690.

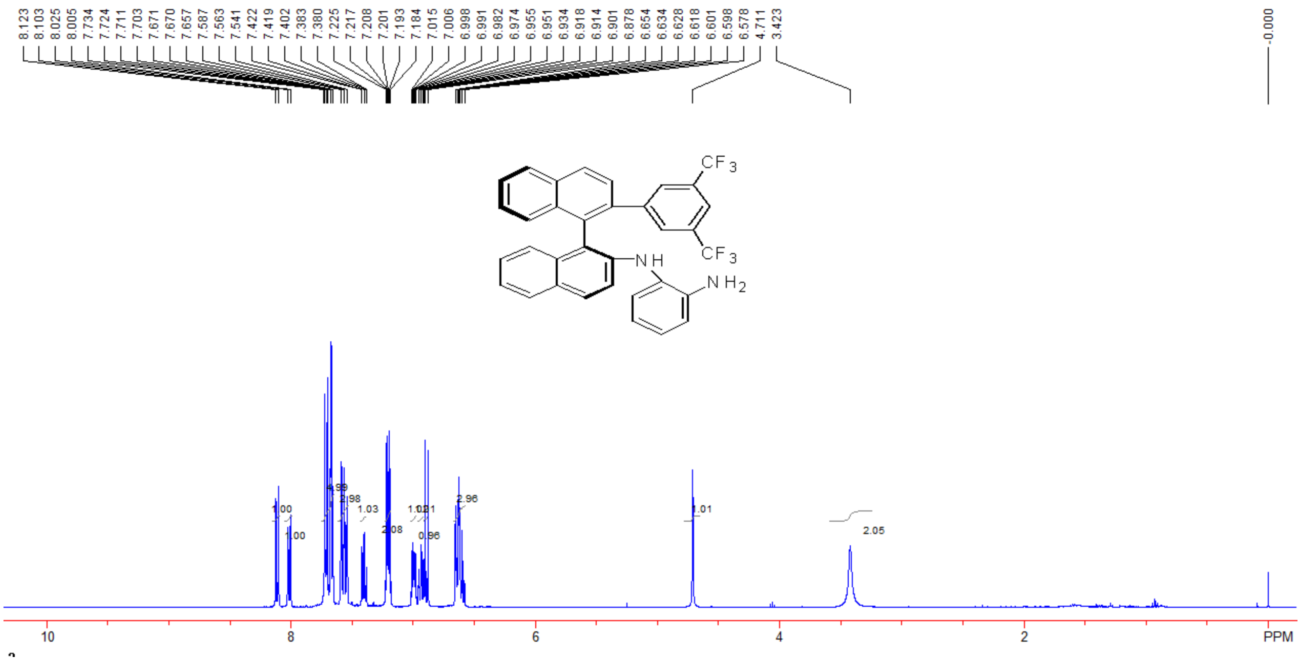
CF3
NH
NH2
PPM

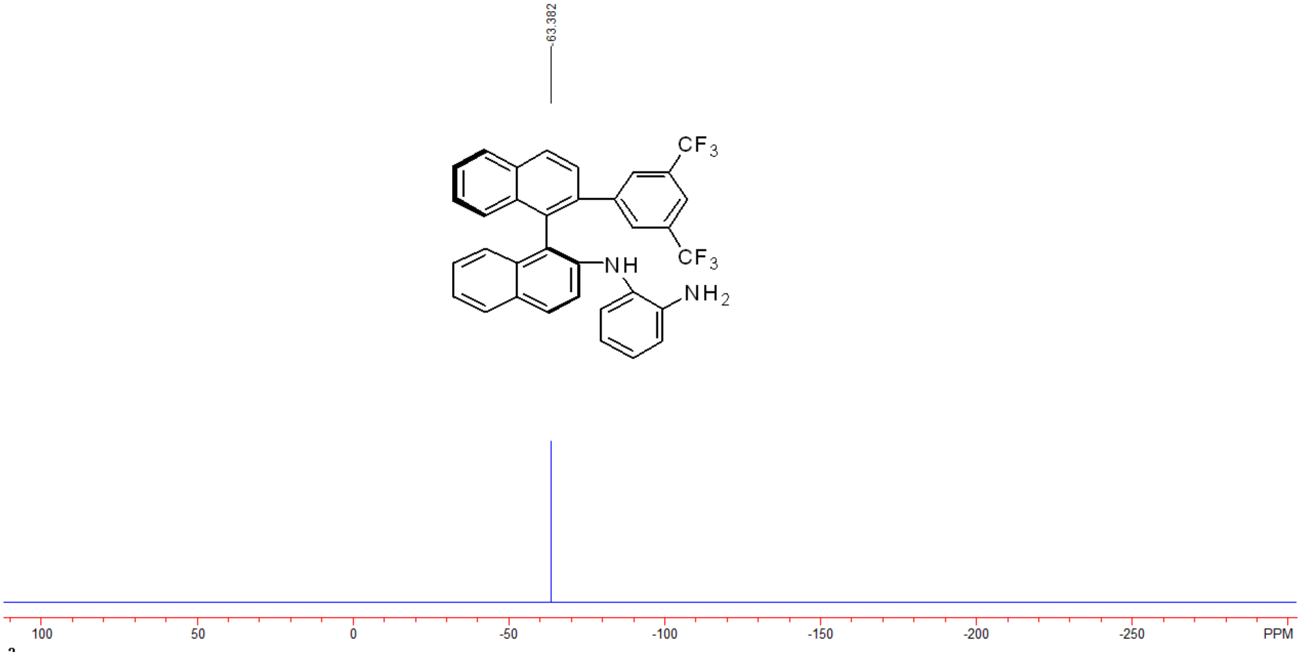
-63.382
CF3
NH
NH2
PPM

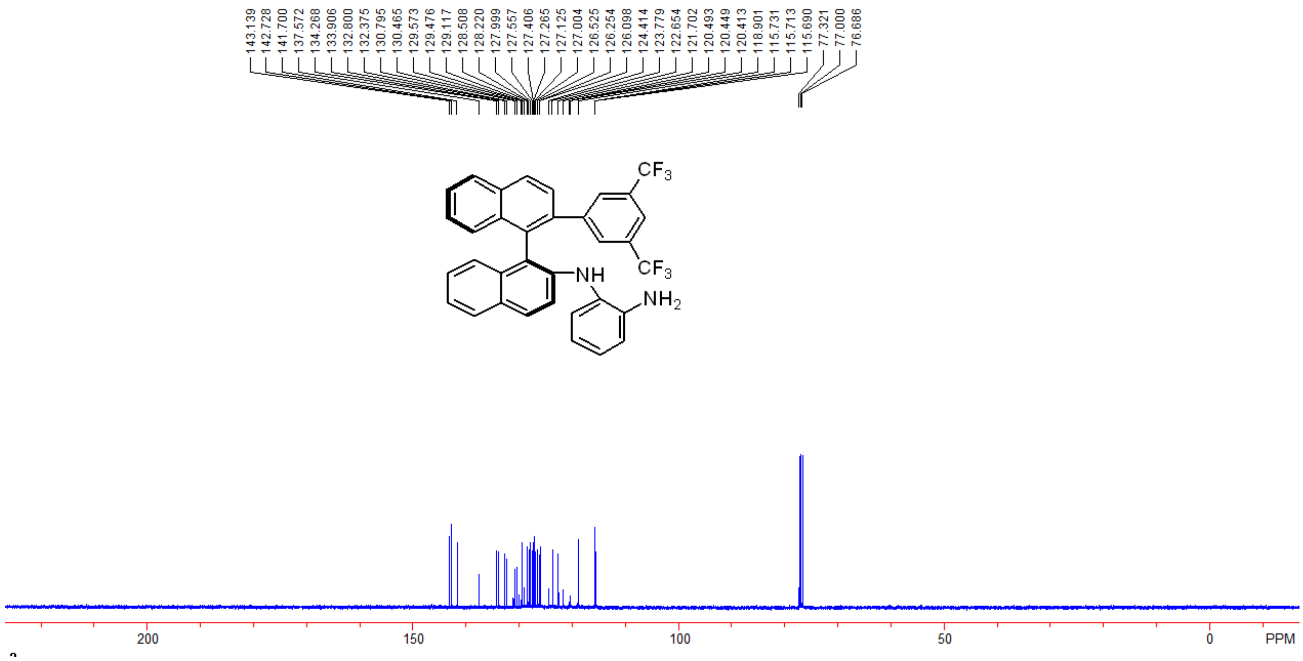

Synthesis of (*S*)-1-(2'-(3,5-bis(trifluoromethyl)phenyl)-[1,1'-binaphthalen]-2-yl)-1*H*-benzo[d]imidazole (12)

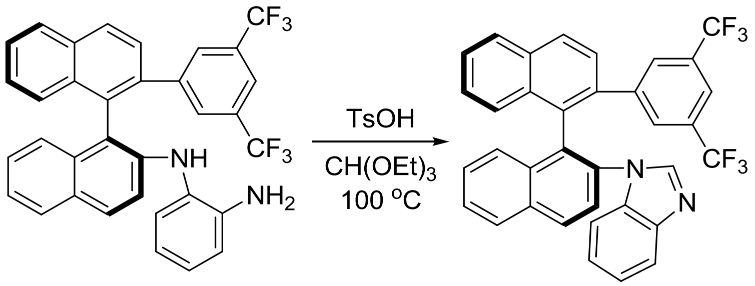

11 **12** (89% yield)

To a solution of 1,1'-binaphthyl derivative (*S*)-**11** (686 mg, 1.2 mmol) in $HC(OEt)_3$ (11 mL) under argon a catalytic amount of TsOH was added. The mixture was stirred for overnight at 100 ^{o}C. Until complete consumption of starting material was indicated by TLC, the solvent was evaporated to obtain crude product. The (*S*)-**12** (621 mg, 89% yield) was purified by flash chromatography on silica gel using PE/EA as the eluent. This is a new compound. $[\alpha]^{20}_{D}$ -42.8 (*c* 0.05, CH_2Cl_2); ^{1}H NMR (400 MHz, $CDCl_3$, TMS): δ 8.10 (t, *J* = 8.4 Hz, 2H, ArH), 8.02 (d, *J* = 8.4 Hz, 2H, ArH), 7,67-7.59 (m, 3H, ArH), 7.53-7.44 (m, 6H, ArH), 7.38 (d, *J* = 8.4 Hz, 1H, ArH), 7.19-7.15 (m, 1H, ArH), 6.97-6.93 (m, 2H, ArH), 6.80 (s, 2H, ArH), 6.32 (d, *J* = 8.0 Hz, 1H, ArH); ^{19}F NMR (376 MHz, $CDCl_3$, $CFCl_3$): δ -63.128;

^{13}C NMR (100 MHz, $CDCl_3$): δ 142.5, 142.0, 141.9, 135.8, 135.0, 133.9, 133.2, 133.1, 132.4, 132.3, 131.2, 130.76, 130.71 (d, ^{2}J(C,F) = 33 Hz), 130.3, 130.0, 128.8, 128.7, 128.4, 128.3, 128.0, 127.3, 127.1, 127.0, 126.8, 126.5, 123.7, 123.6, 122.65, 122.62 (d, ^{1}J(C,F) = 271 Hz), 120.4, 120.0, 108.6. IR (CH_2Cl_2): ν 2917, 2849, 1490, 1455, 1386, 1278, 1264, 1183, 1135, 820, 799, 737, 707 cm^{-1}; MS (m/z): HRMS (ESI) Calcd. for $C_{35}H_{21}F_6N_2$ ([M+H]$^+$); 583.1603. Found: 583.1600.

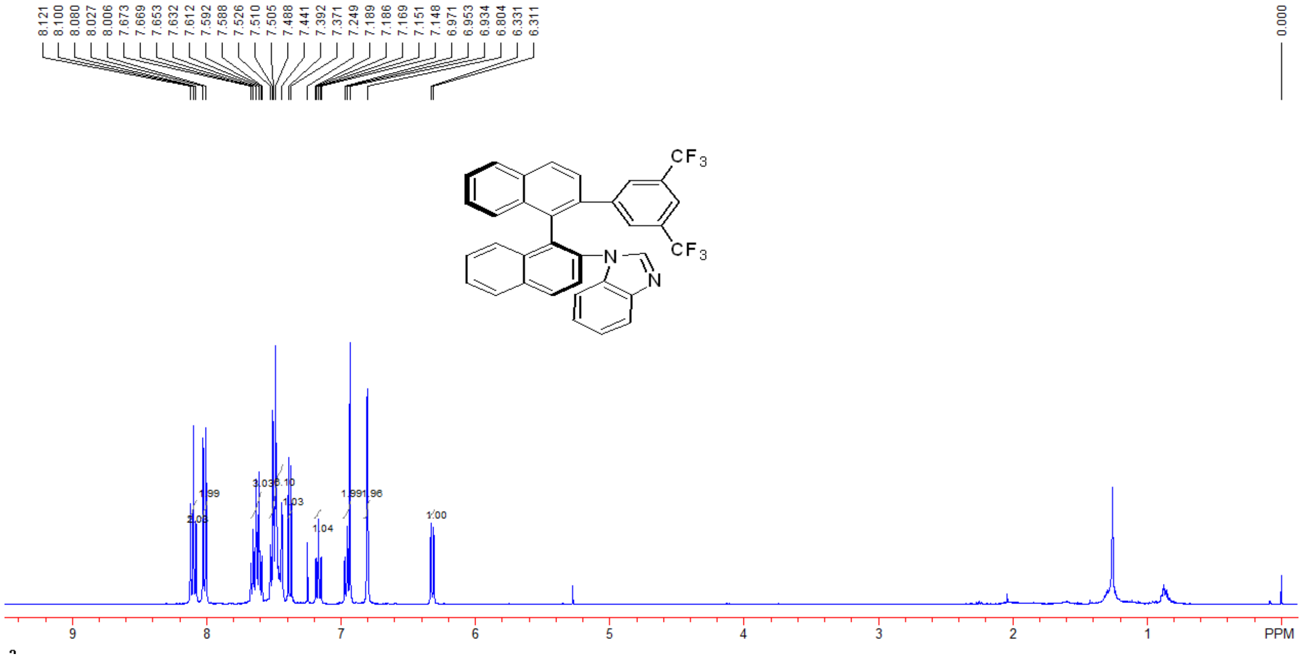

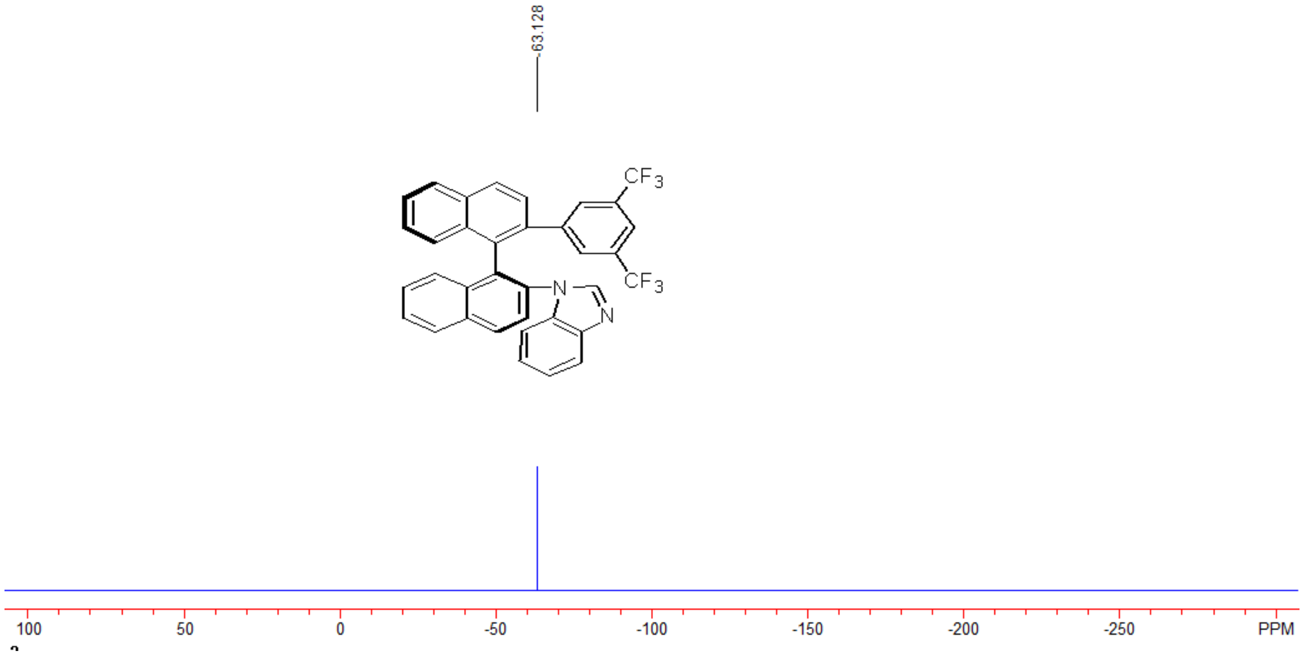

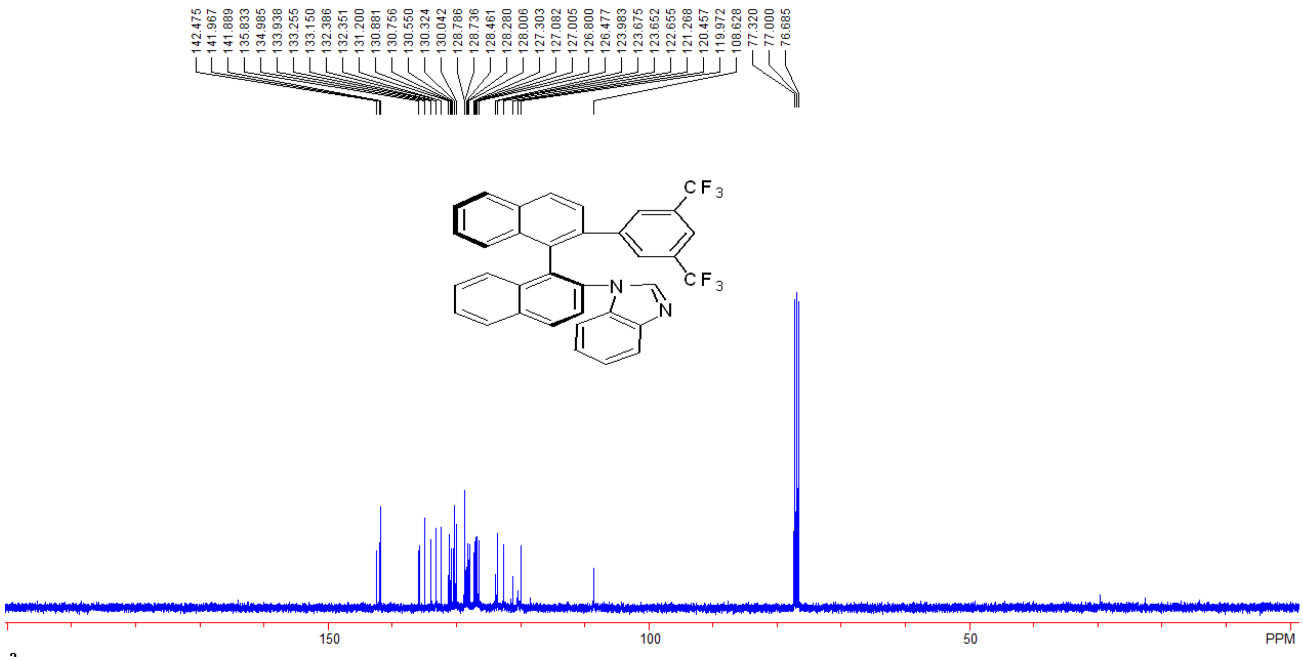

Synthesis of compound (*S*)-13

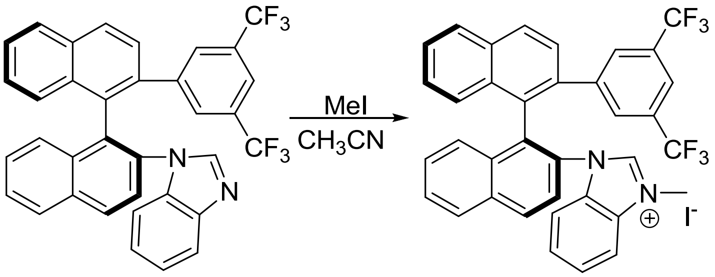

12 **13** (quantitative yield)

The compound (*S*)-**12** (291 mg, 0.5 mmol) and CH_3I (0.1 mL, 2.0 mmol) in CH_3CN (2.0 mL) were stirred under reflux for 5 h. After cooling to room temperature, volatiles were removed under reduced pressure and the obtained solid compound (*S*)-**13** (362 mg, quantitative yield) was used for the next reaction without further purification.

Synthesis of compound (*S*)-14

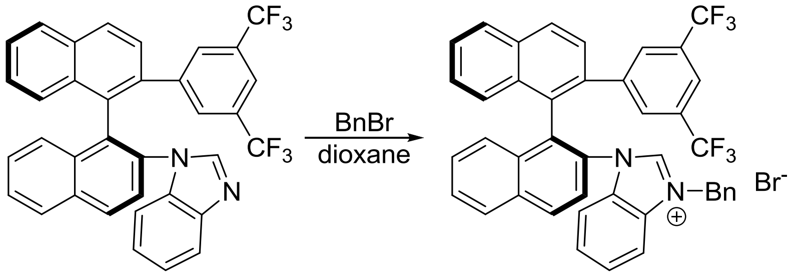

12 **14** (quantitative yield)

The compound (*S*)-**12** (291 mg, 0.5 mmol) and BnBr (0.1 mL, 1.0 mmol) in dioxane (2.0 mL) were stirred under reflux for 5 h. After cooling to room temperature, volatiles were removed under reduced pressure and the obtained solid compound (*S*)-**14** (376 mg, quantitative yield) was used for the next reaction without further purification.

Synthesis of gold-complexes (*S*)-15a and (*S*)-15b

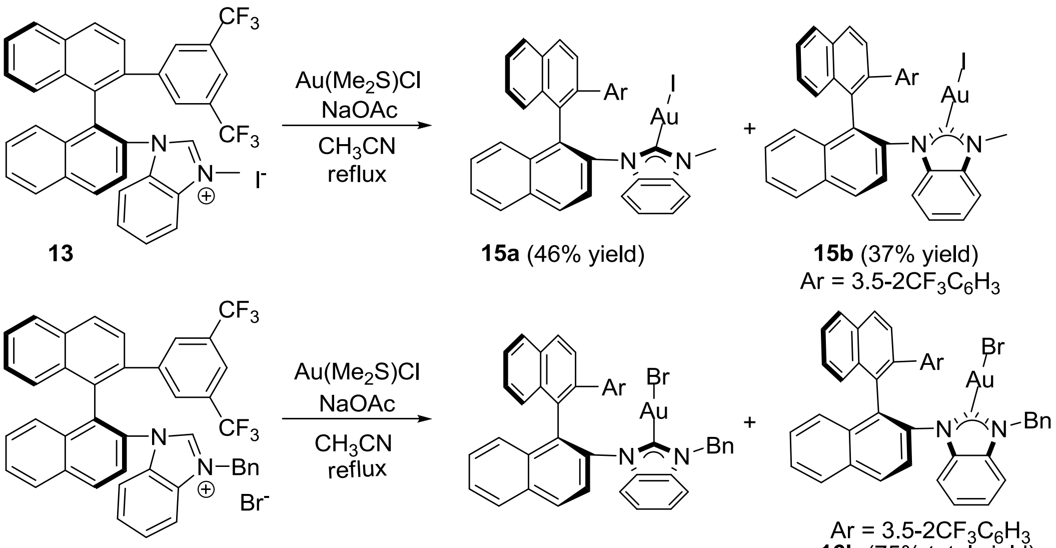

13 **15a** (46% yield) **15b** (37% yield)
Ar = 3.5-2CF$_3$C$_6$H$_3$

14 **16a** Ar = 3.5-2CF$_3$C$_6$H$_3$
16b (75% total yield)
The ratio is 2:1

The compound (*S*)-**13** (145 mg, 0.2 mmol) and AuCl·S(Me)$_2$ (59 mg, 0.2 mmol), NaOAc (33 mg, 0.4 mmol) were refluxed in CH_3CN (2 mL) for overnight. The volatiles were then removed under reduced pressure and the residue was purified by a silica gel flash column chromatography to afford (*S*)-**15a** (84 mg, 46% yield) and (*S*)-**15b** (68 mg, 37% yield). A single crystal grown from gold-complex (*S*)-**15a** or (*S*)-**15b** in a saturated solution of CH_2Cl_2/pentane was suitable for X-ray crystal analysis. (*S*)-**15a**: white

solid; $[\alpha]^{20}_{D}$ -64.7 (*c* 0.10, CH_2Cl_2); ^1H NMR (400 MHz, $CDCl_3$, TMS): δ 8.17-8.13 (m, 2H, ArH), 7.94 (d, *J* = 8.8 Hz, 1H, ArH), 7.90-7.88 (m, 1H, ArH), 7.74-7.70 (m, 1H, ArH), 7.66-7.59 (m, 3H, ArH), 7.56-7.50 (m, 2H, ArH), 7.42 (d, *J* = 8.4 Hz, 1H, ArH), 7.34-7.28 (m, 4H, ArH), 7.06 (s, 2H, ArH), 6.86-6.82 (m, 1H, ArH), 5.60 (d, *J* = 8.4 Hz, 1H, ArH), 3.78 (s, 3H. CH_3); ^{19}F NMR (376 MHz, $CDCl_3$, $CFCl_3$): δ -63.096; ^{13}C NMR (100 MHz, $CDCl_3$): δ 141.5, 140.8, 134.9, 134.5, 133.4, 132.9, 132.6, 131.3, 131.0, 130.9, 130.7, 129.9, 129.2, 129.1, 129.0, 128.5, 128.41, 128.37, 127.9, 127.6, 127.3, 126.9, 126.8, 126.4, 123.7, 123.4, 121.0, 120.6, 113.2, 111.4, 34.8. IR (CH_2Cl_2): ν 3059, 2926, 1594, 1385, 1346, 1277, 1182, 1133, 897, 820, 745, 713 cm^{-1}; MS (m/z): HRMS (ESI) Calcd. for $C_{36}H_{26}AuF_6IN_3$ ($[M+NH_4]^+$); 938.0736. Found: 938.0728.

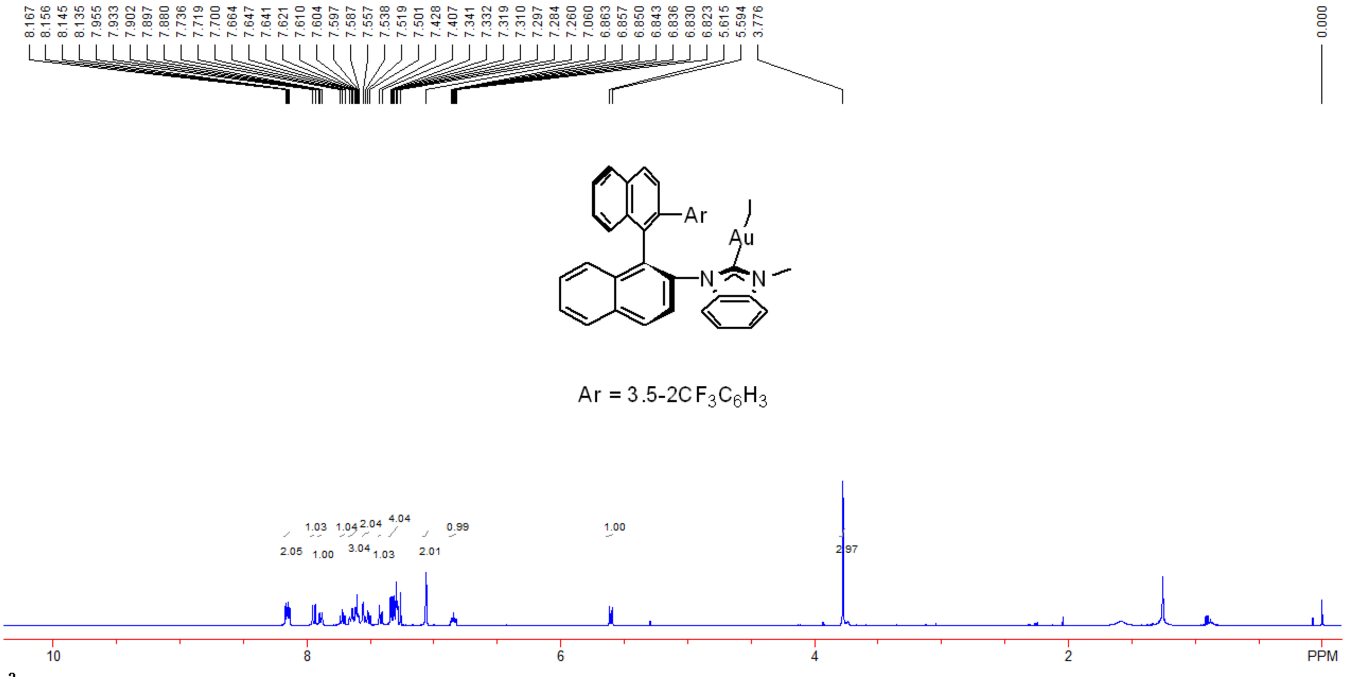

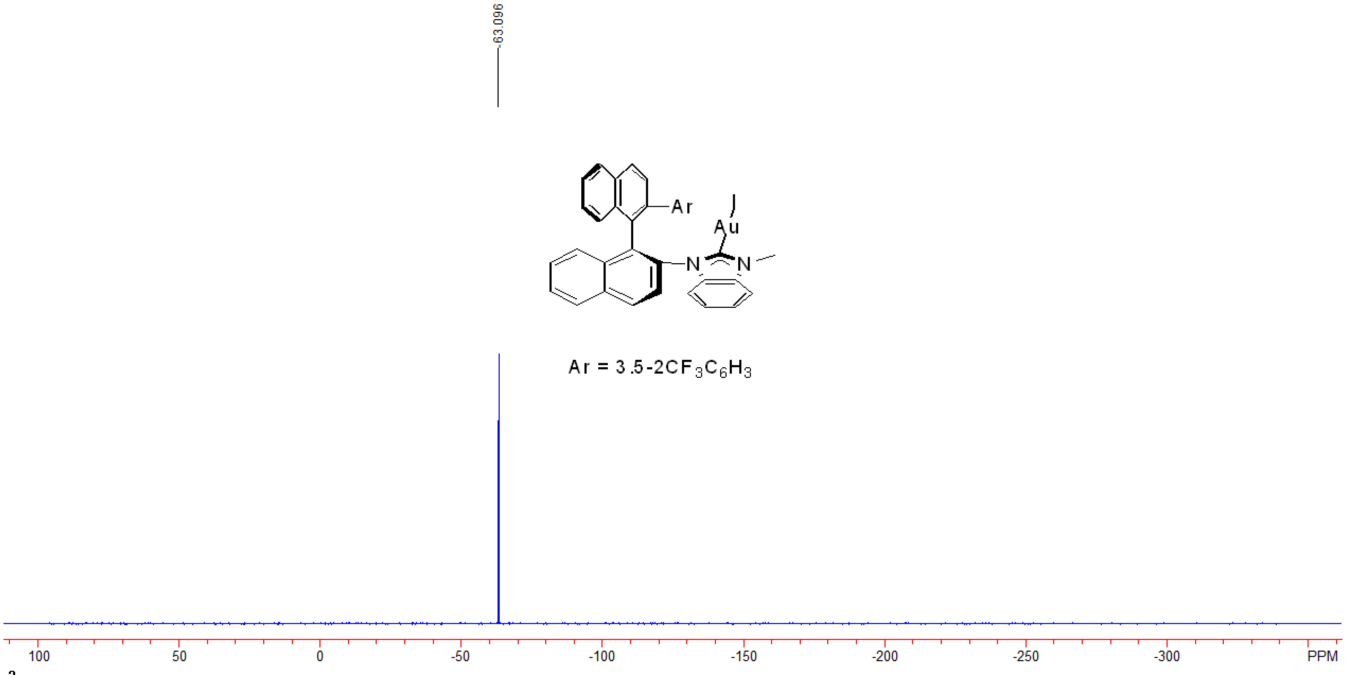

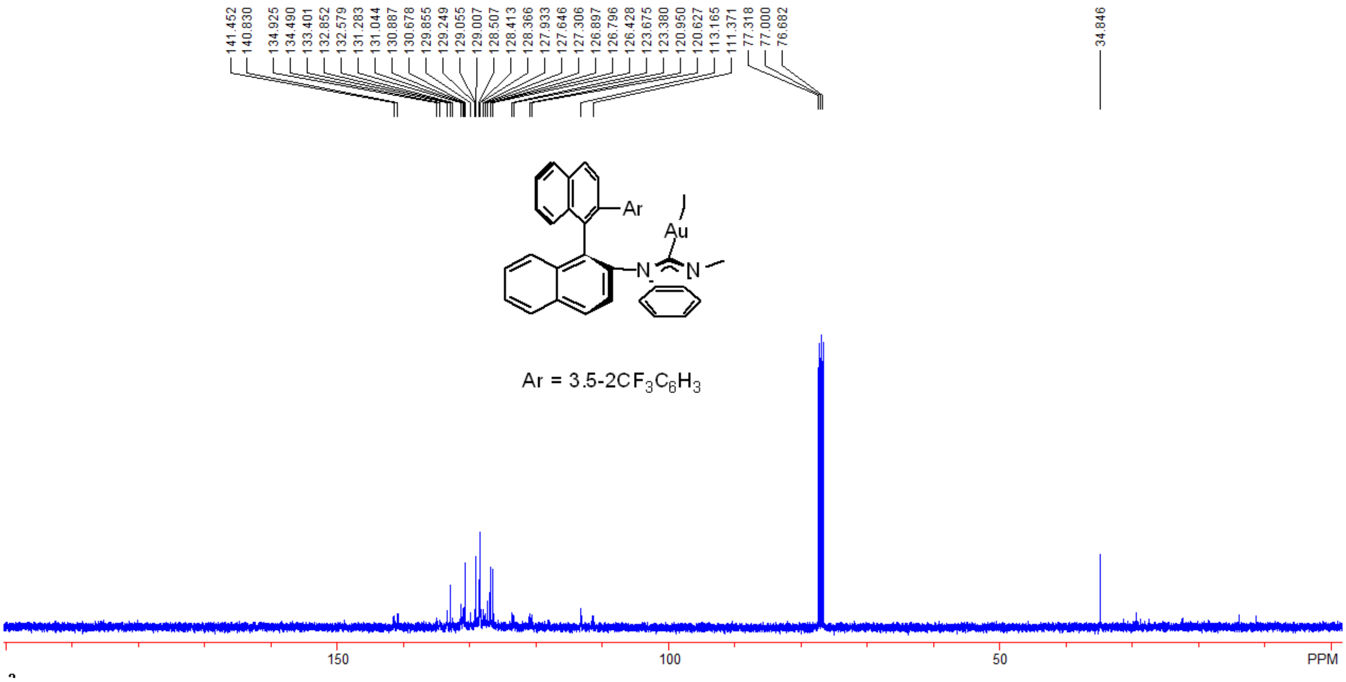

(S)-**15b**: white solid; $[\alpha]^{20}_{D}$ -66.1 (c 1.45, CH_2Cl_2); ^{1}H NMR (400 MHz, $CDCl_3$, TMS): δ 8.17 (d, J = 8.4 Hz, 1H, ArH), 8.13 (d, J = 8.0 Hz, 1H, ArH), 7.85 (d, J = 8.0 Hz, 1H, ArH), 7.79-7.69 (m, 4H, ArH), 7.63 (d, J = 8.0 Hz, 1H, ArH), 7.59-7.54 (m, 3H, ArH), 7.50-7.46 (m, 1H, ArH), 7.23 (d, J = 8.8 Hz, 2H, ArH), 7.17 (s, 2H, ArH), 7.08-7.04 (m, 1H, ArH), 6.47-6.42 (m ,2H, ArH), 3.94 (s, 3H. CH_3); ^{19}F NMR (376 MHz, $CDCl_3$, $CFCl_3$): δ -62.451; ^{13}C NMR (100 MHz, $CDCl_3$): δ 134.6, 134.5, 134.1,

134.0, 133.6, 131.4, 131.34, 131.27, 129.1, 129.0, 128.8, 128.7, 128.66, 128.58, 128.5, 128.3, 128.2, 127.8, 126.9, 126.5, 124.1, 123.4, 118.6, 31.9. IR (CH_2Cl_2): ν 2923, 2851, 1726, 1465, 1387, 1277, 1181, 1131, 894, 823, 743, 712, 681 cm^{-1}; MS (m/z): HRMS (ESI) Calcd. for $C_{36}H_{26}AuF_6IN_3$ ([M +NH_4]$^+$); 938.0736. Found: 938.0725.

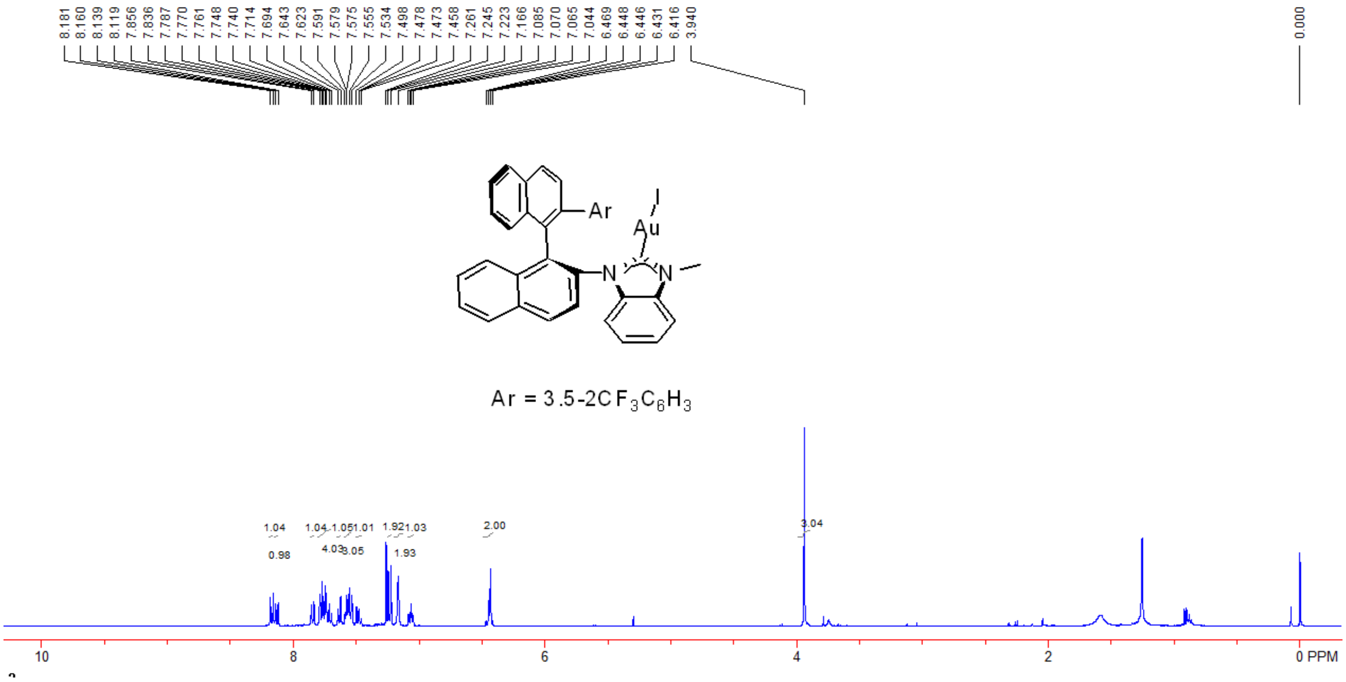

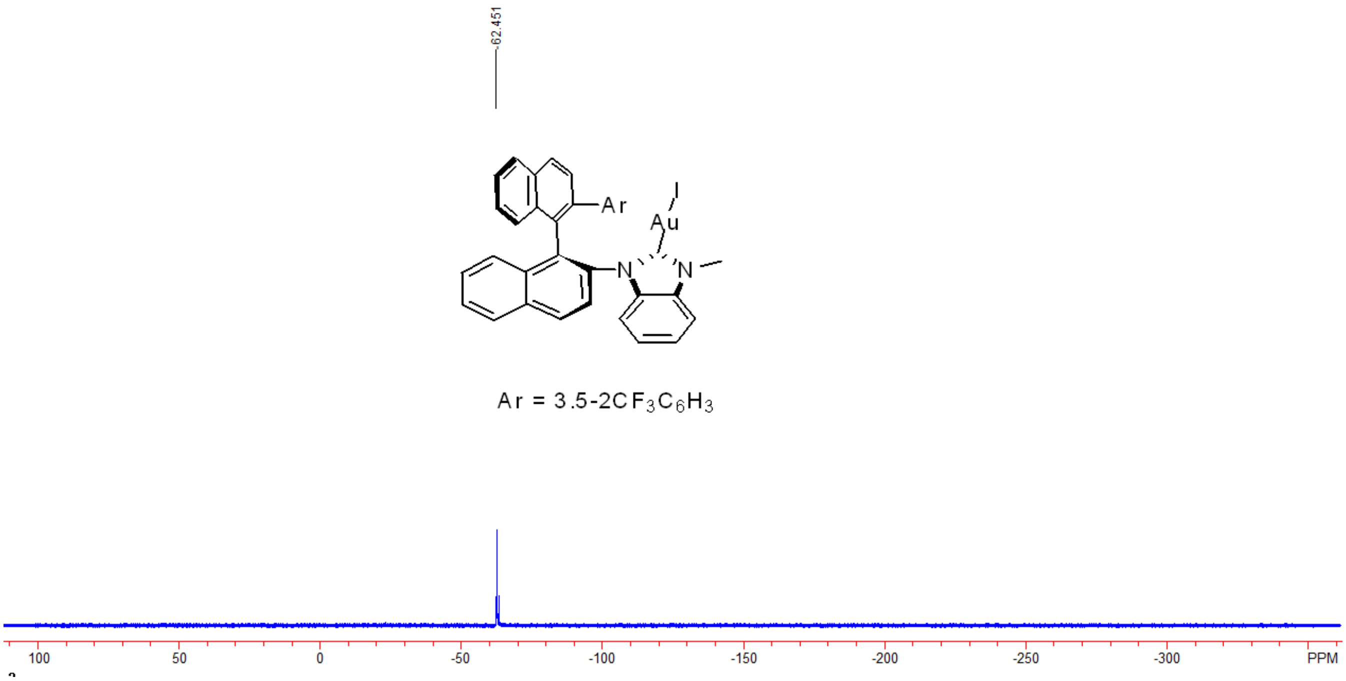

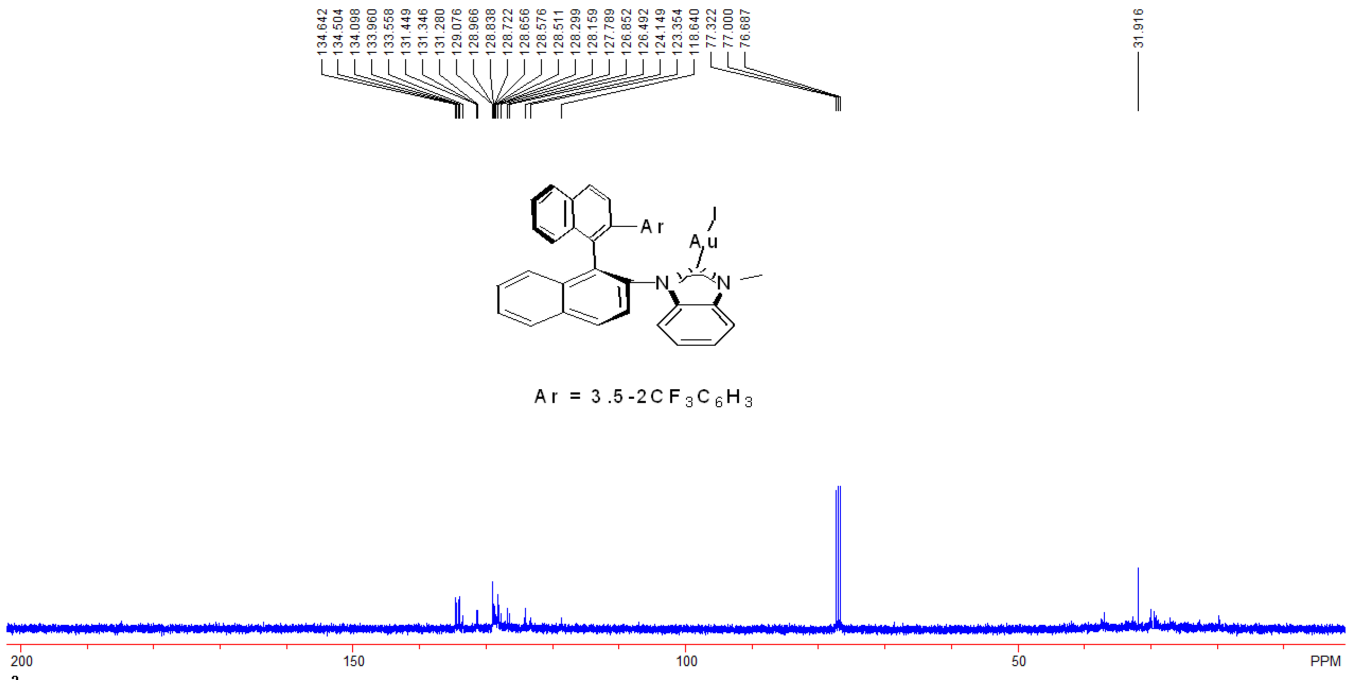

Synthesis of gold complex (*S*)-16

The compound (*S*)-**14** (152 mg, 0.2 mmol) and AuCl·S(Me)$_2$ (59 mg, 0.2 mmol), NaOAc (33 mg, 0.4 mmol) were refluxed in CH_3CN (2 mL) for overnight. The volatiles were then removed under reduced pressure and the residue was purified by a silica gel flash column chromatography. Gold complex (S)**-16** (142 mg, 75% total yield) was obtained as mixture: white solid; $[\alpha]^{20}_{D}$ -82.2 (*c* 0.75, CH_2Cl_2); ^{1}H NMR (400 MHz, $CDCl_3$, TMS): δ 8.21-8.11 (m, 5H, ArH), 8.02 (d, *J* = 8.8 Hz, 2H, ArH), 7.95 (d, *J* = 8.4 Hz, 2H, ArH), 7.85-7.42 (m, 29H, ArH), 7.35-7.26 (m, 15H, ArH), 7.20-7.18 (m, 2H, ArH), 7.14-7.13 (m, 4H, ArH), 7.06 (d, *J* = 8.0 Hz, 1H, ArH), 6.95-6.92 (m, 5H, ArH), 6.80-6.75 (m, 6H, ArH), 6.52 (d, *J* = 8.0 Hz, 1H, ArH), 6.47-6.43 (m, 1H, ArH), 5.84 (d, *J* = 15.6 Hz, 1H, CH_2), 5.75-5.72 (m, 4H, ArH and CH_2), 5.36 (d, *J* = 15.6 Hz, 1H, CH_2), 5.14 (d, *J* = 15.2 Hz, 2H, CH_2); ^{19}F NMR (376 MHz, $CDCl_3$, $CFCl_3$): δ -62.571, -63.093; ^{13}C NMR (100 MHz, $CDCl_3$): δ 142.4, 135.3, 134.45, 134.38, 133.3, 132.8, 132.5, 130.70, 130.66, 130.59, 130.0, 129.8, 129.2, 129.0, 128.9, 128.8, 128.7, 128.5, 128.4, 127.9, 127.8, 127.68, 127.56, 127.4, 127.2, 127.14, 127.05, 126.9, 126.8, 126.6, 125.4, 124.53, 124.46, 124.2, 123.0, 120.5, 111.5, 110.8, 52.6. IR (CH_2Cl_2): ν 3061, 2924, 1594, 1478, 1406, 1278, 1182, 1133, 897,

820, 745, 706 cm^{-1}; MS (m/z): HRMS (ESI) Calcd. for $C_{42}H_{30}AuBrF_6N_3$ ([M +NH$_4$]$^+$); 966.1187. Found: 966.1185.

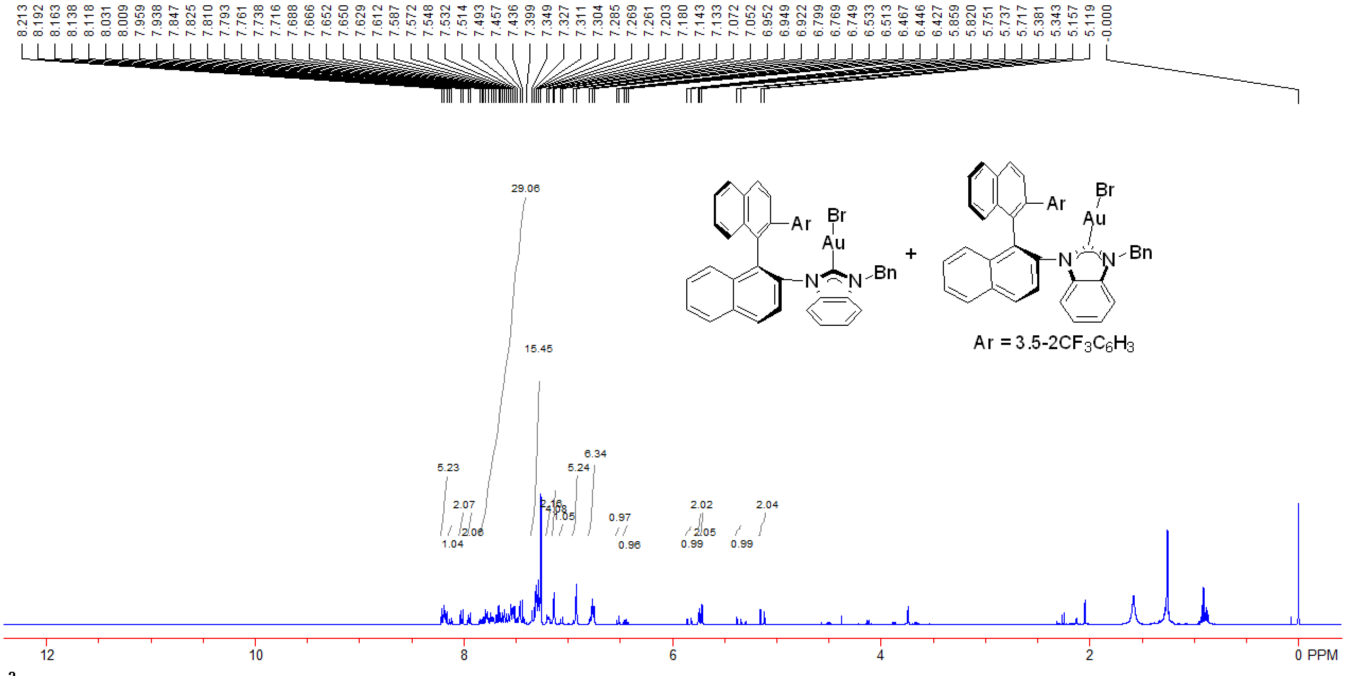

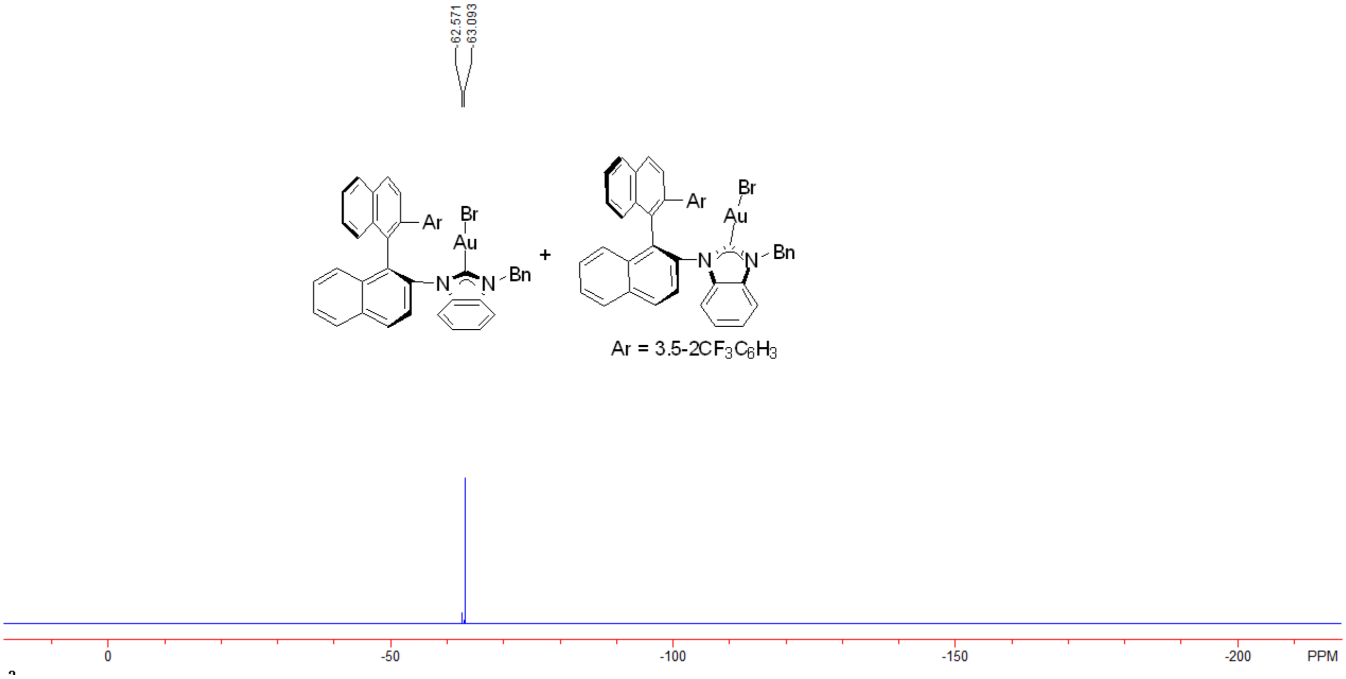

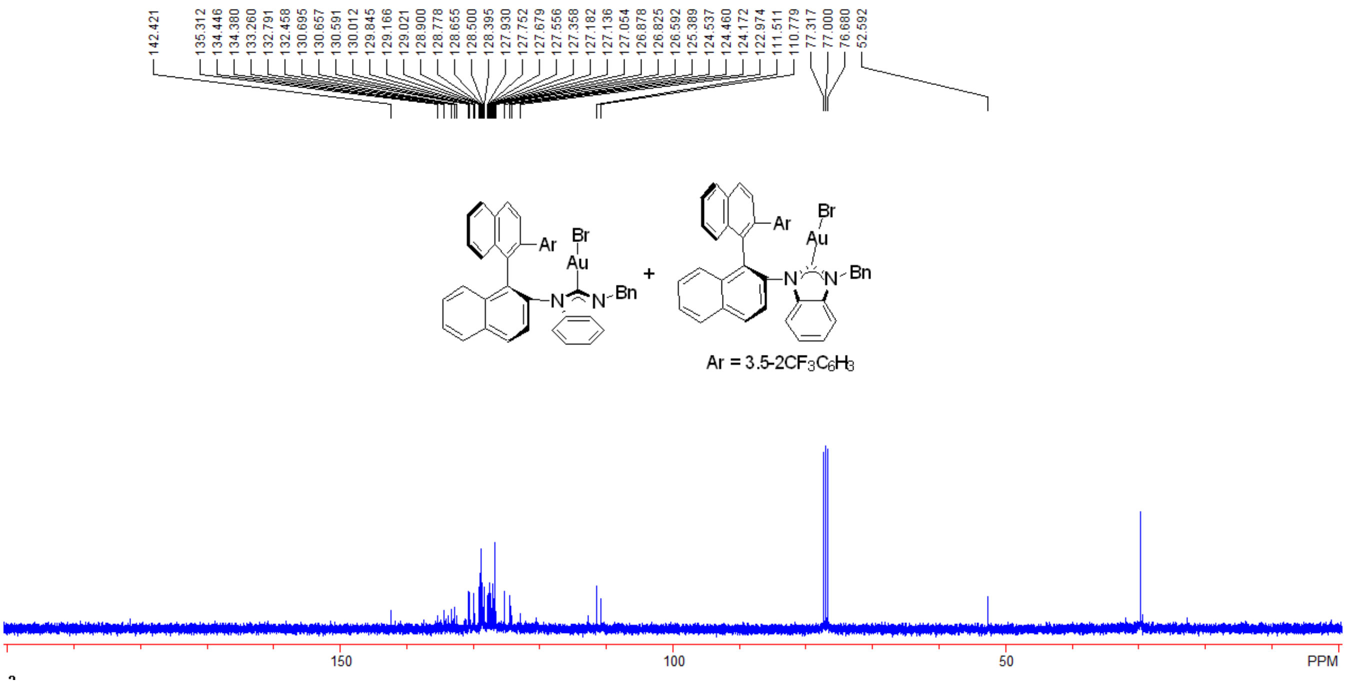

The synthesis of P–Au(I) complexes

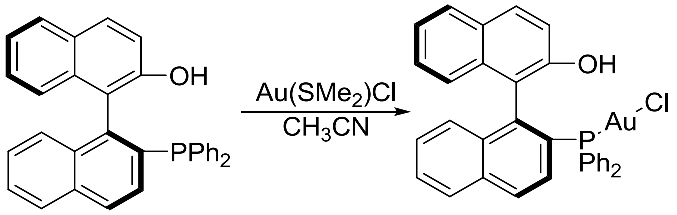

17 **18** (88% yield)

(S)-2'-(diphenylphosphino)-1,1'-binaphthyl-2-ol (**17**) was prepared according to published literature procedures.[2]

Synthesis of gold-complex (*S*)-18

The compound (*S*)-**17** (454 mg, 1.0 mmol) and AuCl·S(Me)$_2$ (294 mg, 1.0 mmol) were stirred in CH_3CN (10 mL) for overnight. The volatiles were then removed under reduced pressure and the residue was purified by a silica gel flash column chromatography to afford pure product (*S*)-**18** (603 mg, 88% yield). A single crystal grown from gold complex (*S*)-**18** in a saturated solution of CH_2Cl_2/pentane was

suitable for X-ray crystal analysis. white solid; $[\alpha]^{20}_{D}$ -35.4 (*c* 0.20, CH_2Cl_2); ^{1}H NMR (400 MHz, $CDCl_3$, TMS): δ 7.99-7.93 (m, 3H, ArH), 7.80 (d, *J* = 8.4 Hz, 1H, ArH), 7.60-7.56 (m, 1H, ArH), 7.50-7.45 (m, 3H, ArH), 7.42-7.17 (m, 12H, ArH), 6.86-6.82 (m, 1H, ArH), 6.45 (d, *J* = 8.4 Hz, 1H, ArH), 5.17 (br, 1H, OH); ^{31}P NMR (162 MHz, $CDCl_3$, 85% H_3PO_4): δ 26.116; ^{13}C NMR (100 MHz, $CDCl_3$): δ 141.8, 136.5, 134.4, 133.7, 133.24, 133.22, 133.1, 132.39, 132.35, 130.5, 130.2, 129.8, 129.0, 128.9, 128.6, 128.4, 127.6, 127.3, 127.2, 127.1, 127.0, 126.6, 124.2, 123.7, 123.0, 112.6, 110.4. IR (CH_2Cl_2): ν 3359, 3055, 2924, 1623, 1513, 1435, 1269, 1098, 972, 937, 814, 743, 692 cm^{-1}; MS (m/z): HRMS (ESI) Calcd. for $C_{32}H_{27}AuClNOP$ ($[M+NH_4]^+$); 704.1179. Found: 704.1170.

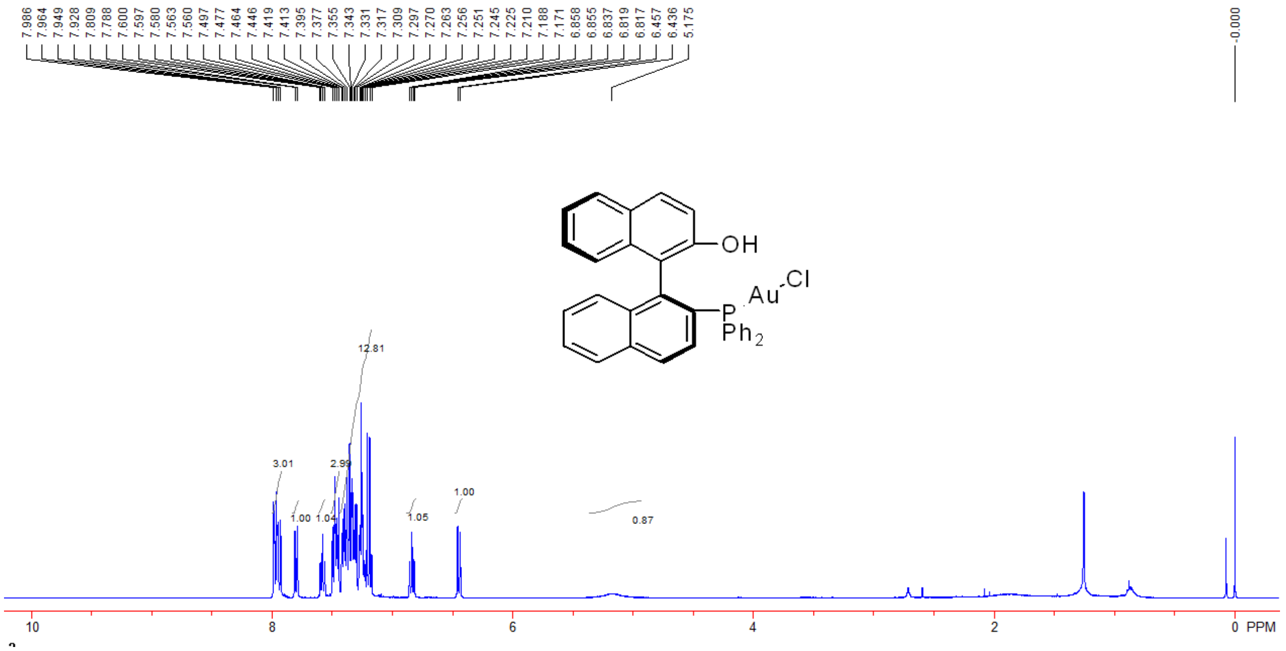

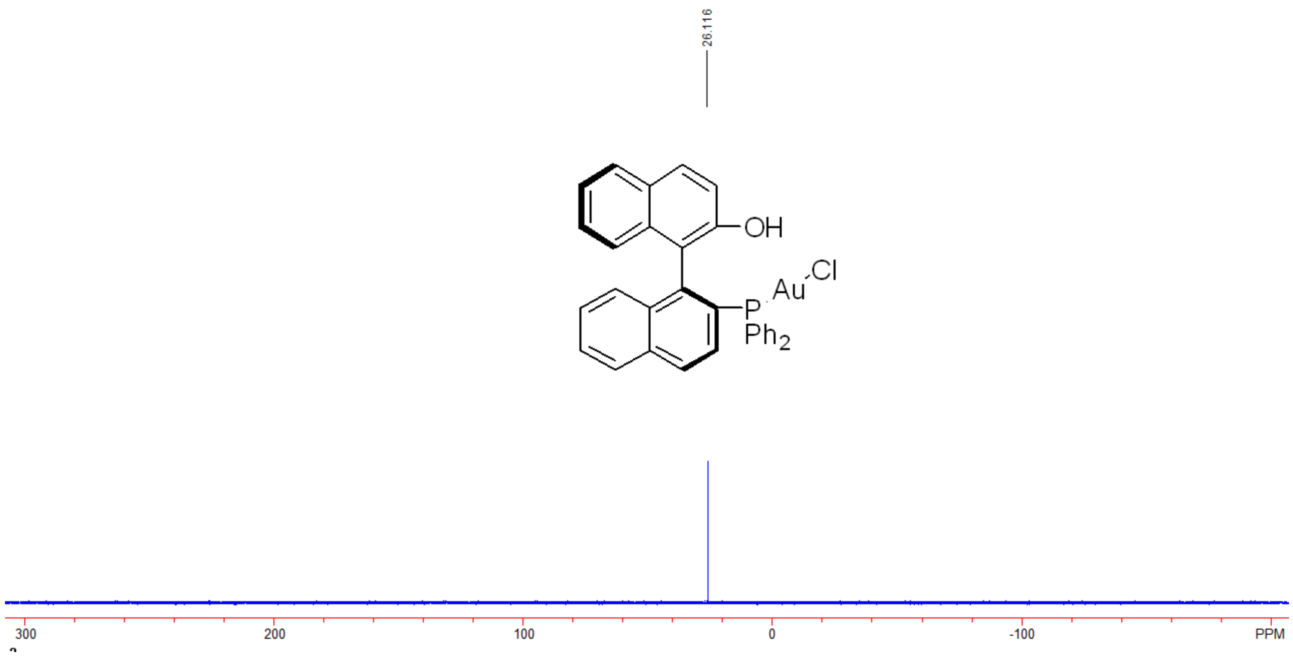
26.116
OH
Au
Cl
P
Ph2
300
200
100
0
-100
PPM

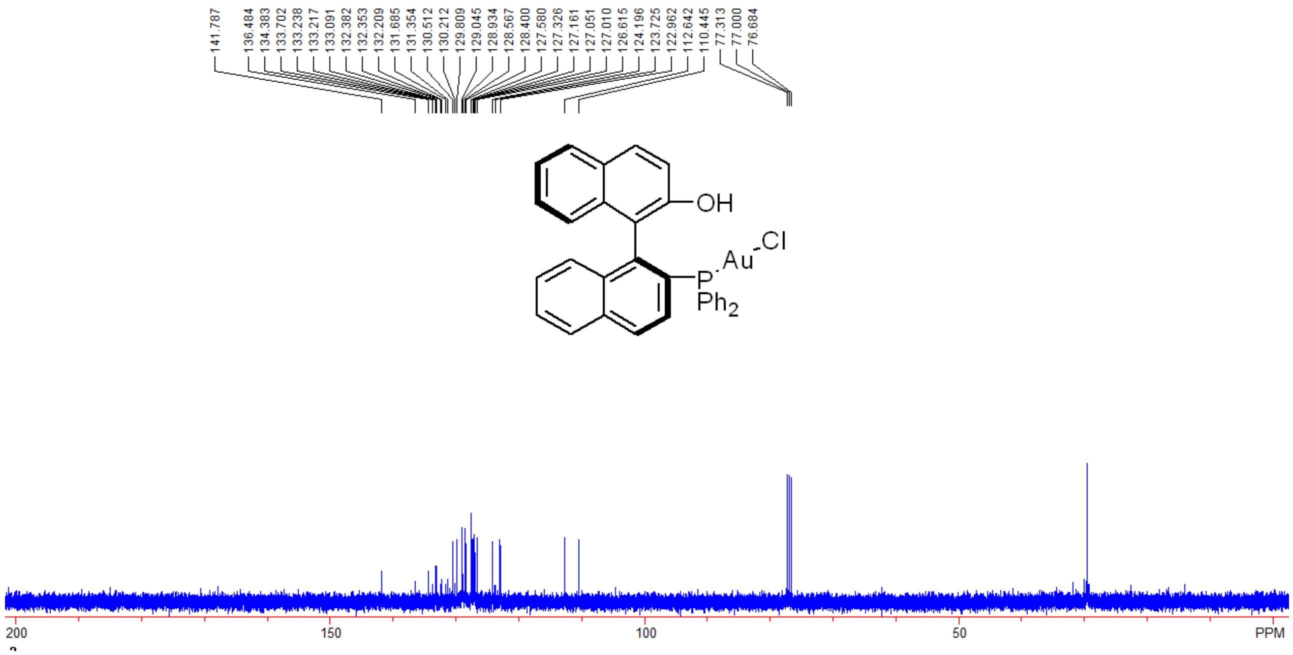
141.787
136.484
134.383
133.702
133.238
133.217
133.091
132.382
132.353
132.209
131.685
131.354
130.512
130.212
129.809
129.045
128.934
128.567
128.400
127.580
127.326
127.161
127.051
127.010
126.615
124.196
123.725
122.962
112.642
110.445
77.313
77.000
76.684
OH
Au
Cl
P
Ph2
200
150
100
50
PPM

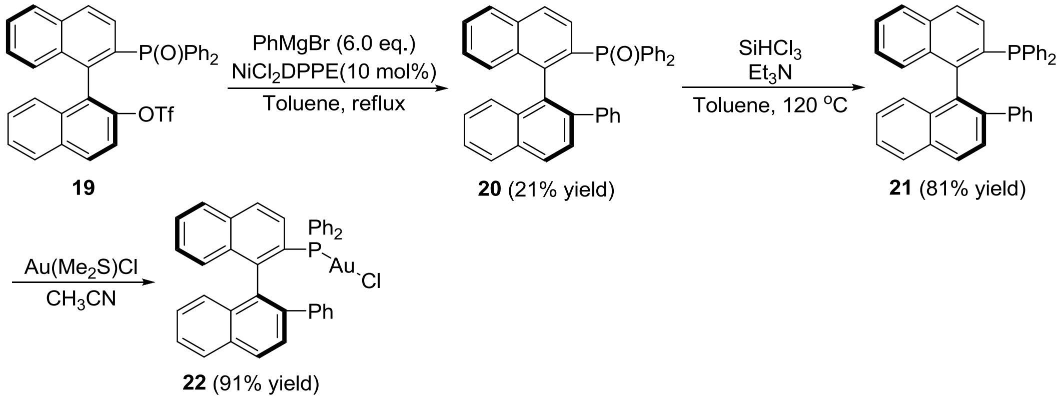

(S)-2'-(diphenylphosphoryl)-1,1'-binaphthyl-2-yl trifluoromethanesulfonate **19** was prepared according to the previous literature procedures.[2]

Synthesis of (*S*)-diphenyl(2'-phenyl-[1,1'-binaphthalen]-2-yl)phosphine oxide (20)

(S)-2'-(diphenylphosphoryl)-1,1'-binaphthyl-2-yl trifluoromethanesulfonate **19** (3.01 g, 5 mmol) and $NiCl_2$(dppe) (285 mg, 0.5 mmol) was dissolved into toluene (40 mL) under argon. To this solution was added dropwise a 1 M THF solution of phenylmagnesium bromide (30 mL, 30 mmol) at 0 °C, and stirring was continued under reflux for overnight. The reaction was quenched with saturated NH_4Cl carefully and extractive workup was conducted with EA. The organic extracts were washed with brine and dried over anhydrous Na_2SO_4. Removal of solvents and purification of the residual oil by column chromatography on silica gel afforded compound (*S*)-**20** (556 mg, 21% yield) as yellow powder. This is a new compound. white solid; $[\alpha]^{20}_D$ -168.3 (*c* 0.80, CH_2Cl_2); ^{1}H NMR (400 MHz, $CDCl_3$, TMS): δ 7.88 (d, *J* = 8.4 Hz, 1H, ArH), 7.85-7.82 (m, 2H, ArH), 7.78 (d, *J* = 8.0 Hz, 1H, ArH), 7.56-7.47 (m, 3H, ArH), 7.35-7.28 (m, 5H, ArH), 7.17-7.00 (m, 12H, ArH), 6.94 (t, *J* = 8.0 Hz, 2H, ArH), 6.87 (d, *J* = 8.8 Hz, 1H, ArH). ^{31}P NMR (162 MHz, $CDCl_3$, 85% H_3PO_4): δ 33.064; ^{13}C NMR (100 MHz, $CDCl_3$): δ 143.7, 143.6, 141.2, 139.1, 134.7, 134.6, 133.85, 133.82, 133.2, 132.7, 132.34, 132.29, 132.14, 132.09, 131.7, 131.49, 131.47, 131.39, 131.38, 131.30, 131.28, 131.17, 131.14, 131.09, 129.4, 128.8, 128.7, 128.6, 128.3, 128.1, 128.04, 128.02, 127.9, 127.8, 127.7, 127.64, 127.62, 127.47, 127.44, 127.3, 127.27,

127.1, 126.8, 126.3, 126.0, 125.3; IR (CH_2Cl_2): ν 3054, 1591, 1495, 1436, 1200, 1115, 1028, 873, 819, 762, 722, 694 cm^{-1}; MS (m/z): HRMS (ESI) Calcd. for $C_{38}H_{28}OP$ ($[M+H]^+$); 531.1872. Found: 531.1865.

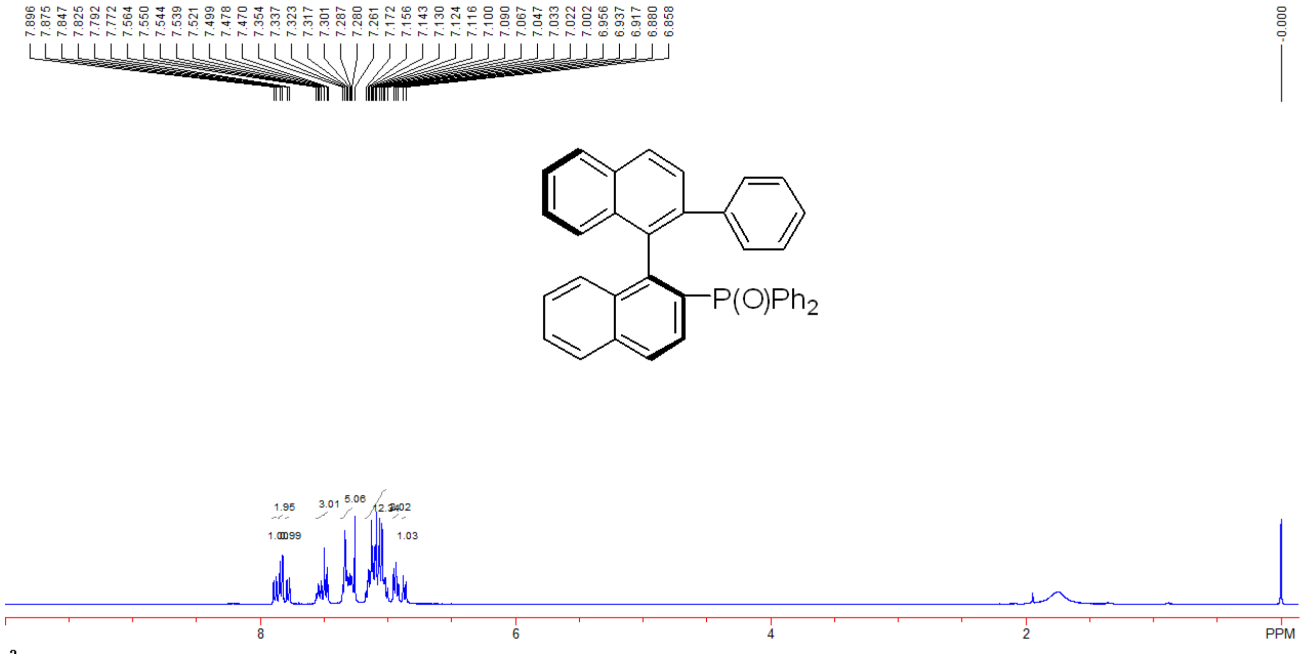

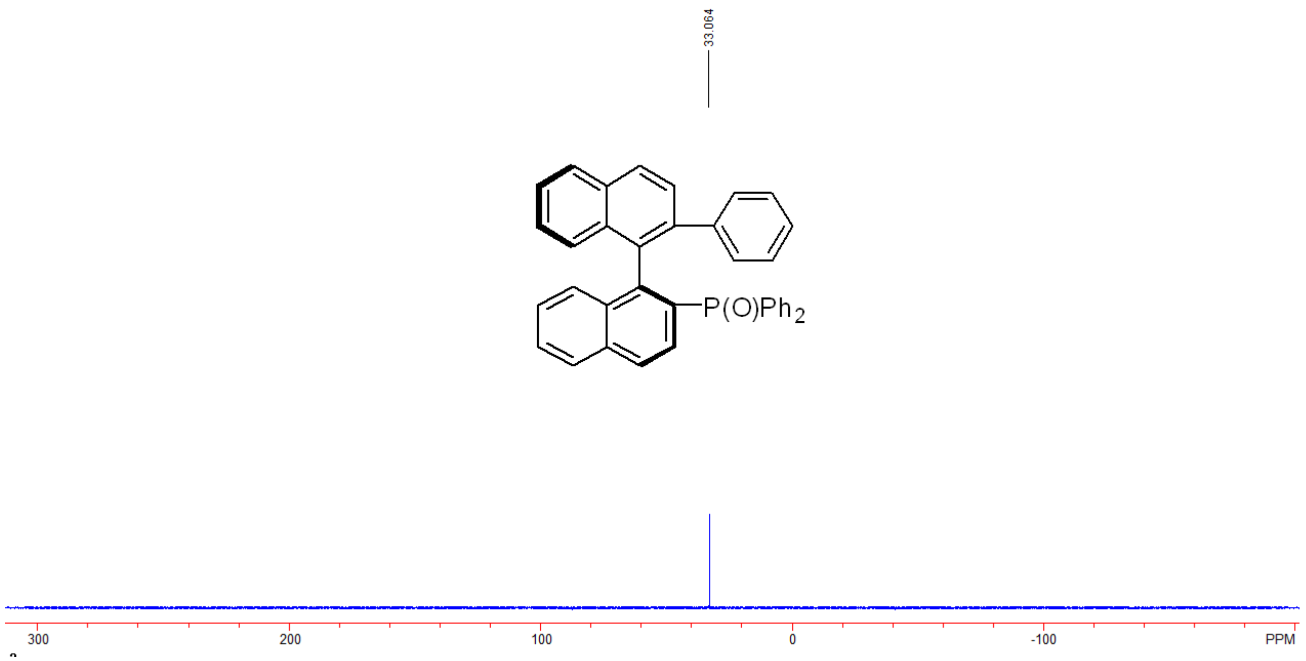

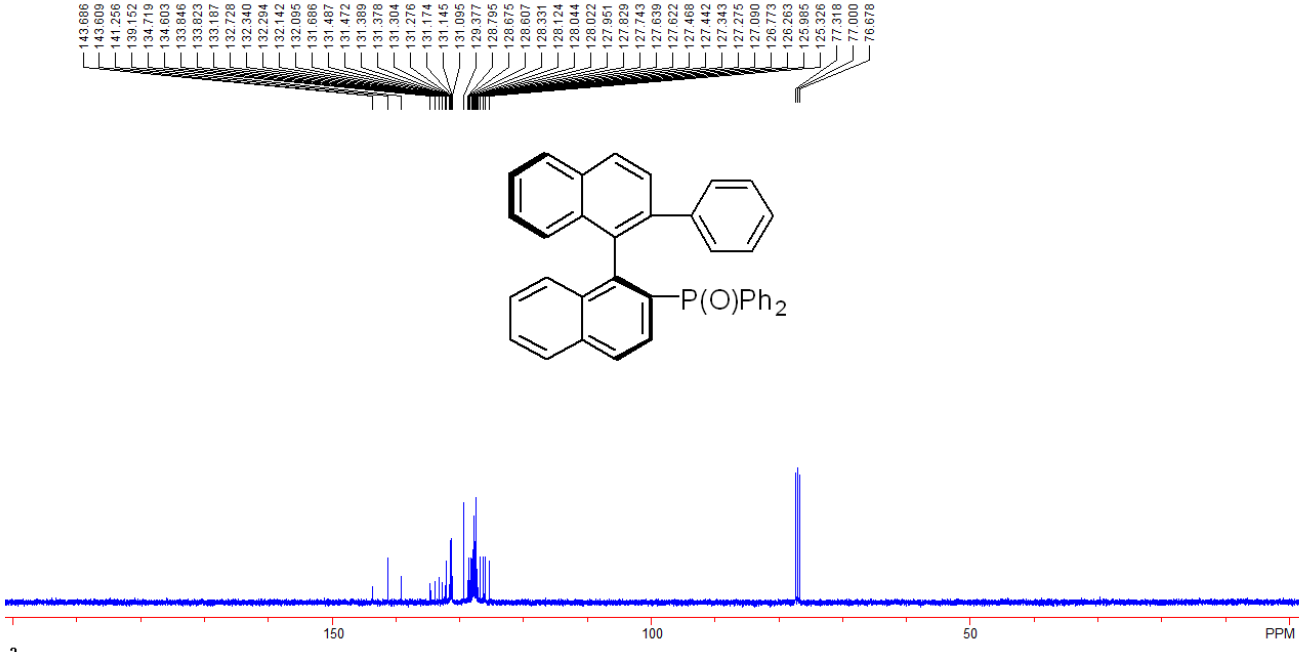

Synthesis of (*S*)-diphenyl(2'-phenyl-[1,1'-binaphthalen]-2-yl)phosphine (21)

Et_3N (0.3 mL, 2 mmol) was added dropwise to a solution of compound (*S*)-**20** in toluene (530 mg, 1.0 mmol) under argon. This solution was added $SiHCl_3$ (0.2 mL, 2.0 mmol) under an ice-water bath, and stirring was continued at 120 ^{o}C for overnight. The reaction was quenched with saturated NH_4Cl carefully and extractive workup was conducted with EA. The organic extracts were washed with brine and dried over anhydrous Na_2SO_4. Removal of solvents and purification of the residual oil by column

chromatography on silica gel afforded compound (*S*)-**21** (416 mg, 81% yield) as yellow powder. This is a known compound. white solid; ^{1}H NMR (400 MHz, $CDCl_3$, TMS): δ 8.04 (d, *J* = 8.4 Hz, 1H, ArH), 7.89 (d, *J* = 8.0 Hz, 1H, ArH), 7.83 (d, *J* = 8.0 Hz, 1H, ArH), 7.72 (d, *J* = 8.4 Hz, 1H, ArH), 7.65 (d, *J* = 8.4 Hz, 1H, ArH), 7.46 (t, *J* = 8.0 Hz, 1H, ArH), 7.42 (d, *J* = 8.8 Hz, 1H, ArH), 7.35-7.28 (m, 2H, ArH), 7.23-7.20 (m, 1H, ArH), 7.14 (d, *J* = 7.2 Hz, 1H, ArH), 7.10-7.01 (m, 8H, ArH), 6.97-6.88 (m, 5H, ArH), 6.77 (d, *J* = 8.8 Hz, 1H, ArH), 6.60 (t, *J* = 7.2 Hz, 2H, ArH). ^{31}P NMR (162 MHz, $CDCl_3$, 85% H_3PO_4): δ -13.790; ^{13}C NMR (100 MHz, $CDCl_3$): δ 144.8, 144.5, 141.8, 139.55, 139.52, 138.2, 138.0, 137.5, 137.4, 135.2, 135.1, 134.9, 134.8, 134.5, 134.4, 133.9, 133.7, 133.36, 133.35, 133.02, 133.01, 132.38, 132.37, 132.2, 130.7, 129.4, 128.4, 128.3, 128.2, 128.0, 127.98, 127.94, 127.90, 127.89, 127.86, 127.7, 127.60, 127.57, 127.4, 127.2, 126.6, 126.3, 125.9, 125.4; IR (CH_2Cl_2): ν 3053, 1585, 1494, 1434, 1264, 1206, 1115, 1027, 894, 818, 739, 697, 675 cm^{-1}; MS (m/z): HRMS (ESI) Calcd. for $C_{38}H_{28}P$ ($[M+H]^+$); 515.1923. Found: 515.1921.

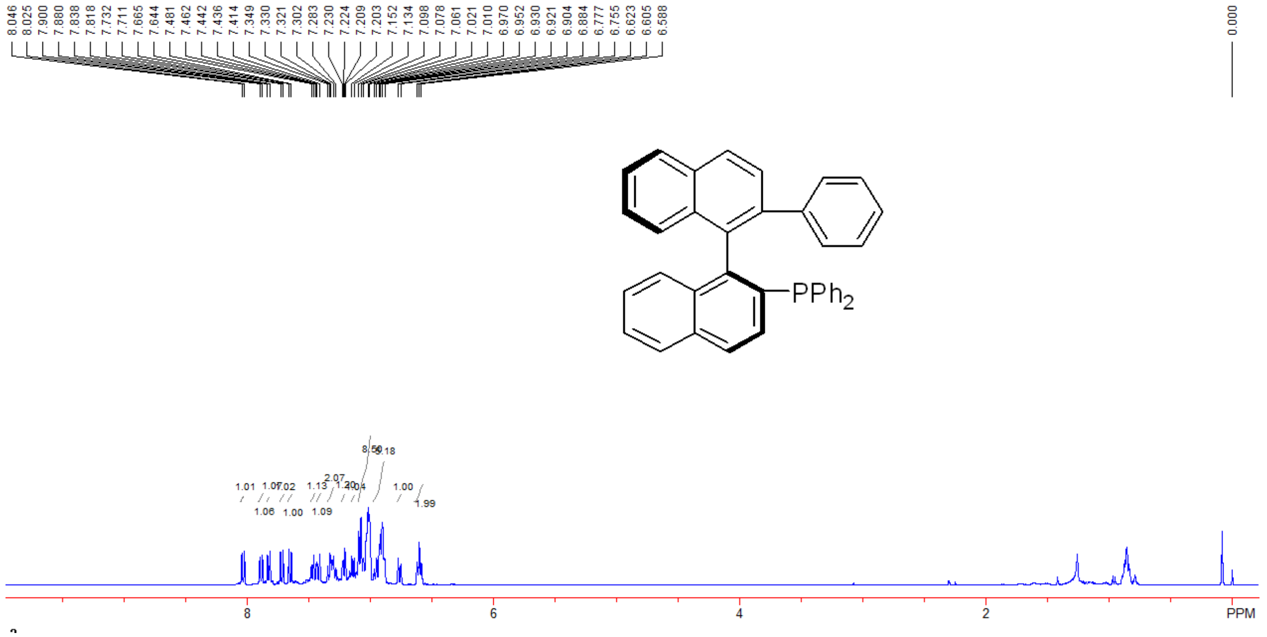

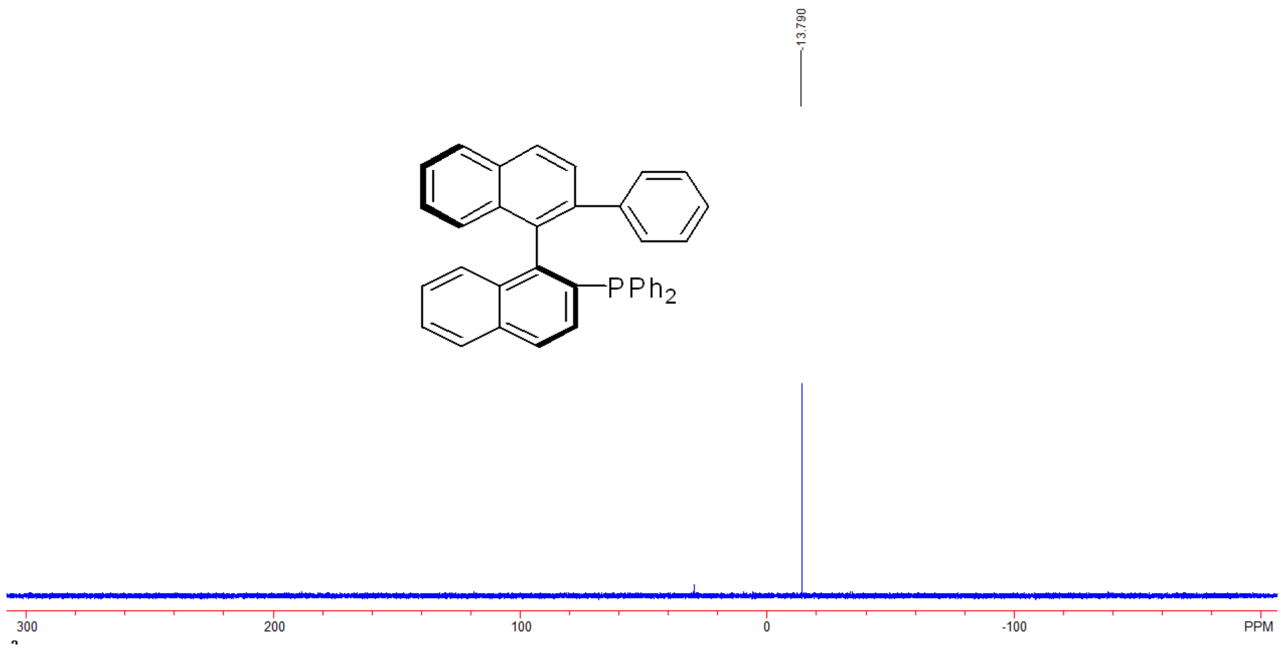

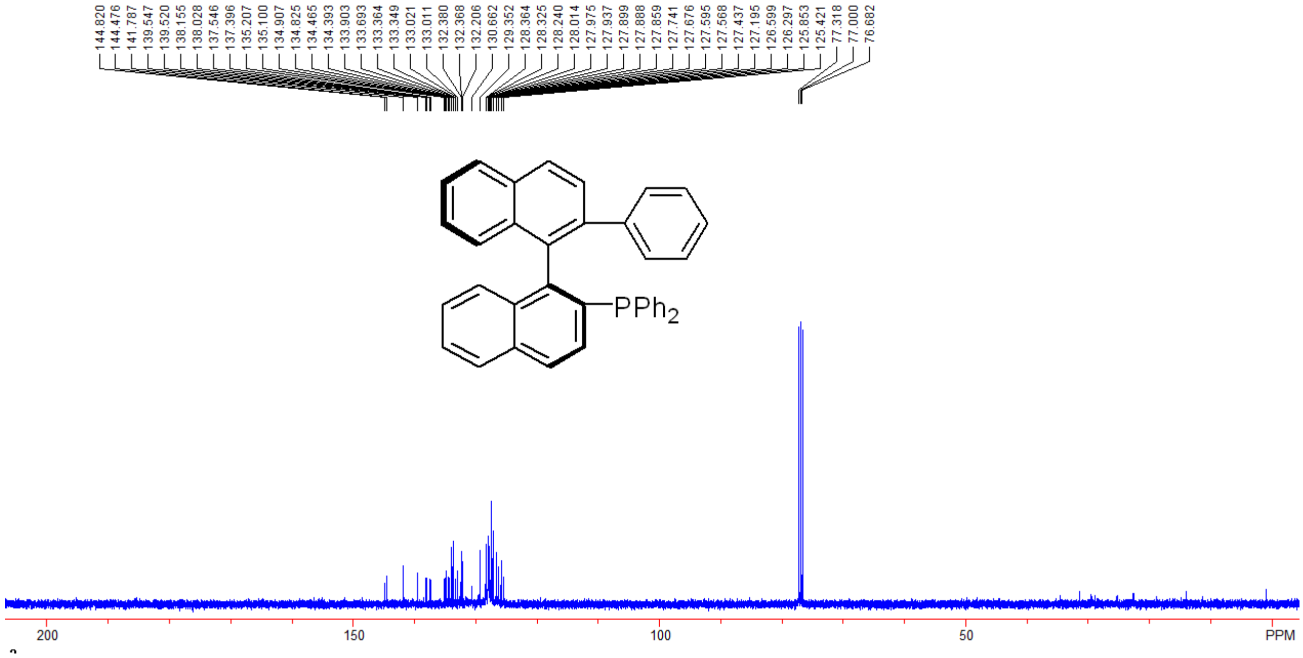

Synthesis of gold complex (*S*)-22

The compound (*S*)-**21** (257 mg, 0.5 mmol) and AuCl·S(Me)$_2$ (147 mg, 0.5 mmol) were stirred in CH_3CN (5 mL) for overnight. The volatiles were then removed under reduced pressure and the residue was purified by a silica gel flash column chromatography to afford (*S*)-**22** (339 mg, 91% yield). A single crystal grown from gold complex **22** in a solution of CH_2Cl_2/pentane was suitable for X-ray crystal

analysis. white solid; $[\alpha]^{20}_{D}$ -80.7 (*c* 0.95, CH_2Cl_2); ^{1}H NMR (400 MHz, $CDCl_3$, TMS): δ 8.27 (d, *J* = 8.8 Hz, 1H, ArH), 8.05 (d, *J* = 8.4 Hz, 1H, ArH), 7.90 (d, *J* = 8.0 Hz, 1H, ArH), 7.84 (d, *J* = 8.4 Hz, 1H, ArH), 7.67 (d, *J* = 8.4 Hz, 1H, ArH), 7.62-7.57 (m, 1H, ArH), 7.47 (t, *J* = 7.6 Hz, 1H, ArH), 7.40-7.35 (m, 4H, ArH), 7.28-7.24 (m, 2H, ArH), 7.22-7.12 (m, 6H, ArH), 6.97 (d, *J* = 7.6 Hz, 2H, ArH), 6.92 (t, *J* = 7.2 Hz, 2H, ArH), 6.88 (d, *J* = 7.6 Hz, 1H, ArH), 6.84 (d, *J* = 8.8 Hz, 1H, ArH), 6.77 (t, *J* = 7.6 Hz, 2H, ArH); ^{31}P NMR (162 MHz, $CDCl_3$, 85% H_3PO_4): δ 22.898, 22.825, 22.751, 22.697; ^{13}C NMR (100 MHz, $CDCl_3$): δ 151.4, 134.6, 134.4, 134.1, 133.9, 133.7, 133.5, 133.2, 133.1, 131.5, 131.44, 131.37, 131.35, 131.25, 129.9, 129.3, 129.04, 128.98, 128.92, 128.89, 128.87, 128.85, 128.84, 128.7, 128.64, 128.59, 128.51, 128.4, 128.3, 128.2, 127.8, 127.5, 126.81, 126.80, 126.6, 126.5, 126.47, 126.1, 126.0, 124.1, 123.4. IR (CH_2Cl_2): ν 3054, 1589, 1494, 1480, 1436, 1306, 1265, 1098, 1027, 819, 763, 744, 698 cm^{-1}; MS (m/z): HRMS (ESI) Calcd. for $C_{38}H_{31}AuClNP$ ($[M+NH_4]^+$); 764.1543. Found: 764.1532.

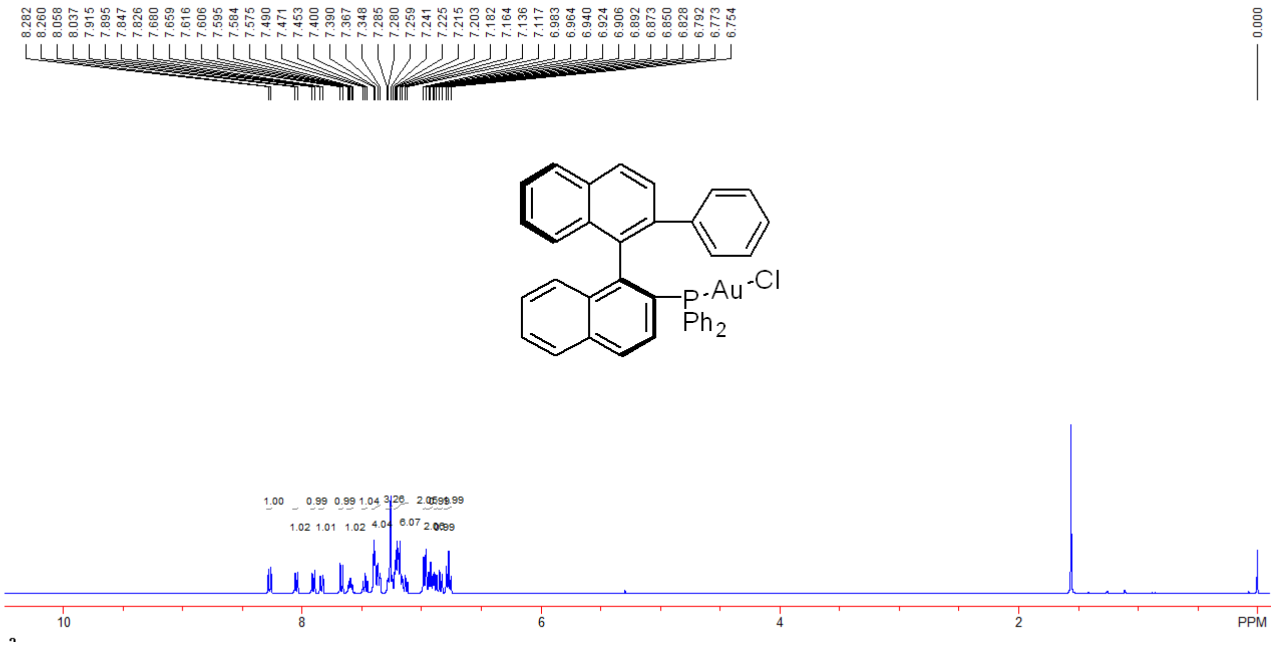

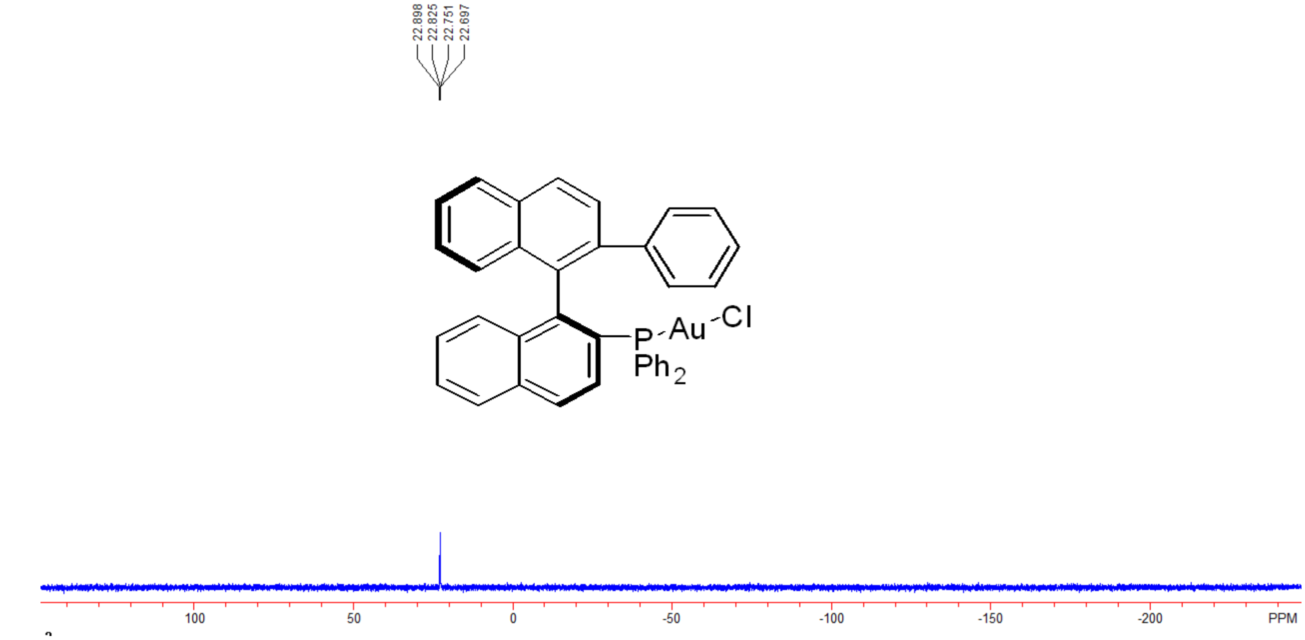
22.898
22.825
22.751
22.697
P
Au
Cl
Ph2
100
50
0
-50
-100
-150
-200
PPM

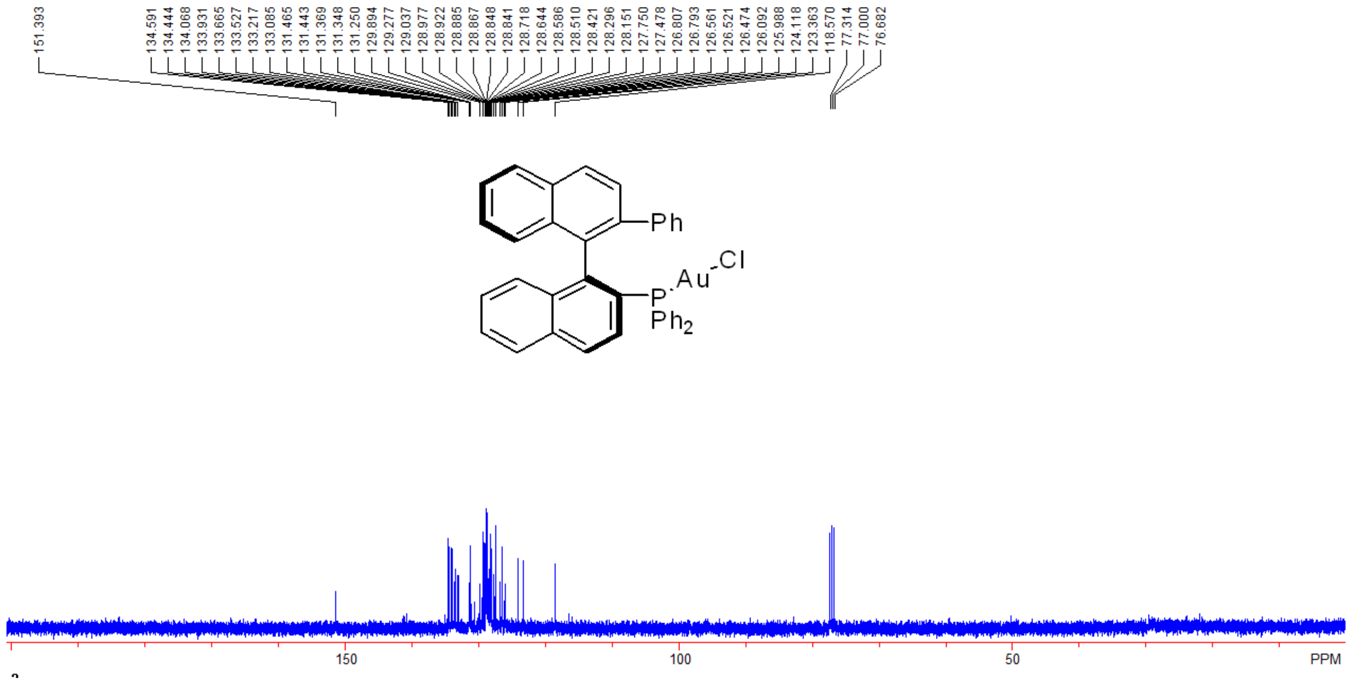
151.393
134.591
134.444
134.068
133.931
133.665
133.527
133.217
133.085
131.465
131.443
131.369
131.348
131.250
129.894
129.277
129.037
128.977
128.922
128.885
128.867
128.848
128.841
128.718
128.644
128.586
128.510
128.421
128.296
128.151
127.750
127.478
126.807
126.793
126.561
126.521
126.474
126.092
125.988
124.118
123.363
118.570
77.314
77.000
76.682
Ph
P
Au
Cl
Ph2
150
100
50
PPM

Typical procedure for intramolecular hydroamination reaction

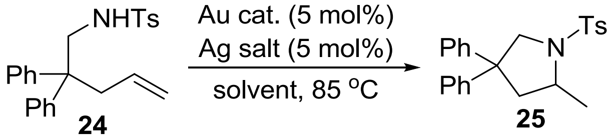

A mixture of Au catalyst (5 mol %) and AgX (5 mol %) in solvent (0.5 mL) was stirred at room temperature for 5 min under argon, then a solution of compound **24** (76.6 mg, 0.20 mmol) in solvent (0.5 mL) was added into the resulting solution. The resulting suspension was stirred at 85 °C for 36 h. Column chromatography of the reaction mixture gave the desired product. The enantiomeric purity of the product was determined by chiral HPLC analysis.

Compound **24**[3] was prepared according to the previously reported method.

2-methyl-4,4-diphenyl-1-tosylpyrrolidine (**25**): ^{1}H NMR (400MHz, $CDCl_3$, TMS): δ 7.61 (d, J = 8.0 Hz, 2H, ArH), 7.27-7.09 (m, 12H, ArH), 4.17 (d, J = 10.4 Hz, 1H, CH_2), 3.94 (dd, J_1 = 10.4 Hz, J_2 = 0.4 Hz, 1H, CH_2), 3.82-3.74 (m, 1H, CH), 2.78 (ddd, J_1 = 12.4 Hz, J_2 = 7.2 Hz, J_3 = 0.4 Hz, 1H, CH_2), 2.38 (s, 3H, CH_3), 2.26 (dd, J_1 = 12.4 Hz, J_2 = 7.2 Hz, 1H, CH_2), 1.25 (d, J = 6.4 Hz, 3H, CH_3); ^{13}C NMR (100 MHz, $CDCl_3$): δ 145.6, 144.8, 142.9, 135.3, 129.5, 128.43, 128.42, 127.1, 126.6, 126.42, 126.39, 126.2, 58.3, 55.4, 52.2, 45.9, 22.1, 21.4; $[\alpha]^{20}_{D}$ 20.1 (c 1.2, CH_2Cl_2), for 29% ee; Chiralcel PA-2, hexane/i-PrOH = 60/40, 0.5 mL/min, 214 nm, t_{major} = 45.07 min, t_{minor} = 27.49 min.

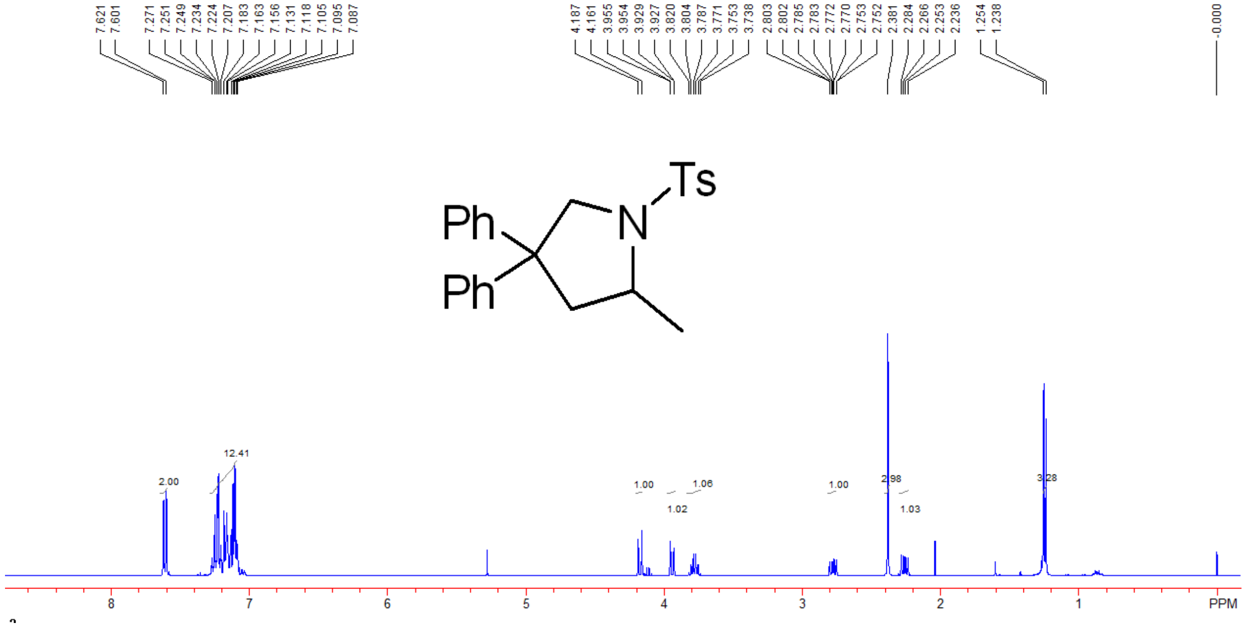

7.621
7.601
7.271
7.251
7.249
7.234
7.224
7.207
7.183
7.163
7.156
7.131
7.118
7.105
7.095
7.087
4.187
4.161
3.955
3.954
3.929
3.927
3.820
3.804
3.787
3.771
3.753
3.738
2.803
2.802
2.785
2.783
2.772
2.770
2.753
2.752
2.381
2.284
2.266
2.253
2.236
1.254
1.238
-0.000
Ph
Ph
N
Ts
2.00
12.41
1.00
1.02
1.06
1.00
2.98
1.03
3.28
8
7
6
5
4
3
2
1
PPM

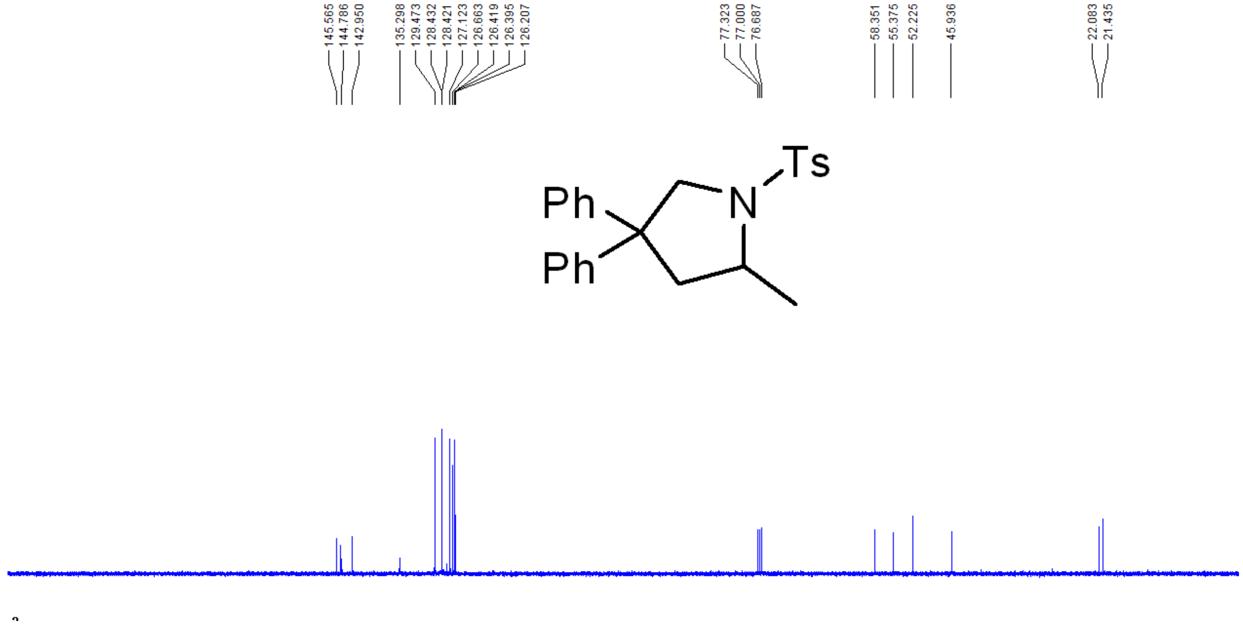

145.565
144.786
142.950
135.298
129.473
128.432
128.421
127.123
126.663
126.419
126.395
126.207
77.323
77.000
76.687
58.351
55.375
52.225
45.936
22.083
21.435
Ph
Ph
N
Ts

实验时间：2013-04-13, 14:56:40
谱图文件:C:\Users\Administrator.PC-20130627LMXK\Desktop\胺氢化\syw-13-12E-PA-2-6-4-0.5-214.dat

实验者:
报告时间：2013-06-28, 18:01:44
积分方法:面积归一法

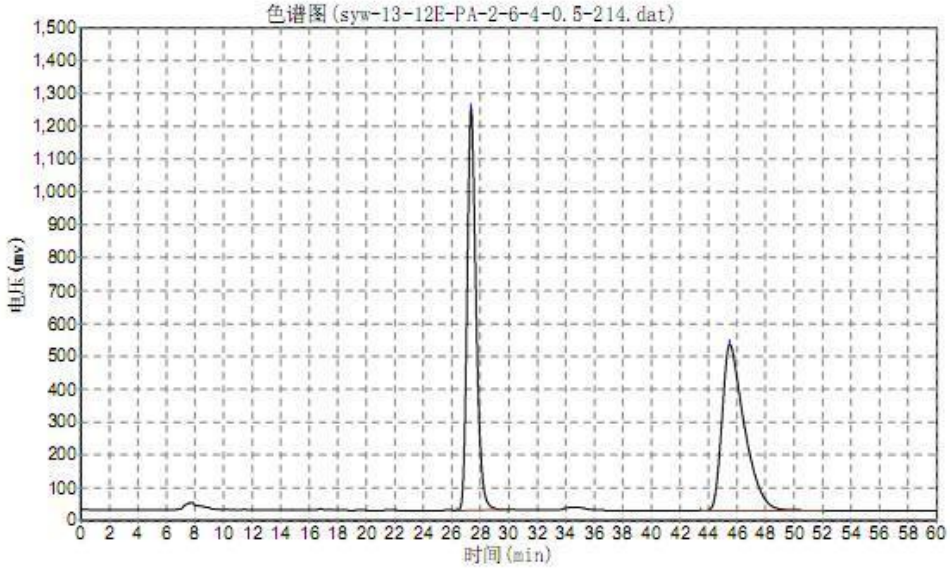

分析结果表

峰号	峰名	保留时间	峰高	峰面积	含量
1		27.353	1223504.375	52971004.000	49.0193
2		45.495	507170.813	55090580.000	50.9807
总计			1730675.188	108061584.000	100.0000

Translation

Peak No.	Peak name	Retention time	Peak height	Peak area	Content
1		27.353	1223504.375	52971004.000	49.0193
2		45.495	507170.813	55090580.000	50.9807
Total			1730675.188	108061584.000	100.0000

实验时间：2013-04-13, 13:30:07
谱图文件:C:\Users\Administrator.PC-20130627LMXK\Desktop\胺氢化\syw-13-12C-PA-2-6-4-0.5-214.org

实验者：
报告时间：2013-06-28, 17:02:34
积分方法:面积归一法

使用仪器类型:气相色谱　　检测器:FID　　进样器:分流

柱温:程序升温

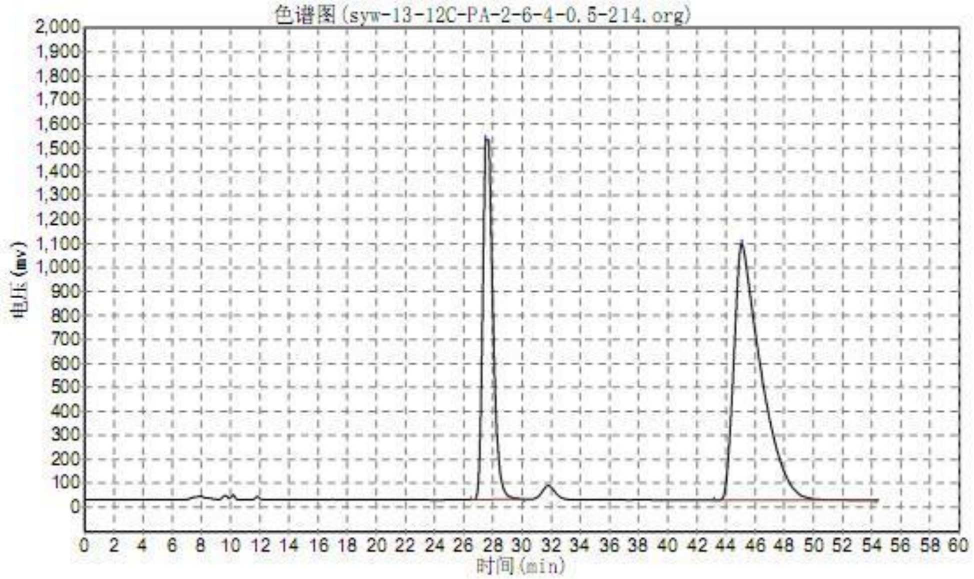

分析结果表

峰号	峰名	保留时间	峰高	峰面积	含量
1		27.495	1498459.875	73299120.000	34.8481
2		45.075	1066793.250	137039824.000	65.1519
总计			2565253.125	210338944.000	100.0000

Translation

Peak No.	Peak name	Retention time	Peak height	Peak area	Content
1		27.495	1498459.875	73299120.000	34.8481
2		45.075	1066793.250	137039824.000	65.1519
Total			2565253.125	210338944.000	100.0000

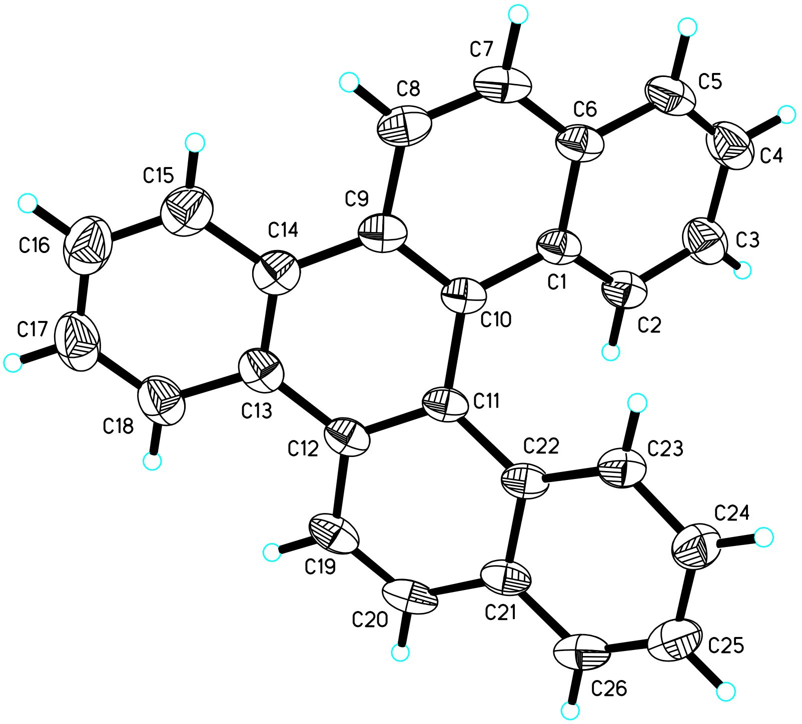

Figure SI-1. The crystal data of compound **23** have been deposited in CCDC with number 920618. Empirical Formula: $C_{26}H_{16}$; Formula Weight: 328.39; Crystal Color, colorless; Crystal Dimensions: 0.212 x 0.156 x 0.113 mm; Crystal System: Monoclinic; Lattice Parameters: a = 5.8492(16)Å, b = 13.351(4)Å, c = 21.102(6)Å, $\alpha = 90^{o}$, $\beta = 91.971(5)^{o}$, $\gamma = 90^{o}$, V = 1646.9(8)Å^3; Space group: P2(1)/n; Z = 4; D_{calc}= 1.324 g/cm^3; F_{000} = 688; Final R indices [I>2sigma(I)]: R1 = 0.0678; wR2 = 0.1630.

References

1. Ooi, T.; Ohmatsu, K.; Maruoka, K. *J. Am. Chem. Soc.* **2007**, *129*, 2410-2411. doi:10.1021/ja063051q

2. Shi, M.; Chen, L.-H.; Li, C.-Q. *J. Am. Chem. Soc.* **2005**, *127*, 3790-3800. doi:10.1021/ja0447255

3. Zhang, J.; Yang, C.-G.; He, C. *J. Am. Chem. Soc.* **2006**, *128*, 1798-1799. doi:10.1021/ja053864z
